# An antimicrobial peptide-resistant minor subpopulation of *Photorhabdus luminescens* is responsible for virulence

**DOI:** 10.1038/srep43670

**Published:** 2017-03-02

**Authors:** Annabelle Mouammine, Sylvie Pages, Anne Lanois, Sophie Gaudriault, Gregory Jubelin, Maurine Bonabaud, Stéphane Cruveiller, Emeric Dubois, David Roche, Ludovic Legrand, Julien Brillard, Alain Givaudan

**Affiliations:** 1 DGIMI, INRA, Univ. Montpellier, Montpellier, France; 2 INRA, UR454 Microbiologie, Saint-Genès-Champanelle, France; 3 MGX-Montpellier GenomiX, c/o IGF, Montpellier, France; 4 Laboratoire d’Analyse Bioinformatique en Génomique et Métabolisme, CEA, Genoscope & CNRS, Evry, France; 5 LIPM, INRA, CNRS, Castanet-Tolosan, France

## Abstract

Some of the bacterial cells in isogenic populations behave differently from others. We describe here how a new type of phenotypic heterogeneity relating to resistance to cationic antimicrobial peptides (CAMPs) is determinant for the pathogenic infection process of the entomopathogenic bacterium *Photorhabdus luminescens*. We demonstrate that the resistant subpopulation, which accounts for only 0.5% of the wild-type population, causes septicemia in insects. Bacterial heterogeneity is driven by the PhoPQ two-component regulatory system and expression of *pbgPE,* an operon encoding proteins involved in lipopolysaccharide (LPS) modifications. We also report the characterization of a core regulon controlled by the DNA-binding PhoP protein, which governs virulence in *P. luminescens*. Comparative RNAseq analysis revealed an upregulation of marker genes for resistance, virulence and bacterial antagonism in the pre-existing resistant subpopulation, suggesting a greater ability to infect insect prey and to survive in cadavers. Finally, we suggest that the infection process of *P. luminescens* is based on a bet-hedging strategy to cope with the diverse environmental conditions experienced during the lifecycle.

Phenotype switching, the ability to switch reversibly between phenotypic states, is a survival strategy commonly used by bacteria confronted with unpredictable environments^1^,^2^. Heterogeneity has been reported for various phenotypes in bacterial pathogens, including persistence in the presence of antibiotic treatment^3^, heterogeneous behavior within biofilms^4^ and the expression of motility determinants^5^,^6^,^7^. A textbook example in bacterial expression is the bistable expression of virulence genes in *Salmonella* Typhimurium^8^, resulting in phenotypically virulent and avirulent subpopulations. A division of labor has been demonstrated for these two subpopulations during *S.* Typhimurium infections^9^.

*Photorhabdus luminescens* (Enterobacteriaceae) is an entomopathogenic bacterium that lives in symbiotic association with the nematode *Heterorhabditis*. The complex invades insect larvae and the nematode regurgitates its bacterial symbiont directly into the hemolymph, the insect equivalent of blood. Once released from the nematode into the open circulatory system of the insect (the hemocoel), *Photorhabdus* interferes with the cellular immunity, which is mediated by circulating hemocytes that engulf the invading bacteria^10^. *P. luminescens* must also evade the peptide-mediated immune response, which involves antimicrobial peptides (AMPs)^11^. Recognition of *Photorhabdus* by the immune system is controlled by the Imd signaling pathway in *Drosophila*, which induces AMP synthesis^12^,^13^. Circulating *P. luminescens* bacteria are initially cleared from the insect hemolymph^12^,^14^, but bacterial septicemia then occurs. Virulence factors also play a role in the death of the insect^10^,^15^. Once the insect host is dead, the bacteria digest the content of the cadaver, which the nematode then uses as a food source to sustain several generations of reproduction^16^. *Photorhabdus* produces a number of antimicrobial molecules that eliminate bacteria that might antagonize the growth of the nematode partner and compete for nutrient resources^10^. Phenotypic switching is a frequent occurrence in the genus *Photorhabdus*^16^. Phenotypic variants generally emerge after prolonged *in vitro* culture of the wild-type strain collected from the nematode^17^. One particular phenotypic variant, the secondary variant, is characterized by changes to many of the traits of the wild-type form (the production of extracellular enzymes, antibiotics). Generally, both the wild-type and secondary variant forms are virulent in insect hosts, but only the wild-type supports nematode growth and development^11^. In the model strain, *P. luminescens* TT01, transcriptomic and proteomic analyses of the wild-type and secondary variants have suggested that phenotypic switching results in extensive cellular reprogramming mediated by transcriptional regulators^14^,^18^. However the emergence of secondary variants has been shown to be unrelated to genomic rearrangements^19^. Another source of phenotypic heterogeneity in *P. luminescens* is switching between the ON and OFF states of expression of the fimbrial maternal adhesion locus (*mad* genes), through an inversion of the promoter region. In the ON state, *mad* genes are transcribed and the bacteria are covered with fimbriae^20^. This form of *Photorhabdus* is called the M form (for “mutualistic”) and it is observed only during symbiosis with the nematode. When the *mad* promoter is in the OFF conformation, the bacteria have no fimbriae and are in P form (for “pathogenic”)^21^. Thus, phenotypic switching is required for the mutualistic relationship between *Photorhabdus* and its nematode host. We describe here a new heterogeneity phenomenon relating to resistance to cationic antimicrobial peptides (CAMPs) that has a determinant effect during the pathogenic phase of insect infection. CAMPs have antibacterial activity mediated by charge interactions with the anionic bacterial surface, predominantly through binding to the acidic lipid A moiety of LPS^22^. In some bacteria, LPS modifications are mediated by proteins encoded by genes regulated by the PhoPQ two-component system^23^. The addition of an aminoarabinose moiety to the lipid A core (a PbgPE-dependent LPS modification) decreases the initial electrostatic attraction by reducing the net negative charge on the outer membrane, thereby playing a role in bacterial virulence by increasing resistance to CAMPs^24^. In *Salmonella,* PhoP indirectly regulates the *pbgPE* operon (also called *pmr* or *arn)*, which encodes the enzymes responsible for this lipid A modification, via another two-component system, PmrAB^24^. The *pbgPE* operon of *Photorhabdus* is required for virulence in *G. mellonella* insects^25^, but the genome of this bacterium contains no *pmrAB* genes^26^. The *Photorhabdus phoP* mutant results in a completely avirulent phenotype in lepidopterans and susceptibility to CAMPs, such as polymyxin B and cecropins A and B^27^. Furthermore, expression of the PhoP-regulated *ail1* gene, which encodes an outer membrane protein, is dependent on Mg^2+^ concentration^28^. Mg^2+^ is also the main *in vitro* inducer responsible for activating PhoPQ in *Salmonella*^29^. We show here that the virulence strategy of *P. luminescens* involves the generation of a subpopulation of bacteria resistant to CAMPs that causes septicemia in insects. The resistant bacteria account for only 0.5% of the wild-type population in *P. luminescens* TT01 during *in vitro* culture. This bacterial heterogeneity depends on PhoP and *pbgPE*.

## Results

### A bacterial subpopulation in *P. luminescens* displays resistance to CAMPs dependent on the *phoP* and *pbgPE* genes

**Figure 1:**
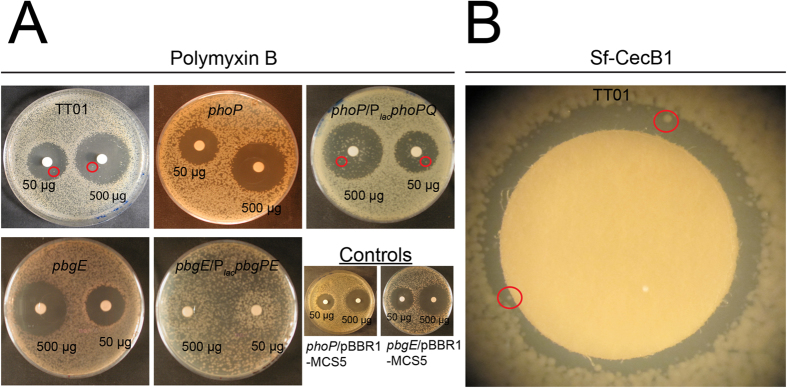
Heterogeneity of CAMP resistance in the TT01 wild-type strain population. (**A**) Bacteria were layered onto agar plates with paper disks loaded with 500 μg and 50 μg of polymyxin B. The antimicrobial resistance patterns of the various strains were determined in agar disk diffusion assays. Polymyxin B created a visible halo for all strains except for *pbgE* /P_*lac*_*pbgPE*, which was completely resistant. (**B**) Cecropin B (Sf cecB1) resistance patterns of TT01 in agar disk*-*diffusion assays. Red circles highlight colonies found within the halos. Experiments were performed at least twice.

We analyzed the resistance profiles to CAMPs of the wild-type population TT01, a *phoP* mutant and its *phoP*/P_*lac*_*phoPQ* complemented strain, a *pbgE* mutant and its *pbgE/*P_*lac*_*pbgPE* complemented strain, by determining minimal inhibitory concentrations (MICs). As previously reported^25^, TT01 was resistant to high doses of CAMPs, whereas the *phoP* and *pbgE* mutant strains were susceptible to very low doses of antimicrobial compounds, such as polymyxin B (Table 1). The wild-type resistant phenotype was restored in the complemented strains (*phoP*/P_*lac*_*phoPQ, pbgE/*P_*lac*_*pbgPE*). The results of this bulk assay suggest that the wild-type population is fully resistant to CAMPs and that this resistance is dependent on the products of the *pbgPE* genes. Unlike MIC determination, agar disc diffusion assays can be used to analyze bacterial resistance to CAMPs at the individual CFU level (Fig. 1A). Surprisingly, only a few colonies of TT01 were able to grow in the halo containing a gradient of polymyxin B concentration, demonstrating that most of the cells of TT01 were susceptible to polymyxin B. By contrast, no resistant clones were observed in the halo for the *phoP* and *pbgE* mutant strains. The *pbgE*/P_*lac*_*pbgPE* complemented strain had a fully resistant phenotype, whereas the *phoP*/P_*lac*_*phoPQ* complemented strain was broadly susceptible, with a profile similar to that of TT01. We also assayed the resistance of TT01 to the synthetic *Spodoptera frugiperda* cecropin B (*Sf*-CecB1), to mimic the antimicrobial activities naturally found in the plasma of insect larvae challenged with bacteria^30^. A minor Sf-CecB1-resistant population of TT01 was also observed in the agar disc diffusion assay (Fig. 1B). However the high MIC of *Sf-*CecB1 (Table 1) made further experiments too costly, we therefore use polymyxin B throughout the study. Finally, the TT01 strain of *P. luminescens* is a mixed population of individuals, most of which are susceptible to CAMPs, with only a small number of bacterial cells displaying resistance dependent on the expression of *phoP* and *pbgPE*.

### CAMP resistance is a new *reversible non*-*genetic phenotype in P. luminescens*

While phenotypic variation phenomena have been described in *Photorhabdus*^16^,^21^, the variant trait (antimicrobial resistance) was not previously reported. Beside polymyxin resistance, we showed that all other tested phenotypes (dye adsorption, the production of extracellular lipases, hemolysins and antibiotics and the ability to generate bioluminescence) were the same as for the wild-type demonstrating a new type of phenotypic heterogeneity. We then quantified the proportions of resistant and susceptible cells in TT01 during bacterial growth in liquid medium, by spreading the bacteria on plates with and without polymyxin B supplementation (Supplementary Figure 1). About 0.5% of the wild-type population was found to be resistant to CAMPs. The reversion phenomenon was then studied from a single colony of polymyxin-resistant clones. After subculturing in broth medium without antibiotic, we yielded a rate of about 1% of resistant bacteria as observed for the wild type showing that the CAMP resistance profile of this subpopulation of cells was reversible *in vitro*. Such survival rate and reversion phenomenon suggest that resistance heterogeneity in *P. luminescens* is not governed by mutations alone. We confirmed the hypothesis of a non-genetic origin of CAMP resistance, by first comparing the sequences of the *pbgPE* and *phoP* loci between the wild-type population and the resistant subpopulation, using cultures established from the same inoculum. Sanger sequencing revealed no differences between the wild-type population and the resistant subpopulation. We also compared the whole-genome sequence of the polymyxin B-resistant subpopulation obtained with SMRT technology to that of the wild-type population; we found no major rearrangement or relevant mutation (Supplementary Table 1). In particular, no inversion of the promoter region of the fimbrial *mad* locus was detected showing that polymyxin-resistant clones are not the previously described M form variant^21^.

### Single-cell analysis of the dynamics of resistance gene expression

**Figure 2:**
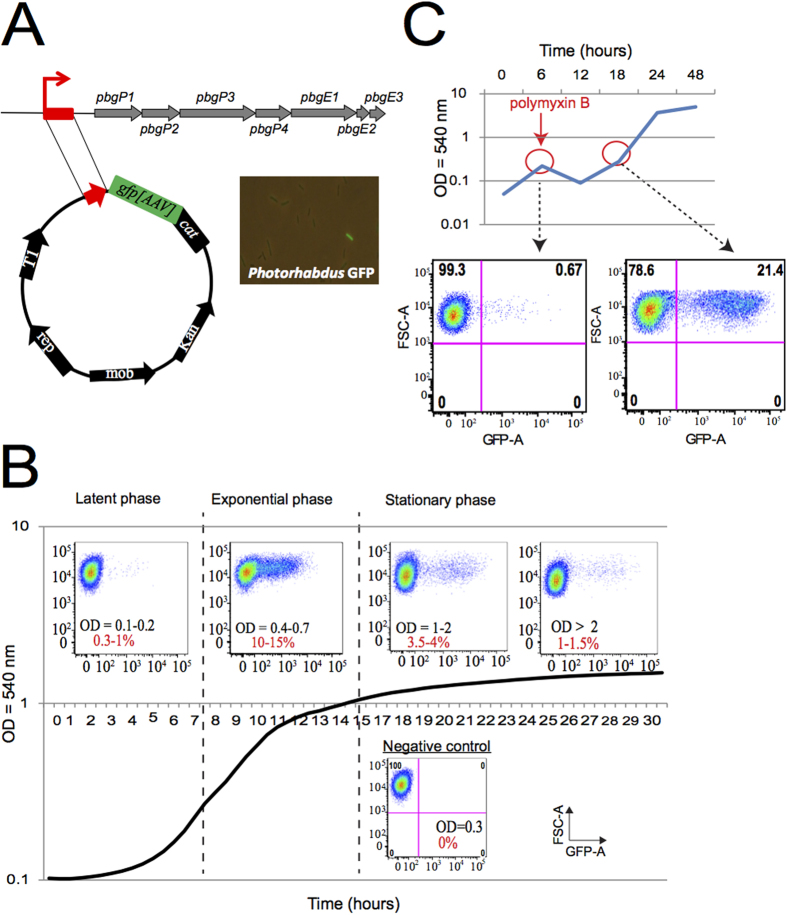
Resistance gene expression after polymyxin B selection at the single-cell level. (**A**) Representative scheme of the P_*pbgPE*_-*gfp*[AAV] transcriptional fusion between the *pbgPE* promoter and a destabilized GFP-encoding gene. A GFP-positive bacterium expressing resistance genes is shown. (**B**) TT01/P_*pbgPE*_-*gfp*[AAV]) was cultured in LB and samples taken over a time course were fixed with formaldehyde and analyzed in a FACS Canto II cytometer. Results are presented as dot plots of side scatter (FSC) against GFP fluorescence intensity. Each dot represents one bacterium. (**C**) TT01/P_*pbgPE*_-*gfp*[AAV] was first cultured in LB. Samples were collected at an OD_540_ of 0.3, before and after the addition of polymyxin B to the culture medium. The representation used here is as in panel B. Only live cells were considered, after treatment with eFluor 660. One representative experiment from more than three independent experiments is shown.

We investigated whether the emergence of the resistant subpopulation was correlated with higher levels of *pbgPE* expression in single cells, by constructing a transcriptional fusion between the promoter region of the *pbgPE* operon and a destabilized GFP-encoding gene named TT01/P_*pbgPE*_-*gfp*[AAV] reporter strain (Fig. 2A). In the absence of CAMP selection pressure (Fig. 2B), the fluorescence of individual cells harvested at various growth phases was heterogeneous, suggesting a lack of synchronization of *pbgPE* gene expression within the population during bacterial growth. The proportion of cells expressing *pbgPE* was highest during the exponential growth phase (10–15%). Cytometry analysis of a population of cells harboring a transcriptional fusion between the promoter of the *pbgPE* operon and a stable GFP-encoding gene (the P_*pbgPE*_-*gfp*[mut3]) revealed that 70 to 80% of the bacteria were GFP-positive during the exponential and post-exponential growth phases, whereas up to 87% of the bacteria in long-term cultures were GFP-positive (data not shown). These data illustrate that most cells were able to activate the *pbgPE* promoter during growth. We then used the same procedure with the destabilized GFP to determine the pattern of fluorescence for the bacterial population during the selection of a resistant subpopulation with polymyxin B (Fig. 2C). The addition of polymyxin B led to a 32-fold increase in the number of living cells producing GFP. The intensity of the GFP signal representing the *pbgPE* expression of the cell (*x*-axis) was not higher in LB than after polymyxin B treatment. These findings are consistent with the selection of a resistant subpopulation by CAMP.

### Characterization of the PhoP regulon in *P. luminescens*

**Figure 3:**
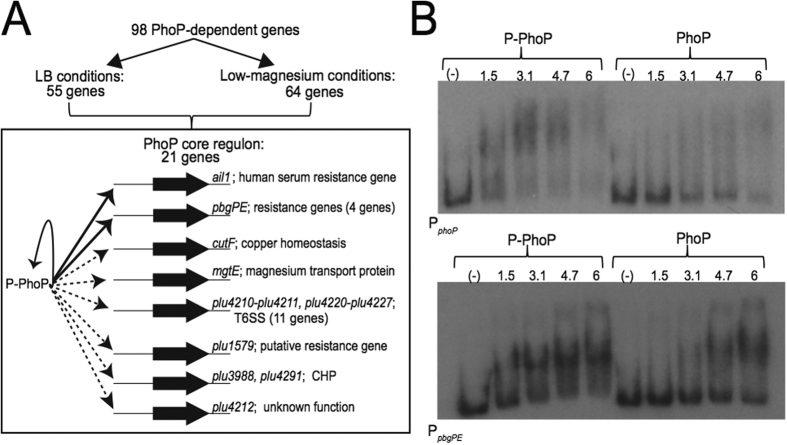
Model summarizing the core PhoP regulon in *P. luminescens.* (**A**) Two sets of PhoP-dependent genes (55 LB and 64 low Mg^2+^) were identified after RNAseq analysis of the gene expression patterns of the TT01 and *phoP* strains in two different sets of growth conditions (LB medium or minimal M9 medium supplemented with 10 μM Mg^2+^). The intersection of the two lists identifies 21 gene markers as the core PhoP regulon (see box). Bold thin arrows indicate direct regulation by the phosphorylated form of PhoP (P-PhoP). Hatched thin arrows indicated that the binding profile is unknown. CHP: conserved hypothetical protein, T6SS: type 6 secretion system. (**B**) Electrophoretic mobility shift assays were carried out to test the binding of the PhoP protein activated *in vitro* with 10 mM acetyl phosphate (P-PhoP) or of the non-activated PhoP-His to the 206-bp *phoP* and 198-bp *pbgPE* promoter regions. The PhoP-His concentrations indicated are in micromoles per liter. We checked that binding was specific, by adding BSA and poly(dI-dC) to the binding buffer to saturate non-specific binding sites.

As PhoP was essential for the emergence of a CAMP-resistant subpopulation, we first characterized the PhoP regulon in *P. luminescens* at the population scale. Growth in the presence of low levels of Mg^2+^ is a triggering signal for PhoP regulon expression^29^. We therefore used RNAseq analysis to compare the gene expression profiles of TT01 and the *phoP* mutant grown either in LB or in a medium containing low Mg^2+^ levels, at mid-exponential growth phase (OD_540_ = 0.3). We used DESeq differential analysis to compare each annotated feature between transcriptomes. We observed significant differences in expression between the *phoP* mutant and TT01 for 55 coding sequences (|log_2_ fold change| ≥1; adjusted *p*-values ≤ 0.005) in LB medium and for 64 coding sequences in low-Mg^2+^ medium (Supplementary Table 2A,B). The PhoP regulatory network, then, consists of 98 genes that are differentially expressed between TT01 and the *phoP* mutant strain (Fig. 3A). Only 26 genes were downregulated by PhoP in both LB and low-Mg^2+^ conditions. We subsequently defined the core PhoP regulon in *P. luminescens* as the 21 differentially regulated genes (*de facto* over-expressed) in both sets of conditions out of the 98 PhoP-dependent genes (Fig. 3A) and six loci were validated by real-time RT-qPCR (Supplementary Table 2C). The core PhoP regulon includes genes involved in CAMP resistance (*pbgP* operon), resistance to host factors (*ail1*, plu1579) and genes encoding conserved hypothetical proteins of unknown function. We also found 11 genes belonging to a locus encoding putative components of a type 6 secretion system in TT01 (T6SS-4_TT01_: plu4198-plu4227) (Fig. 3A). For the entire PhoP regulon, we identified six additional genes from the T6SS-4_TT01_ locus and four genes from the T6SS-3_TT01_ locus (plu3259-3262) as differentially regulated (Supplementary Table 2A). T6SS are widely thought to be involved in cellular interactions between bacteria or between bacteria and eukaryotes. Their function in *Photorhabdus* has yet to be determined, but both these roles are consistent with the lifestyle of this bacterium^16^.

PhoP is a transcriptional regulator that mostly upregulates its network through direct or indirect interactions. The phosphorylated form of PhoP can bind directly to the promoter region of the *ail1* gene^28^, a member of the core PhoP regulon. We investigated the architecture of the PhoP network further, by exploring the interactions of PhoP with the promoter sequences of the *pbgPE* and *phoPQ* operons, both of which are required for the emergence of the resistant subpopulation. We carried out electromobility shift assays (EMSA) to compare the interaction profiles of different amounts of PhoP protein with the *pbgPE* and *phoP* promoter regions. A shift was observed for both promoters after the addition of phosphorylated PhoP-His (Fig. 3B). No such shift was observed after incubation with unphosphorylated PhoP-His at low concentration (below 4.7 μM). This specific binding to the promoter regions of *phoP* and *pbgPE* confirmed that the phosphorylated form of PhoP is the active isoform and revealed that PhoP is involved in positive feedback control of its own expression (Fig. 3A).

### Concomitant overexpression of genes involved in resistance, virulence and bacterial antagonism in the polymyxin B-resistant subpopulation

**Figure 4:**
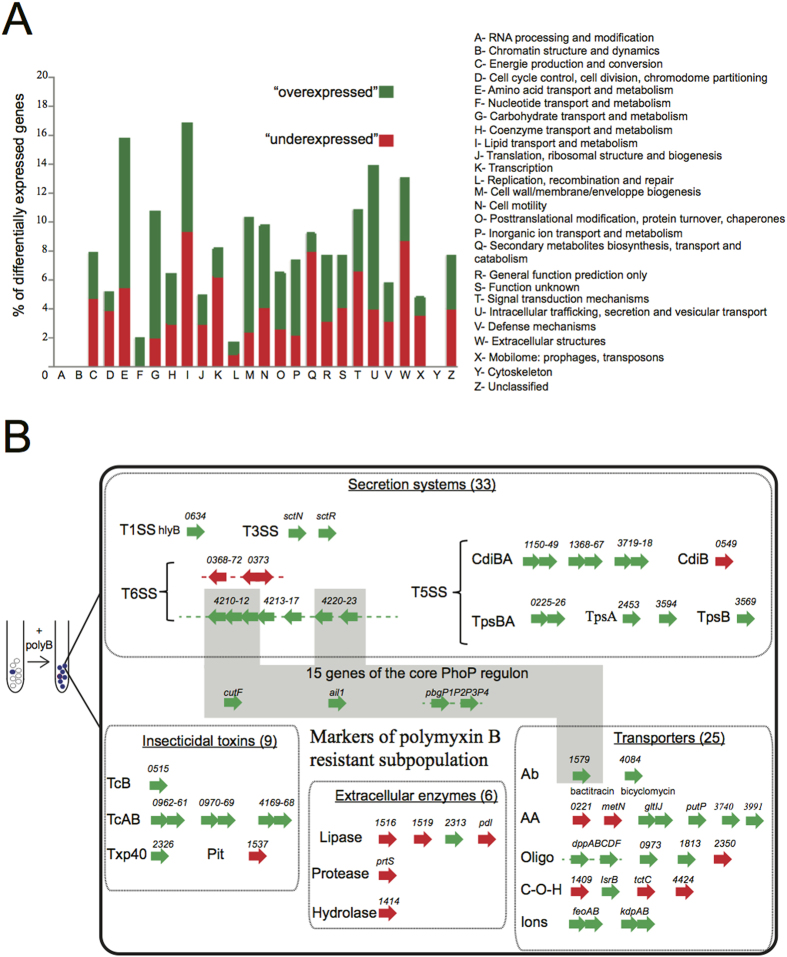
Identification of marker genes for the polymyxin B-resistant subpopulation. (**A**) Classification by COG (cluster of orthologous class) annotation of the 445 genes displaying a change in expression after the addition of polymyxin B (polymyxin B gene set) according to the 2014 update (ftp://ftp.ncbi.nih.gov/pub/COG/COG2014/static/lists/homeCOGs.html). The percentage of genes from each COG class differentially expressed between the polymyxin B-resistant subpopulation and *Photorhabdus luminescens* TT01 genes is shown. (**B**) Four functional groups of representative marker genes for the polymyxin B-resistant subpopulation. The number of genes in each group is indicated in brackets. Genes are represented as arrows with their name or their label number. Overexpressed and underexpressed genes are indicated in green and red, respectively. The gray area corresponds to genes from the core PhoP regulon. AA: amino acids; C-O-H: carbohydrates; Ab: antibiotics; oligo: oligopeptides.

We investigated the gene expression profiles of the subpopulation defined on the basis of CAMP resistance, by extracting RNA from bacteria grown in LB medium and harvested at an OD_540_ of 0.3–0.4 before (control) and after the addition of polymyxin B (10–12 hours of exposure). We observed significant differences (|log_2_ fold change| ≥1; adjusted *p*-values ≤ 0.005) in expression between the two sets of conditions for 445 genes (Supplementary Table 3). We found that 208 genes were underexpressed and 237 genes were overexpressed in the resistant subpopulation relative to the control (*i.e*., the same clonal population before the addition of polymyxin B) (Supplementary Table 3B,C). COG annotations and representations of percentages by COG class in TT01 genome (Fig. 4A) indicated that the polymyxin B-selected regulon was not massively biased towards one specific COG class. Nevertheless, we observed a slight enrichment in genes encoding proteins involved in amino-acid transport and metabolism (E), lipid transport and metabolism (I), intracellular trafficking, secretion and vesicular transport (U) and extracellular structures (W). Interestingly, four large families of proteins previously described in *P. luminescens*^26^ were over-represented (Fig. 4B). First, components of secretion systems were frequently found to be differentially expressed and some of these systems have been described as bacterial contact-dependent delivery systems^31^. One remarkable marker of the polymyxin B-resistant subpopulation is the family of proteins secreted by the two-partner secretion (TPS) pathway (T5SS). Actually, six of the ten genes encoding TpsA (the exoproteins) and six of the eight genes encoding the TpsB (β-barrel protein pores) were differentially expressed. For *tpsAB* genes, the expression of *phlAB,* encoding a protein involved in secretion of the hemolysin PhlA in *P. luminescens*^32^, was similar in the presence and absence of polymyxin B, whereas most of the *cdiAB* genes (seven of eight *cdi* genes) encoding a new functional family of contact-dependent inhibition (CDI) system^33^ were representative markers of the polymyxin B subpopulation (Fig. 4B). Moreover, 12 PhoP-dependent genes encoding the T6SS-4_TT01_ were overexpressed markers in the resistant subpopulation, whereas six genes from another T6SS locus (T6SS-1_TT01_: plu0335-plu0373) were underexpressed in the resistant subpopulation (Fig. 4B). Similarly, 25 genes encoding components of transporters of small molecules were differentially expressed between the resistant subpopulation and the wild-type population. The other overrepresented proteins were large multimeric protein complexes from the insecticidal toxin complex (Tc) family^34^. Seven genes encoding TcA or TcB subunits were overexpressed in the resistant subpopulation, suggesting higher virulence for the resistant subpopulation in insects. Finally, five extracellular enzymes classically described as causal factors of insect cadaver degradation^35^ or enhancers of Tc toxins, were underexpressed in the resistant subpopulation. We also found that a large proportion of the genes of the PhoP regulon were overexpressed in the resistant subpopulation: 37 of the 55 PhoP-dependent genes in LB broth (Supplementary Table 3C) and 15 of the 21 genes from the core PhoP regulon (Fig. 4B; Supplementary Table 3C). Moreover we showed by RT-qPCR that the main signature markers (PhoP-dependent or not) are also present in polymyxin-resistant clones grown without polymyxin B (Supplementary Table 3D) demonstrating that the presence of the antibiotic is not required to the differential expression of signature markers. Thus, the polymyxin B-resistant subpopulation mainly displayed an upregulation of genes involved in CAMP resistance, virulence (such as *tc*) and bacterial antagonism (such as *cdi*), suggesting an enhanced ability to infect insects and to outcompete saprophytic bacteria in cadavers.

### The resistant subpopulation is responsible for killing insects

**Figure 5:**
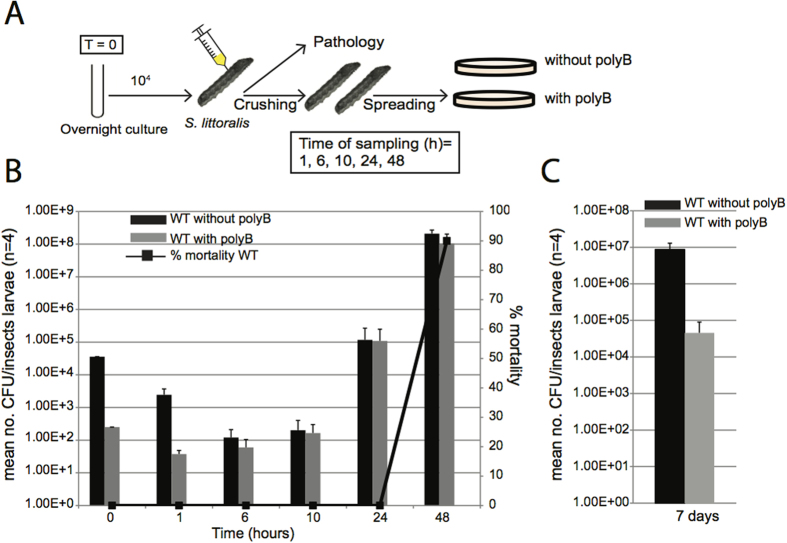
The resistant subpopulation is the major population present during septicemia in insects. (**A**) Representative diagram of the experimental procedure. **(B**) Bacterial growth and insect larval mortality following the injection of *P. luminescens* TT01 into *Spodoptera littoralis.* We injected 3 × 10^4^ bacteria into each larva at time zero. The histogram shows the mean numbers of CFU recovered from four larvae per time point, after plating on nutrient agar (black bars) or nutrient agar supplemented with polymyxin B (gray bars). The error bars indicate the standard error of the mean. The lines indicate larval mortality rates (for 20 larvae per treatment). One representative experiment from more than three independent experiments carried out is shown. (**C**) The procedure and representation are as in panel B, except that the insect cadaver extracts were plated on agar seven days after injection.

The observation of a *Photorhabdus* population containing cells in which virulence and resistance genes were pre-activated *in vitro* led us to study the fate of this resistant subpopulation in insects through studies of the *in vivo* growth kinetics of the *P. luminescens* TT01 strain. At various time points after injection, we quantified the cells of the resistant subpopulation by plating extracts of crushed insect larvae (Fig. 5A). As previously described^14^, the total number of living TT01 CFU decreased by two to three orders of magnitude during the first few hours after injection, corresponding to the clearance phase. Between 6 to 10 hours post infection (hpi), the total population of living cells consisted entirely of resistant bacteria (Fig. 5B). *Spodoptera* larvae synthesize AMPs 3 to 9 hours post injection^36^,^37^, a time period corresponding perfectly to the observed clearance phase. During the septicemia phase, when the insects were dying, the resistant subpopulation outcompeted the susceptible population, highlighting the dependence of septicemia on the multiplication of resistant bacteria. No insect death and septicemia was observed after the injection of the *phoP* or *pbgE* mutant strains (see Supplementary Figure 2) and as expected, no resistant bacteria were observed (data not shown). To confirm that the resistant subpopulation consisted of pre-adapted infectious cells, we injected a selected polymyxin B-resistant subpopulation into insects at a lower dose (10^3^ bacteria per insect). The LT_50_ was reached four hours earlier than for the TT01 wild-type population (see Supplementary Figure 3). We also assessed the reversibility of the resistant phenotype in insects after long-term incubation *in vivo*. The proportion of resistant cells in the population present in the insect cadaver returned to its initial value after seven days (Fig. 5C).

## Discussion

We identified phenotypic and transcriptional heterogeneity underlying individual variation in resistance to CAMPs in subpopulations of *P. luminescens*. The high rate with which resistance emerged and its rapid reversion in the CAMP-resistant subpopulation suggested that the variation was not genetic. Genome sequence analysis identified no causal mutation related to the polymyxinB-resistance phenotype, suggesting that the resistance was instead epigenetic. Differences between cells in isogenic sbacterial populations may reflect noise due to central cellular processes^1^. We have shown in the sister species of this entomopathogenic bacterium, *Xenorhabdus*^5^, that feedback loops in regulatory networks lead to bimodal gene expression or bistability, as described elsewhere^38^. We also found that the emergence of *P. luminescens* resistant individuals is driven by the PhoP-PhoQ two-component regulatory system and the *pbgPE* resistance genes encoding proteins involved in LPS modifications^25^. Consistent with previous reports^27^, we found that PhoP regulated its own transcription via a positive feedback loop and we observed direct binding of phosphorylated PhoP to the promoter region of the *pbgPE* operon. However, no conserved PhoP box consisting of a repeated hexameric sequence^39^ was identified in the 5′UTR of these two genes. In *Salmonella enterica*, the positive feedback loop of the *phoPQ* operon is required for a surge in the activity of the PhoP/PhoQ system^40^. In *P. luminescens*, the ectopic expression of *phoPQ* genes and bacterial growth in inducing conditions (low Mg^2+^ conditions) did not significantly increase the proportion of polymyxin B-resistant individuals in the total population. Furthermore, the feedback loop controlling *phoP* expression was unable to stabilize the noisy pattern of *pbgP* expression in the presence and absence of CAMP. Despite the lack of synchronization of PhoP-dependent *pbgPE* gene expression during the growth of *P. luminescens,* appropriate levels of expression, at the right time, can be achieved randomly in certain individuals, facilitating thereby the modifications to LPS involved in CAMP resistance. The mechanisms underlying the erratic expression of *pbgPE* genes have yet to be characterized. Finally, this non-genetic variation in *P. luminescens* displayed some degree of heritability, as the production of a sufficiently large progeny allowed colony formation in the presence of CAMP selection, suggesting a role for epigenetic variation. It is noteworthy that PhoP-mediated LPS modifications also occur in a subset of *S. enterica* during infection and this bacterial heterogeneity is sufficient to drive radically different host immune responses^41^. Another successful strategy of antibiotic resistance used by subpopulations of bacterial cells involves the generation of persister cells, which cannot multiply in the presence of antimicrobial compounds^3^. However, transient dormancy states cannot confer resistance to cationic peptides acting like surfactants. A recent study described an *Enterobacter cloacae* isolate harbouring a minor subpopulation highly resistant to colistin (polymyxin E)^42^. As shown here with polymyxin B, this subpopulation become predominant in colistin and returned to baseline after antibiotic removal and was also dependent on PhoQ. Authors proposed to refer to this resistance phenomenon described in *E. cloacae* as “clonal heteroresistance”. However, excepted a higher levels of the lipid A modification genes such as *arnB (pbgP1* synonym), signature markers described in polymyxin-resistant subpopulation of *Photorhabdus* (such as T6SS or T5SS upregulation) were not found in colistin-resistant *E. cloacae*. Other clinical studies have also reported the preadaptation of certain bacterial cells to antibiotic challenge in the absence of mutation. So-called “adaptive” resistance typically involves transient changes in the expression of genes encoding porins or efflux pumps, increasing the chances of the isogenic bacterial population surviving antibiotic treatment^43^. For instance, single-cell heterogeneity in expression of the porin gene *ompC* confers adaptive resistance to kanamycin in *Salmonella*^44^. The resistance phenotype described here is a similar, transient phenomenon, but the mechanism involved is different. Moreover we also showed here that the emergence of polymyxin resistance in *P. luminescens* is a phenomenon different than phenotypic variation previously described in this genus.

One of the most striking findings of this study is the selection of the transient resistant state during infection, rendering the subpopulation of cells resistant to CAMPs virulent in insect larvae. Indeed, *P. luminescens* kills insects through bacteremia, and the LT_50_ of *Photorhabdus* has been shown to be directly correlated with its rate of growth *in vivo*^45^. We found that the observed clearance of circulating bacteria from the insect hemolymph^12^,^14^ was consistent with the destruction of most of the bacterial cells susceptible to the CAMPs produced by the insect early in infection. In particular, attacin C and drosocin were strongly upregulated after *Photorhabdus* infection^13^. The subsequent growth of the CAMP-resistant population leads to septicemia and insect death. After the infection stage, reversibility of the resistant phenotype, with a return to the initial proportions of resistant cells was observed after long-term incubation in insect cadavers, consistent with reports that CAMPs may be degraded by proteases, produced by *P. luminescens*^46^. The reversible phenotypic variation in *Photorhabdus,* conferring an adaptive resistance phenotype on a minor subpopulation, may clearly be seen as a bet-hedging strategy^47^. Our findings are consistent with the hypothesis that bet-hedging is potentially advantageous in harsh and unpredictable environments^48^. One question that remains to be addressed is why the infection strategy is based on bet-hedging, rather than direct environmental sensing in *P. luminescens*. Indeed, *Photorhabdus* has to cope with very different environments imposed by the host immune system. The nematode hosts of these bacteria target a wide range of insects^49^. Each insect family has its own defense system, with different patterns and the production of different families of CAMPs (for review see^50^). We therefore suggest that *P. luminescens* survival is partly dependent on a non-specific mechanism of resistance to CAMPs, involving LPS modifications and that the expression of the resistance phenotype is noisy because the insect-host environmental signals are highly diverse and unpredictable due to the diversity of insect prey^49^. We also found that the selection mediated by polymyxin B *in vitro* or by insect CAMPs early in infection resulted in the generation of individuals strongly expressing antibiotic resistance genes. As expected, the overexpression of *pbgPE* and of the other main PhoP-dependent genes from the PhoP core regulon was identified as a marker of this selection. However, with the exception of two genes encoding putative transport proteins (Fig. 4), very few other antimicrobial peptide resistance genes were selected. In addition, our RNAseq data revealed no induction of regulon involved in stress responses. Interestingly, detailed analysis revealed enrichment in genes encoding type 5 and 6 secretion systems. Complex interplay has already been reported between LPS O-antigen structure in *Pseudomonas aeruginosa* and polymyxin B, a membrane-disrupting product, with the induction of T3SS and T6SS expression^51^,^52^. Like T6SS, the CdiAB family of TPS proteins mediates interbacterial competition in a contact-dependent manner^53^. Indeed, *cdi* genes are highly representative markers of the polymyxin B-resistant subpopulation. The CdiAB toxin family is overrepresented in the genus *Photorhabdus*^33^, but its function remains unknown. By analogy to other bacteria^53^, the overexpression of contact-dependent delivery systems in pre-adapted resistant *P. luminescens* may confer a substantial growth advantage in kin competition (i.e. with other nematode-associated bacterial strains) in cases of multiple nematode infestations. The cost of bet-hedging is related to the generation of types poorly adapted to the current conditions. The majority population of susceptible bacteria is probably avirulent in lepidopterans, as reported for *pbgE* and *phoP* mutants (Supplementary Fig. 2). This population may also display other impairments of the expression of secretion systems, transporters and insecticidal toxins. The CAMP-susceptible subpopulation is probably at an advantage under one indeterminate condition, and can outcompete CAMP-resistant individuals under a different set of conditions. Remarkably, it is likely that the majority population of susceptible bacteria produces larger amounts of extracellular enzymes than the resistant subpopulation. This susceptible population may, therefore, be better adapted to the next phase of the *Photorhabdus* life cycle, involving the degradation of insect cadavers to support nematode reproduction.

The phenotypic variation in *P. luminescens* underlies its virulence and the rapid transition from an exponentially growing pathogenic and resistant state to a post-exponential growth-stage form able to promote its own growth and that of its nematode host. Low nutrient concentration triggers the last phase of the *Photorhabdus* lifecycle: colonization of the nematode host and transmission to the next insect prey. The entry into stationary growth phase also coincides with the emergence of another variant form previously described^21^: the mutualistic form able to colonize the infective juvenile stage of the nematode. In conclusion, both studies clearly illustrate that phenotypic switching fully controls the pathogenic and mutualistic interactions between *Photorhabdus* and its invertebrate hosts.

## Methods

### Bacterial strains, plasmids, and growth conditions

The strains and plasmids used in this study are listed in Supplementary Table 4. *P. luminescens* strains were routinely grown at 28 °C in Luria-Bertani (LB) or Mueller-Hinton broth (Biokar), nutrient agar medium (Difco), or NBTA agar^54^. *Photorhabdus* was also grown in M9 liquid medium supplemented with 0.1% casamino acids, 0.41 mM nicotinic acid, 9.1 mM sodium pyruvate, 0.1 mM CaCl_2_ and 0.2% glycerol, with various concentrations of MgSO_4_ (10 μM for low Mg^2+^ conditions and 10 mM for high Mg^2+^ conditions). When required, antibiotics were used at the following final concentrations: polymyxin B (polyB), 100 mg.l^−1^; kanamycin, 20 mg.l^−1^; gentamicin, 15 mg.l^−1^; and erythromycin 15 mg.l^−1^.

### Antibacterial activity

*In vitro* susceptibility tests to determine MICs were performed by the broth microdilution method, according to guidelines^55^. Stock solutions of colistin methane sulfonate (Sigma) and polymyxin B (Sigma) were diluted in sterile water to obtain concentrations of 20 and 50 mg.l^−1^, respectively. Stock solutions of 0.4 mg.l^−1^ cecropin A and B (Sigma) were prepared in sterile water. Synthetic *S. frugiperda* cecropin B (CecB1)^30^ was prepared in distilled water, to yield a concentration of 0.5 mg.l^−1^. Antibiotics were then added directly to 96-well microtiter plates in serial two-fold dilutions. We dispensed 10^4^ bacteria grown to an OD_540_ of 0.6–0.8 into each microdilution well. MICs were determined by eye after incubation in microtiter plates containing Mueller-Hinton broth (Biokar) at 28 °C for 48 h.

### Electrophoretic mobility shift assays (EMSA)

The promoters of *phoP* and *pbgP1* were amplified by PCR from the genomic DNA of TT01 with the primers (Supplementary Table 5), and purified with the High Pure PCR Product Purification kit (ROCHE). The 5′ ends of the DNA were labeled with [γ-^32^P] ATP and T4 polynucleotide kinase (Promega). The PhoP-His protein was purified and phosphorylated *in vitro* by incubation with acetyl phosphate^56^. Radioactive DNA probe (2000 cpm.ml^−1^), 200 ng of poly(dI-dC)-poly(dI-dC) (SIGMA) and various amounts of PhoP-His were mixed in binding buffer (50 mM Tris-HCl pH 8, 50 mM KCl, 50 μg.ml^−1^ BSA), in a total volume of 20 μl, and incubated for 20 minutes at room temperature. The mixture was then loaded onto a native 6% (w/v) polyacrylamide TBE precast gel (Invitrogen) and subjected to electrophoresis in 1% TBE (Tris-borate-EDTA) buffer for 1 h at 100 V. Radioactive species were detected by autoradiography. PhoP-His was activated by *in vitro* phosphorylation with acetyl phosphate^57^.

### Molecular techniques and RNA preparation

DNA manipulations were carried out as previously described^58^. Plasmids were introduced into *E. coli* WM3064 (Supplementary Table 4) by transformation and transferred to *P. luminescens* by filter mating^32^. All constructs were sequenced by Eurofins MWG Operon. The primers used (Eurogentec) are described in Supplementary Table 5. Total RNA was extracted and purified with the RNeasy miniprep kit (Qiagen), including a DNase I treatment step. For each RNA preparation, we assessed DNA contamination by carrying out a control PCR. The quantity and quality of RNA were assessed with an Agilent 2100 Bioanalyzer with the RNA 6000 Nano LabChip kit. RNA was prepared from a culture at an OD_540_ of 0.3–0.45 before and after the addition of polymyxin B to the medium (when the OD_540_ had returned to 0.3). Samples were differentially analyzed in the *phoP* mutant and the wild-type TT01 strain grown in LB or low Mg^2+^ conditions or before and after adding polymyxin B (six independent biological replicates per strain) and pooling equal amounts of total RNA from three replicates of the same strain together. We thus generated two biological samples per strain, which were subjected to two successive rounds of ribosomal RNA depletion with the Microbe Express kit (Ambion) before RNA Sequencing.

### Single-molecule real-time (SMRT) DNA sequencing and mutation analysis

Genomic DNA was extracted from bacteria grown in LB or LB plus polymyxin B and harvested at an OD_540_ of 0.3–0.45 with the QIAamp DNA Mini Kit (Qiagen). An additional purification was performed with the DNA Clean-up kit (MoBio Laboratories). The DNA libraries were prepared according to PacBio guidelines and sequenced on two PacBio SMRT cells on a Pacific Biosciences RSII instrument (Genome Québec, Montréal, Canada). The raw data were processed with the PacBio SMRT Analysis Suite (version v2.3 p3). The reads were assembled *de novo*, with the high-quality Hierarchical Genome Assembly Process HGAP.3 algorithm. The assembled genomes were deposited in the Microscope platform database^59^. No major rearrangements were observed with progressiveMauve 2.1.0.a1^60^. Conserved Synteny LinePlot revealed 100% conservation of synteny groups between the two genomes studied, with a synton size ≥3 and the *P. luminescens* TT01 NC_005126 genome as the reference (data not shown). We used two programs for the detection of SNPs, insertions and deletions: GATK v3.3.0 (The Genome Analysis Toolkit^61^) which was used to map PacBio reads onto the NC_005126 reference genome, and LAST v712^62^, which is based on the alignment of the NC_005126 reference genome and the two HGAP.3 assembled genomes. Regions of poor quality displaying homopolymers ≥4 nucleotides in length or discordant multiple alignments (i.e. mapping several times onto the reference genome, with one correct mapping and one (or several) displaying <100% homology) were considered irrelevant and discarded. With 20% of its genome consisting of repeats, *P. luminescens* TT01 NC_005126 has been identified as a genome with a particularly high level of repeat coverage.

### RNA Sequencing

The RNA-seq libraries were prepared with the TruSeq^®^ Stranded mRNA sample prep kit (Illumina). Samples depleted of rRNA were fragmented and reverse-transcribed with random hexamers, Superscript II (Life Technologies) and actinomycin D. During the generation of the second strand, dTTP was replaced with dUTP. Double-stranded cDNAs were adenylated at their 3′ ends before ligation with Illumina indexed adapters. Ligated cDNAs were amplified by 15 cycles of PCR and purified with AMPure XP Beads (Beckman Coulter Genomics). Libraries were validated with a DNA1000 chip (Agilent) and quantified with the KAPA Library quantification kit (Clinisciences). Twelve libraries were pooled in equimolar amounts in a single lane and were sequenced on a HiSeq2000 machine, with the single-read protocol (50 nt). Image analysis and base-calling were performed with Illumina HiSeq Control Software and the Real-Time Analysis component. Demultiplexing was performed with Illumina sequencing analysis software (CASAVA 1.8.2). Data quality was assessed with FastQC from the Babraham Institute and the sequencing analysis viewer (SAV) from Illumina software.

### RNAseq analysis

High-throughput transcriptomic sequencing data were processed with a bioinformatic pipeline implemented at the Microscope platform^59^. The reads were mapped onto the *P. luminescens subsp. laumondi* TT01 genome sequence (EMBL accession number: BX470251) with BWA software (v. 0.7.4)^63^. We then used SAMtools (v.0.1.12)^64^ to lower the false-positive discovery rate and to extract reliable alignments from BAM-formatted files. The number of reads matching each genomic object harbored by the reference genome was then calculated with the Bioconductor-GenomicFeatures package^65^. For reads matching several genomic objects, the count number was weighted so as to keep the total number of reads constant. Finally, we used the Bioconductor-DESeq package^66^ with default parameters to analyze raw count data and to evaluate differential expression between conditions. In more details, we used the False positive Discovery Rate (FDR) method (variance estimate for each gene with the Negative Binomial distribution followed by a per gene Wald-test generating p-values that were adjusted by the method^67^. Between 11 and 20 million Illumina sequences (50-base reads) were obtained for each sample and between 15 and 40% of high-quality sequences mapped to at least one site in the reference genome. The complete dataset from this study has been deposited in the GEO database, under accession number GSE76559.

### RT-qPCR analysis

RT-qPCR was performed as previously described^5^. The data for each sample are expressed relative to the level of *recA,* using REST software 2009^68^. This method quantified the expression of a target gene relative to that of a reference gene, for comparisons of TT01 with the *phoP* mutant in different growth conditions and for comparisons of TT01 and the polymyxin B resistant subpopulation before and after the addition of polymyxin B.

### Estimation of the size of the resistant subpopulation

Agar disk diffusion assays were performed as follows. An exponentially growing culture was diluted in Mueller-Hinton medium and 1 ml, corresponding to a total of 10^3^ CFU, was spread on Mueller-Hinton agar plates. The excess medium was removed and the plates were dried for 5 to 10 minutes. We placed paper disks on the surface of the plates, and these disks were infiltrated with 500 μg or 50 μg of polymyxin B in a maximum of 10 μl. The plates were incubated at 28 °C and observed after incubation for 48 h. We also determined the proportion of the total population displaying resistance *in vitro* by counting CFUs on nutrient agar plates. Samples from the same culture of TT01 were diluted and at least three dilutions were spread on plates with nutrient agar or nutrient agar supplemented with polymyxin B (final concentration: 100 μg.ml^−1^) to isolate the resistant subpopulation. Samples were collected during bacterial growth, at each key point (lag, exponential and stationary phases). Colonies were counted 48 h after incubation at 28 °C.

### Construction of plasmids expressing *gfp*[AAV] and *gfp*[mut3] under the control of the *pbgPE* promoter

We used a method similar to that reported in a previous study^56^ to construct plasmids expressing the reporter gene *gfp*[AAV] under the control of the *pbgPE* or *lac* promoter. We constructed P_*pbgPE*_-*gfp*[AAV] as follows. Briefly, a DNA fragment corresponding to the *pbgPE* promoter region (198 bp) was amplified by PCR from TT01 genomic DNA with primers containing a *Kpn*I or *Xba*I restriction site (Supplementary Table 5). The PCR products were digested and inserted into the corresponding sites of pPROBE-*gfp*[AAV]. We constructed P_*pbgPE*_-*gfp*[mut3] by replacing the *gfp*[AAV] from P_*pbgPE*_-*gfp*[AAV] with the *gfp* mut3 gene (PCR amplification from pBB-KGFP). Finally, P_*pbgPE*_-*gfp*[AAV] and P_*pbgPE*_-*gfp*[mut3] were transferred into the TT01 wild-type strain by mating.

### Flow cytometry analysis of *pbgPE* expression in individual bacterial cells

The cultures were standardized, with a starting OD_540_ of 0.05 in 100 ml of LB medium supplemented with kanamycin. For kinetic analyses, samples were washed once with PBS (without calcium and magnesium) and the bacteria were fixed by incubation in 2% formaldehyde in PBS for 15 minutes at room temperature. For analysis of the resistant subpopulation, samples were collected at an OD_540_ of 0.3 before the addition of 100 μg.ml^−1^ polymyxin B, and then again after 10 to 12 hours of growth in the presence of polymyxin B. For live-dead staining, samples were washed once and the bacteria were incubated with Hoechst 33342 stain (final concentration: 3 μg.ml^−1^) for 15 minutes, washed twice and then incubated with Fixable Viability Dye eFluor 660 (eBioscience), diluted 1:1,000 in PBS, at 4 °C for 30 minutes before fixation in formaldehyde. All samples were analyzed in a FACS Canto II flow cytometer (BD Bioscience)^5^ for GFP quantification and live-dead analysis. Data were captured for 30,000 bacteria per sample and the raw data were analyzed with FlowJo version 8.8.6 software (TreeStar). Compensation was performed, if required. Only live cells were counted in GFP analysis (Hoechst-positive and eFluor 660-negative bacteria).

### *In vivo* pathogenicity assays

The common cutworm, *Spodoptera littoralis*, was reared with a photoperiod of 12 h on an artificial diet at 23 °C. Fifth-instar larvae were selected and surface-sterilized with 70% (vol/vol) ethanol before injections. To monitor survival curves over time, we injected 10^3^ CFU of exponentially growing bacteria from cultures supplemented with antibiotics if necessary (polymyxin B 100 μg.ml^−1^) into groups of 20 larvae. Treated larvae were incubated individually for up to 96 h, and the time at which the insects died was recorded. Bacterial concentrations were determined by counting CFUs after the plating of dilutions on nutrient agar. Statistical analysis was performed to compare survival experiments. Signed-rank tests (Wilcoxon test) were used to compare mortality patterns, as previously described^69^. For the analysis of bacterial growth kinetics and clearance in insect larvae, we injected more bacteria than previously used for the pathogenicity assays (about 5 × 10^4^ bacteria from overnight cultures) into each of 60 fifth-instar larvae. We used 40 larvae to monitor bacterial growth in hemocoel, respectively. Four groups of two larvae for each time point were surface-sterilized with 70% (vol/vol) ethanol, crushed in 3 ml LB medium with a TissueLyzer II (Qiagen) and centrifuged at 400 × *g* for two minutes to remove larval debris. The extract was then suitably diluted and the density of bacteria present was determined by counting CFUs for dilutions plated on NBTA nutrient agar supplemented with 15 μg.ml^−1^ erythromycin in order to avoid the growth of other bacteria than *Photorhabdus* that is naturally Ery^R^. We evaluated the concentration of bacteria in the resistant subpopulation by adding 15 μg.ml^−1^ erythromycin and 100 μg.ml^−1^ polymyxin B to the plates.

## Additional Information

**How to cite this article:** Mouammine, A. *et al*. An antimicrobial peptide-resistant minor subpopulation of *Photorhabdus luminescens* is responsible for virulence. *Sci. Rep.* **7**, 43670; doi: 10.1038/srep43670 (2017).

**Publisher's note:** Springer Nature remains neutral with regard to jurisdictional claims in published maps and institutional affiliations.

## Supplementary Material

Supplementary Information

## Figures and Tables

**Table 1:** MICs* of four CAMPs for the *P. luminescens* strains.

Strains	MIC (μg.ml^−1^)			
	Colistin	Cecropin A	Cecropin B (*S. frugiperda*)	Polymyxin B
TT01	>10,000	>25	>50	>250
*phoP*	20	0.8–1.6	6–12	1–3
*phoP/*P_lac_PhoPQ	>10,000	>25	ND	>250
*pbgE*	<10	12	6–12	1–2
*pbgE/*P_lac_pbgPE	ND	>25	>100	>250

^*^MICs were determined by culturing bacterial strains for 48 h with various concentrations of insect (cecropins) and non-insect CAMPs (colistin and polymyxin B). All these experiments were performed at least three times.
