# Supplementary material for: An antimicrobial peptide-resistant minor subpopulation of Photorhabdus luminescens is responsible for virulence
Source: Sci Rep. 2017 Mar 2;7:43670. doi: 10.1038/srep43670 (PMC5333078; doi:10.1038/srep43670)
Supplement: Supplementary Information [file srep43670-s1.pdf]

Supplementary Information:

An antimicrobial peptide-resistant minor subpopulation of *Photorhabdus luminescens* is responsible for virulence

Annabelle Mouammine[1*], Sylvie Pages[1,] Anne Lanois[1], Sophie Gaudriault[1], Gregory Jubelin[2], Maurine Bonabaud[3], Stéphane Cruveiller[4], Emeric Dubois[3], David Roche[4], Ludovic Legrand[5], Julien Brillard[1] and Alain Givaudan[1#]

[1] DGIMI, INRA, Univ. Montpellier, Montpellier, France

[2] INRA, UR454 Microbiologie, Saint-Genès-Champanelle, France

[3] MGX-Montpellier GenomiX, c/o IGF, Montpellier, France

[4] Laboratoire d'Analyse Bioinformatique en Génomique et Métabolisme, CEA, Genoscope & CNRS, Evry, France

[6] LIPM, INRA, CNRS, Castanet-Tolosan, France

Supplementary Legends:

Supplementary Figure 1: Only 0.5% of TT01 bacteria are resistant to polymyxin B. The proportion of resistant TT01 bacteria was assessed by counting CFUs on nutrient agar plates in the presence (100 µg/mL) and absence of polymyxin B. The proportion of resistant bacteria for each OD_{540} tested is shown in the boxes. The black line is a representative curve of the growth of the TT01 strain over time. Experiments were performed at least three times.

Supplementary Figure 2: No insect death and septicemia observed after the injection of the *phoP* or *pbgE* mutants. Bacterial growth and insect larval mortality following the injection of the *phoP* or *pbgE* mutant strains into *Spodoptera littoralis.* We injected about 10^4

bacteria into each larva at time zero. The histogram shows the mean numbers of CFU of *phoP* (in green) or *pbgE* (in blue) recovered from four larvae per time point, after plating on nutrient agar. The error bars indicate the standard error of the mean. The lines indicate larval mortality rates (for 20 larvae per treatment). One representative experiment from more than three independent experiments carried out is shown.

Supplementary Figure 3: The resistant subpopulation kills insects more rapidly than the wild-type strain. Survival curves over time are shown for TT01 (blue) and the resistant subpopulation preselected with polymyxin B (green). For each set of conditions, we injected 10^3 bacteria in a volume of 20 µl into each of 20 *Spodoptera littoralis* larvae. More than seven independent experiments were analyzed with R software. Statistically significant differences were detected ($p < 0.005$; Wilcoxon test).

Supplementary Table 1: Mutations identified in the polymyxin B-resistant population

Supplementary Table 2A: List of 55 genes differentially expressed between *P. luminescens* TT01 wild-type strain and its isogenic *phoP* mutant in LB culture.

Supplementary Table 2B: List of 64 genes differentially expressed between *P. luminescens* TT01 wild-type strain and its isogenic *phoP* mutant in low Mg^{2+} culture.

Supplementary Table 2C: List of the 21 genes belonging to the core PhoP regulon of *P. luminescens* TT01.

Supplementary Table 3A: List of 445 genes differentially expressed in *P. luminescens* TT01 between LB culture and LB+polymyxin B culture.

Supplementary Table 3B: List of 208 genes underexpressed in *P. luminescens* TT01 between LB culture and LB+polymyxin B culture.

Supplementary Table 3C: List of 237 genes overexpressed in *P. luminescens* TT01 between LB culture and LB+polymyxin B culture.

Supplementary Table 3D: RT-qPCR comparative analysis of 20 differentially expressed marker genes in polyB resistant clones (PolyR) cultivated in LB+polyB or in LB cultures.

Supplementary Table 4: Bacterial strains and plasmids used in this study.

Supplementary Table 5: Oligonucleotides used in this study.

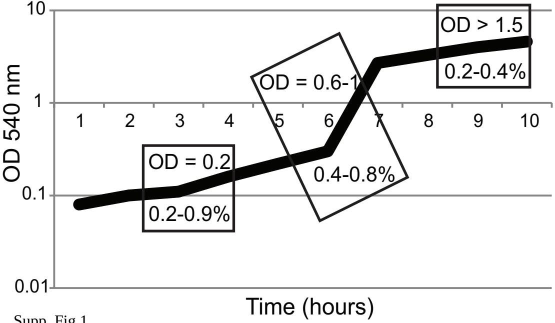

OD 540 nm
10
1
0.1
0.01
1
2
3
4
5
6
7
8
9
10
OD = 0.2
0.2-0.9%
OD = 0.6-1
0.4-0.8%
OD > 1.5
0.2-0.4%
Time (hours)

Supp. Fig.1

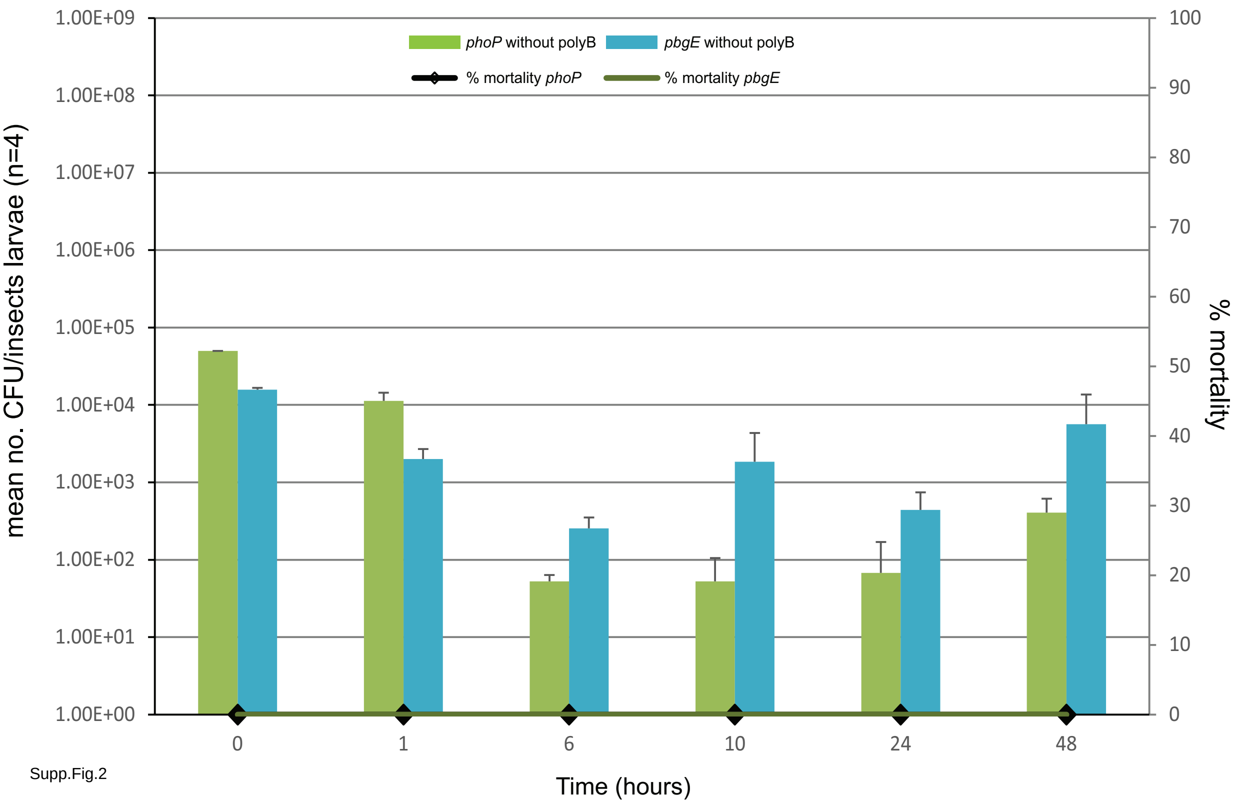

phoP without polyB
pbgE without polyB
% mortality phoP
% mortality pbgE
mean no. CFU/insects larvae (n=4)
% mortality
Time (hours)
1.00E+09
1.00E+08
1.00E+07
1.00E+06
1.00E+05
1.00E+04
1.00E+03
1.00E+02
1.00E+01
1.00E+00
100
90
80
70
60
50
40
30
20
10
0
0
1
6
10
24
48

Supp.Fig.2

Supp. Figure 3

.

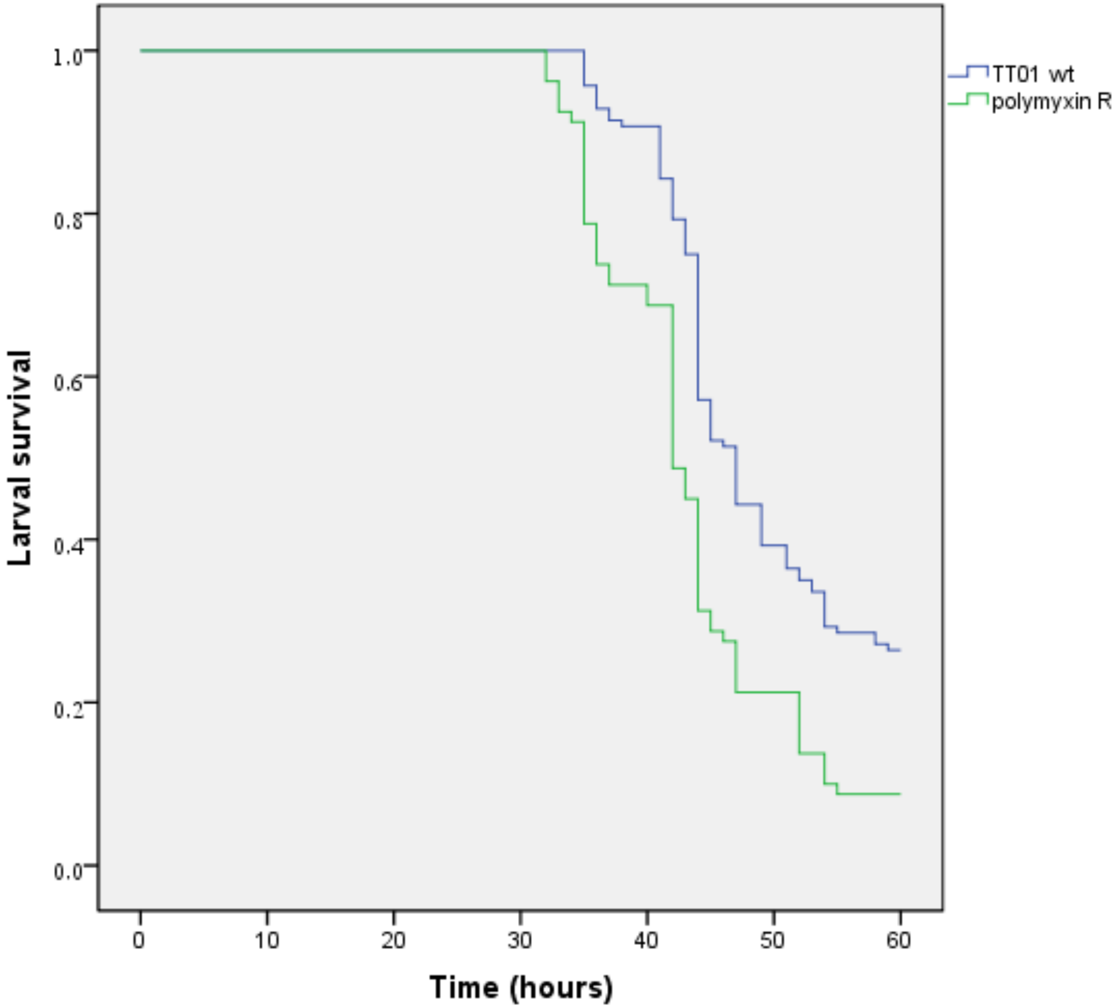

Supplementary Table 1 : Putative mutations identified in the polymyxin B-resistant population [1]

Proposed mutation	Position in reference	Sequence in reference	Alternative sequence	Inside gene	Repeated region (defined by MaGe)	Other repeat	BlastN region analysis
Deletion	22 403	GG	G	plu0024 (pts11)		duplicated phagic region	mutation not confirmed
Deletion	23 470	GT	G			duplicated phagic region	mutation not confirmed
Deletion	23 777	CT	C		yes	duplicated phagic region	mutation not confirmed
Deletion	24 262	GC	G			duplicated phagic region	mutation not confirmed
Deletion	24 448	TA	T	plu0027 (pts8)		duplicated phagic region	mutation not confirmed
Deletion	24 489	CA	C	plu0027 (pts8)		duplicated phagic region	mutation not confirmed
Deletion	24 589	GA	G	plu0027 (pts8)		duplicated phagic region	mutation not confirmed
Deletion	24 602	CA	C	plu0027 (pts8)		duplicated phagic region	mutation not confirmed
SNP	26 515	T	C	plu0030 (pts5)	yes	duplicated phagic region	mutation not confirmed
Deletion	28 253	TC	T			duplicated phagic region	mutation not confirmed
Deletion	994 863	TTAAT	T				mutation not confirmed
Deletion	1 065 824	ACTGTaaaactgtaaaa	A		yes		mutation not confirmed
Insertion	1 324 642	A	AA	plu1145	yes		mutation not confirmed
Insertion	1 714 443	T	TT				mutation not confirmed
Deletion	3 525 319	CA	C	plu3025			mutation not confirmed
SNP	4 207 440	AA	TT		yes		mutation not confirmed
Deletion	4 207 442	AACT	A		yes		mutation not confirmed
SNP	4 207 446	ATC	CAG		yes		mutation not confirmed
Deletion	4 779 633	Tgaagtaaatagttatt	T		yes		mutation not confirmed
**Insertion**	**5 531 259**	**C**	**Caaataagt**		yes		not confirmed but a 12Nt deletion is observed in the polymyxin B resistant population

[1] The 20 mutations were identified in the polymyxin B-resistant population compared to the control population following the procedure described in Methods section. Their position refers to that in the reference *P. luminescens* TT01 NC_005126 genome. The majority (n=17) of these 20 mutations was found in duplicated regions or in regions with a high number of repeats. We performed a BlastN comparison of the 0.4-1Kb regions surrounding each of these 20 putative mutations identified in the polymyxin B-resistant population against the control population. It revealed 100% identity for 19 of them between the two populations. Therefore, only one mutation at position 5 531 259 (indicated in bold) was identified: a 12-nucleotide deletion in RPT23004543, a 516-bp repeated sequence, itself located in RPTR964997, a broad region with a large number of repeats (n=435). It therefore remains unclear whether this deletion is a true mutation in the polymyxin B-resistant population or whether it is an artifact due to an incorrect interpretation of the sequence. However, given its location in an intergenic region 395 bp downstream from the stop codon of plu4751 (a transposase-encoding gene) and 617 bp upstream from the start codon of plu4750 (encoding a putative T3SS effector), this hypothetical mutation presumably has no function related to the polymyxin-B resistance of *P. luminescens* TT01.

**Supplementary Table 2A : ** List of 55 genes differentially expressed between *P. luminescens* TT01 wild-type strain and its isogenic *phoP* mutant in LB culture (logarithmic phase, FDR≤0.005, abs(log2FC)≥1)

# # indicates the genes belonging to the core PhoP regulon.

* ratio phoP/WT

Label	Type	Name	Product	Begin	End	Length	Frame	normalized average read count	log2 fold change*	adjusted p-value (FDR)
plu0104	CDS	-	Conserved hypothetical secreted protein	101566	102060	495	1	241	-1.93	1.08e-6
plu0480	CDS	-	Conserved hypothetical protein with Galactose-binding like domain (IPR008979 domain)	520584	521060	477	-2	2824	-1.30	1.70e-6
**plu0691##**	**CDS**	**cutF**	**Copper homeostasis protein CutF precursor**	**794390**	**795061**	**672**	**2**	**1335**	**-1.98**	**4.34e-17**
plu0769	CDS	mrfA	Major fimbrial subunit polypeptide, MrfA	888552	889091	540	3	1488	2.28	6.92e-23
plu0770	CDS	mrfB	Fimbrial pilin protein precursor, MrfB	889175	889723	549	2	55	2.22	3.92e-5
plu0771	CDS	mrfC	Outer membrane usher protein MrfC	889743	892298	2556	3	257	2.02	3.30e-8
plu0858	CDS	-	Conserved hypothetical protein; putative transmembrane protein	987602	988066	465	-3	29	-3.28	8.64e-6
plu1334	CDS	-	Similar to unknown protein VC1449 of Vibrio cholerae	1552827	1553192	366	3	132	1.79	2.40e-4
plu1335	CDS	rtxC	Cytolysin-activating lysine-acyltransferase RtxC	1553214	1553675	462	3	377	1.62	9.96e-6
plu1575	CDS	-	Similar to crystalline inclusion protein type II (CipA-like protein)	1882485	1882823	339	-2	4463	-1.10	2.31e-5
**plu1579##**	**CDS**	**-**	**putative bacitracin transport permease YbjG of Escherichia coli**	**1888106**	**1888714**	**609**	**-3**	**234**	**-2.07**	**1.61e-9**
plu1889	CDS	-	conserved hypothetical protein	2252258	2252443	186	-3	422	-1.22	2.52e-3
plu1954	CDS	fliC	Flagellin	2319961	2321028	1068	1	597	1.61	3.46e-7
plu2143	CDS	-	hypothetical protein	2525733	2526353	621	-2	1933	-1.04	1.02e-3
**plu2480##**	**CDS**	**ail1**	**Ail protein precursor of Yersinia**	**2920701**	**2921228**	**528**	**-2**	**924**	**-2.37**	**2.62e-21**
plu2507	CDS	ygbJ	Highly similar to hypothetical oxidoreductase YgbJ of Escherichia coli	2951054	2951959	906	2	639	1.13	2.61e-3
pluCDS3053012R	CDS	-	hypothetical protein	3053012	3053125	114	-3	145	-1.49	2.07e-3
plu2655	CDS	pbgE2	4-amino-4-deoxy-L-arabinose-phosphoundecaprenol flippase, PbgE2	3108050	3108391	342	-3	79	-1.56	4.45e-3
plu2656	CDS	pbgE1	4-amino-4-deoxy-L-arabinose transferase, PbgE1 protein	3108388	3110049	1662	-1	384	-1.50	2.23e-6
**plu2657##**	**CDS**	**pbgP4**	**Conserved hypothetical protein PbgP4**	**3110057**	**3110950**	**894**	**-3**	**568**	**-1.88**	**2.07e-11**
**plu2658##**	**CDS**	**pbgP3**	**UDP-L-Ara4N formyltransferase ; UDP-GlcA C-4'-decarboxylase PbgP3**	**3110950**	**3112932**	**1983**	**-1**	**1364**	**-2.20**	**8.52e-21**
**plu2659##**	**CDS**	**pbgP2**	**undecaprenyl phosphate-L-Ara4FN transferase PbgP2**	**3112933**	**3113910**	**978**	**-1**	**339**	**-2.36**	**8.77e-14**
**plu2660##**	**CDS**	**pbgP1**	**UDP-4-amino-4-deoxy-L-arabinose--oxoglutarate aminotransferase PbgP1**	**3113911**	**3115056**	**1146**	**-1**	**435**	**-2.70**	**4.89e-20**
plu2784	CDS	pagP	LipidA acyltransferase PagP	3305063	3305686	624	2	54	-5.04	1.21e-18
plu2807	CDS	phoP	Two-component response regulator PhoP	3332475	3333143	669	3	122	-1.60	2.92e-4
plu2959	CDS	-	Some similarities with bacteriophage protein	3462838	3463425	588	-1	642	-1.17	3.25e-3
plu2962	CDS	-	putative bacteriophage tail fiber protein	3466665	3468149	1485	-2	290	-1.55	2.36e-4
plu3259	CDS	-	Truncated gene. Some similarities with unknown protein	3860162	3860554	393	-3	20	-2.66	5.13e-5
plu3260	CDS	-	putative Type VI secretion protein, VC_A0114 (IPR010263)	3860551	3861900	1350	-1	114	-2.32	8.44e-9
plu3261	CDS	-	putative type VI secretion protein, EvpB/VC_A0108, tail sheath (domain IPR010269)	3861916	3863442	1527	-1	162	-2.36	5.75e-10
plu3262	CDS	-	putative Type VI secretion system, VipA, VC_A0107 or Hcp2 (domain IPR008312)	3863474	3863971	498	-3	196	-2.31	1.50e-10
plu3288	CDS	treB	PTS system, trehalose-specific IIBC component (EIIBC-TRE) (trehalose-permease IIBC component) (phosphotransferase enzyme II, BC component) (EII-TRE)	3903006	3904427	1422	-2	436	1.20	1.98e-3
**plu3736##**	**CDS**	**mgtE**	**Magnesium transport protein MgtE**	**4405167**	**4406237**	**1071**	**-2**	**1418**	**-3.98**	**1.13e-56**
plu3749	CDS	-	putative phage lysis protein S (holin)	4421407	4421733	327	-1	500	-1.20	2.07e-3
**plu3988##**	**CDS**	**-**	**conserved hypothetical protein**	**4674261**	**4675196**	**936**	**3**	**542**	**-6.57**	**2.64e-72**
**plu4210##**	**CDS**	**-**	**Conserved hypothetical protein with SANT/Myb DNA-binding domain (IPR001005)**	**4923248**	**4925854**	**2607**	**-3**	**216**	**-1.94**	**9.93e-8**
**plu4211##**	**CDS**	**-**	**Putative Type VI secretion system effector, Hcp (IPR008514)**	**4925955**	**4926446**	**492**	**-2**	**351**	**-3.05**	**1.89e-22**
**plu4212##**	**CDS**	**-**	**Hypothetical protein**	**4926419**	**4926589**	**171**	**-3**	**58**	**-3.49**	**5.83e-12**
plu4214	CDS	-	Putative type VI secretion system IcmF, C-terminal (domains IPR010623 and IPR009612)	4927191	4930550	3360	-2	168	-2.46	1.77e-11
plu4215	CDS	-	Conserved hypothetical protein. Putative membrane protein.	4930543	4931718	1176	-1	80	-2.60	2.42e-9
plu4216	CDS	-	Conserved hypothetical protein	4931721	4931996	276	-2	46	-2.72	9.79e-8
plu4217	CDS	-	Conserved hypothetical protein. Putative membrane protein	4932024	4932692	669	-2	88	-3.03	1.65e-11
plu4218	CDS	-	Conserved hypothetical protein. Putative membrane protein.	4932878	4933867	990	-3	255	-1.49	9.38e-5
plu4219	CDS	-	Conserved hypothetical protein. Putative membrane protein.	4933945	4934955	1011	-1	339	-1.25	4.69e-4
**plu4220##**	**CDS**	**-**	**Conserved hypothetical protein with SANT/Myb DNA-binding domain (IPR001005)**	**4934952**	**4937552**	**2601**	**-2**	**531**	**-3.14**	**8.62e-29**
**plu4221##**	**CDS**	**-**	**Conserved hypothetical protein**	**4937552**	**4938592**	**1041**	**-3**	**144**	**-4.69**	**1.26e-27**
**plu4222##**	**CDS**	**-**	**Putative Type VI secretion system, RhsGE-associated Vgr protein (domain IPR006533)**	**4938603**	**4940981**	**2379**	**-2**	**310**	**-4.93**	**3.17e-43**
**plu4223##**	**CDS**	**-**	**Putative type VI secretion ATPase, ClpV1 family**	**4940978**	**4943659**	**2682**	**-3**	**657**	**-4.73**	**4.59e-57**
**plu4224##**	**CDS**	**-**	**Putative Outer membrane protein, OmpA/MotB, C-terminal (domain IPR006665)**	**4943908**	**4945584**	**1677**	**-1**	**576**	**-5.36**	**2.02e-62**
**plu4225##**	**CDS**	**-**	**conserved hypothetical protein. Putative membrane protein**	**4945601**	**4946245**	**645**	**-3**	**380**	**-5.98**	**4.15e-57**
**plu4226##**	**CDS**	**-**	**Putative Type VI secretion protein, VC_A0114 (domain IPR010263)**	**4946288**	**4947649**	**1362**	**-3**	**689**	**-5.82**	**1.58e-73**
**plu4227##**	**CDS**	**-**	**Putative Type VI secretion protein, EvpB/VC_A0108, tail sheath (domain IPR010269)**	**4947666**	**4949198**	**1533**	**-2**	**1092**	**-6.26**	**2.72e-94**
**plu4291##**	**CDS**	**-**	**conserved hypothetical protein**	**5018093**	**5019289**	**1197**	**2**	**160**	**-3.27**	**8.02e-17**
plu4558	CDS	ecfJ	Function unknown; rpoE regulon; periplasmic protein; expressed protein (by similarity)	5321428	5321982	555	1	1696	-1.09	2.92e-4
plu4748	CDS	-	Conserved hypothetical protein; putative secreted protein	5528419	5529000	582	1	1954	-1.10	4.69e-4

**Supplementary Table 2B : ** List of 64 genes differentially expressed between *P. luminescens* TT01 wild-type strain and its isogenic *phoP* mutant in low Mg^{2+} culture (logarithmic phase, FDR≤0.005, abs(log2FC)≥1)
indicates the genes belonging to the core PhoP regulon
* ratio phoP/WT

Label	Type	Name	Product	Begin	End	Length	Frame	normalized average read count	log2 fold change*	adjusted pvalue (FDR)
plu0158	CDS	amt	L-arginine:lysine amidinotransferase	163665	164765	1101	3	836	-1.64	2.49e-4
plu0159	CDS	-	conserved hypothetical protein	164775	165785	1011	3	166	-1.56	3.30e-3
plu0261	CDS	madA	fimbrial protein MadA	277866	278471	606	3	145	2.09	5.06e-5
plu0655	CDS	prtA	Serralysin-type metalloprotease, PrtA	752825	754267	1443	2	8176	-1.17	3.69e-3
plu0656	CDS	inh	Serralysin-type metalloprotease PrtA inhibitor, Inh	754322	754726	405	2	1136	-1.56	5.74e-4
plu0657	CDS	prtB	ATP-binding protein PrtB	754786	756516	1731	1	1779	-1.61	2.15e-4
plu0658	CDS	prtC	Membrane Fusion Protein PrtC	756583	757911	1329	1	964	-1.59	4.01e-4
plu0659	CDS	prtD	Outer membrane protein PrtD	757911	759287	1377	3	734	-1.41	4.02e-3
**plu0691##**	**CDS**	**cutF**	**Copper homeostasis protein CutF precursor**	**794390**	**795061**	**672**	**2**	**562**	**-2.06**	**2.01e-6**
plu1235	CDS	-	putative sigma-70 factor, ECF subfamily	1427552	1428052	501	-3	45	2.52	6.33e-4
plu1369	CDS	-	Putative metal-dependent beta-lactamase	1641988	1642788	801	1	66	-2.04	1.14e-3
plu1574	CDS	-	Conserved hypothetical protein	1881043	1881897	855	-1	1159	-1.60	4.01e-4
**plu1579##**	**CDS**	**-**	**putative bacitracin transport permease YbjG of Escherichia coli**	**1888106**	**1888714**	**609**	**-3**	**232**	**-2.68**	**1.50e-9**
plu1843	CDS	mgtC	-	2190810	2191520	711	3	320	1.83	1.09e-4
plu1915	CDS	flgB	Flagellar basal-body rod protein FlgB (Putative proximal rod protein)	2281971	2282384	414	3	47	2.43	1.22e-3
plu1969	CDS	dsdX	DsdX permease	2341397	2342734	1338	2	1200	2.74	4.14e-11
plu1970	CDS	dsdA	D-serine dehydratase DsdA	2343419	2344750	1332	2	1052	1.60	5.74e-4
plu2227	CDS	ynfK	Putative dethiobiotin synthetase YnfK of Escherichia coli	2620291	2620998	708	1	77	-1.82	2.92e-3
plu2256	CDS	-	conserved hypothetical protein	2649009	2649305	297	3	787	-1.54	6.33e-4
plu2336	CDS	-	-	2751654	2753417	1764	-2	282	-1.76	7.34e-5
**plu2480##**	**CDS**	**ail1**	**Ail protein precursor of Yersinia**	**2920701**	**2921228**	**528**	**-2**	**1106**	**-1.56**	**6.33e-4**
plu2631	CDS	-	-	3081215	3081412	198	2	588	1.79	1.05e-4
**plu2657##**	**CDS**	**pbgP4**	**Conserved hypothetical protein PbgP4**	**3110057**	**3110950**	**894**	**-3**	**279**	**-1.74**	**5.14e-4**
**plu2658##**	**CDS**	**pbgP3**	**UDP-L-Ara4N formyltransferase ; UDP-GlcA C-4'-decarboxylase PbgP3**	**3110950**	**3112932**	**1983**	**-1**	**566**	**-2.16**	**1.41e-6**
**plu2659##**	**CDS**	**pbgP2**	**undecaprenyl phosphate-L-Ara4FN transferase PbgP2**	**3112933**	**3113910**	**978**	**-1**	**171**	**-2.59**	**1.30e-7**
**plu2660##**	**CDS**	**pbgP1**	**UDP-4-amino-4-deoxy-L-arabinose--oxoglutarate aminotransferase PbgP1**	**3113911**	**3115056**	**1146**	**-1**	**249**	**-2.18**	**4.17e-6**
plu2999	CDS	cbiA	Cobyrinic acid A,C-diamide synthase	3499135	3500517	1383	-1	84	-2.27	3.61e-5
plu3067	CDS	-	conserved hypothetical protein	3575125	3575667	543	-1	359	-1.65	3.20e-4
plu3075	CDS	phaxA2	PhaxA2, similar to binary toxin XaxA of Xenorhabdus nematophila	3585640	3586866	1227	1	406	-1.96	3.75e-6
plu3076	CDS	phaxB2	PhaxB2, similar to binary toxin XaxB of Xenorhabdus nematophila	3586913	3587974	1062	2	393	-1.84	1.96e-5
plu3129	CDS	-	conserved hypothetical protein	3677817	3678092	276	3	124	-1.58	4.40e-3
plu3134	CDS	-	putative 4-hydroxybenzoate transporter	3692924	3694246	1323	-3	182	-1.89	7.34e-5
plu3512	CDS	-	Some similarities with energy transducer TonB. Putative secreted protein	4104111	4104878	768	-2	396	1.44	4.40e-3
plu3513	CDS	-	Putative TonB-receptor	4104961	4108005	3045	-1	1659	1.56	5.74e-4
plu3514	CDS	-	conserved hypothetical protein	4108262	4109761	1500	-3	881	1.85	5.08e-5
plu3595	CDS	-	-	4242432	4242791	360	-2	60	2.64	1.82e-4
**plu3736##**	**CDS**	**mgtE**	**Magnesium transport protein MgtE**	**4405167**	**4406237**	**1071**	**-2**	**1920**	**-2.20**	**1.12e-7**
plu3795	CDS	-	conserved hypothetical protein	4459644	4460093	450	-2	1079	-2.00	7.12e-6
plu3923	CDS	-	-	4601127	4601714	588	-2	300	-1.53	2.70e-3
**plu3988##**	**CDS**	**-**	**conserved hypothetical protein**	**4674261**	**4675196**	**936**	**3**	**128**	**-7.23**	**3.73e-27**
plu4084	CDS	-	putative transport protein and multidrug resistance protein like bicyclomycin resistance protein	4770938	4772125	1188	-3	93	2.49	4.24e-5
**plu4210##**	**CDS**	**-**	**Conserved hypothetical protein with SANT/Myb DNA-binding domain (IPR001005)**	**4923248**	**4925854**	**2607**	**-3**	**228**	**-1.98**	**4.24e-5**
**plu4211##**	**CDS**	**-**	**Putative Type VI secretion system effector, Hcp (IPR008514)**	**4925955**	**4926446**	**492**	**-2**	**280**	**-3.43**	**5.51e-15**
**plu4212##**	**CDS**	**-**	**Hypothetical protein**	**4926419**	**4926589**	**171**	**-3**	**40**	**-3.83**	**2.57e-7**
**plu4220##**	**CDS**	**-**	**Conserved hypothetical protein with SANT/Myb DNA-binding domain (IPR001005)**	**4934952**	**4937552**	**2601**	**-2**	**176**	**-1.97**	**1.68e-4**
**plu4221##**	**CDS**	**-**	**Conserved hypothetical protein**	**4937552**	**4938592**	**1041**	**-3**	**44**	**-2.66**	**4.01e-4**
**plu4222##**	**CDS**	**-**	**Putative Type VI secretion system, RhsGE-associated Vgr protein (domain IPR006533)**	**4938603**	**4940981**	**2379**	**-2**	**84**	**-2.80**	**2.45e-6**
**plu4223##**	**CDS**	**-**	**Putative type VI secretion ATPase, ClpV1 family**	**4940978**	**4943659**	**2682**	**-3**	**147**	**-3.19**	**2.14e-10**
**plu4224##**	**CDS**	**-**	**Putative Outer membrane protein, OmpA/MotB, C-terminal (domain IPR006665)**	**4943908**	**4945584**	**1677**	**-1**	**231**	**-4.80**	**1.10e-23**
**plu4225##**	**CDS**	**-**	**conserved hypothetical protein. Putative membrane protein**	**4945601**	**4946245**	**645**	**-3**	**144**	**-4.95**	**2.76e-20**
**plu4226##**	**CDS**	**-**	**Putative Type VI secretion protein, VC_A0114 (domain IPR010263)**	**4946288**	**4947649**	**1362**	**-3**	**244**	**-4.21**	**5.04e-20**
**plu4227##**	**CDS**	**-**	**Putative Type VI secretion protein, EvpB/VC_A0108, tail sheath (domain IPR010269)**	**4947666**	**4949198**	**1533**	**-2**	**292**	**-4.59**	**1.10e-23**
plu4253	CDS	yjjZ	-	4975792	4976004	213	-1	315	1.58	1.50e-3
**plu4291##**	**CDS**	**-**	**conserved hypothetical protein**	**5018093**	**5019289**	**1197**	**2**	**133**	**-2.80**	**1.05e-8**
plu4446	CDS	fecA	Iron(III) dicitrate outer membrane transporter precursor protein FecA	5194611	5196971	2361	3	97	2.09	4.01e-4
plu4565	CDS	pcfD	Carbamoyl transferase, NodU family, involved in the induction of Photorhabdus cell clumping	5329532	5331229	1698	-3	591	-2.01	1.80e-6
plu4566	CDS	pcfC	Glycine amidinotransferase, involved in the induction of Photorhabdus cell clumping	5331253	5332056	804	-1	186	-1.82	1.21e-4
plu4567	CDS	pcfB	Argininosuccinate synthase, involved in the induction of Photorhabdus cell clumping	5332053	5333243	1191	-2	155	-1.51	4.02e-3
plu4568	CDS	pcfA	cysteine synthase, O-acetylserine sulfhydrolase subunit, involved in the induction of Photorhabdus cell clumping	5333233	5334261	1029	-1	155	-1.82	2.53e-4
plu4627	CDS	fhuF	Ferric iron reductase protein FhuF	5393763	5394542	780	3	236	1.77	2.53e-4
plu4797	CDS	wblB	Myo-inositol 2-dehydrogenase (WblB protein)	5571065	5572114	1050	2	572	-1.36	3.94e-3
plu4809	CDS	wblM	WblM protein	5583742	5585616	1875	1	258	-1.42	4.40e-3
plu4813	CDS	wblQ	WblQ protein	5589089	5590216	1128	2	90	1.98	1.09e-3
plu4814	CDS	wzxB	WzxB protein	5590261	5591466	1206	1	104	2.03	5.74e-4

**Supplementary Table 2C : ** List of the 21 genes belonging to the core PhoP regulon of *P. luminescens* TT01
indicates the genes belonging to the core PhoP regulon
* ratio phoP/WT

Label	Type	Name	Product	log2 fold change* LB culture RNA-Seq	log2 fold change* LB culture RT-qPCR	log2 fold change* Low Mg2+ RNA-Seq	log2 fold change* Low Mg2+ RT-qPCR
**plu0691##**	**CDS**	**cutF**	**Copper homeostasis protein CutF precursor**	**-1.98**	-1.86	**-2.06**	-2.09
**plu1579##**	**CDS**	**-**	**putative bacitracin transport permease YbjG of Escherichia coli**	**-2.07**		**-2.68**	
**plu2480##**	**CDS**	**ail1**	**Ail protein precursor of Yersinia**	**-2.37**	-2.26	**-1.56**	-2.32
**plu2657##**	**CDS**	**pbgP4**	**Conserved hypothetical protein PbgP4**	**-1.88**		**-1.74**	
**plu2658##**	**CDS**	**pbgP3**	**UDP-L-Ara4N formyltransferase ; UDP-GlcA C-4'-decarboxylase PbgP3**	**-2.20**		**-2.16**	
**plu2659##**	**CDS**	**pbgP2**	**undecaprenyl phosphate-L-Ara4FN transferase PbgP2**	**-2.36**		**-2.59**	
**plu2660##**	**CDS**	**pbgP1**	**UDP-4-amino-4-deoxy-L-arabinose--oxoglutarate aminotransferase PbgP1**	**-2.70**	-4.21	**-2.18**	-3.68
**plu3736##**	**CDS**	**mgtE**	**Magnesium transport protein MgtE**	**-3.98**	-4.06	**-2.20**	-4.76
**plu3988##**	**CDS**	**-**	**conserved hypothetical protein**	**-6.57**	-7.16	**-7.23**	-7.16
**plu4210##**	**CDS**	**-**	**Conserved hypothetical protein with SANT/Myb DNA-binding domain (IPR001005)**	**-1.94**		**-1.98**	
**plu4211##**	**CDS**	**-**	**Putative Type VI secretion system effector, Hcp (IPR008514)**	**-3.05**	-2.82	**-3.43**	-3.7
**plu4212##**	**CDS**	**-**	**Hypothetical protein**	**-3.49**		**-3.83**	
**plu4220##**	**CDS**	**-**	**Conserved hypothetical protein with SANT/Myb DNA-binding domain (IPR001005)**	**-3.14**		**-1.97**	
**plu4221##**	**CDS**	**-**	**Conserved hypothetical protein**	**-4.69**		**-2.66**	
**plu4222##**	**CDS**	**-**	**Putative Type VI secretion system, RhsGE-associated Vgr protein (domain IPR006533)**	**-4.93**		**-2.80**	
**plu4223##**	**CDS**	**-**	**Putative type VI secretion ATPase, ClpV1 family**	**-4.73**		**-3.19**	
**plu4224##**	**CDS**	**-**	**Putative Outer membrane protein, OmpA/MotB, C-terminal (domain IPR006665)**	**-5.36**		**-4.80**	
**plu4225##**	**CDS**	**-**	**conserved hypothetical protein. Putative membrane protein**	**-5.98**		**-4.95**	
**plu4226##**	**CDS**	**-**	**Putative Type VI secretion protein, VC_A0114 (domain IPR010263)**	**-5.82**		**-4.21**	
**plu4227##**	**CDS**	**-**	**Putative Type VI secretion protein, EvpB/VC_A0108, tail sheath (domain IPR010269)**	**-6.26**	-6.96	**-4.59**	-6.06
**plu4291##**	**CDS**	**-**	**conserved hypothetical protein**	**-3.27**		**-2.80**	

Supplementary Table 3A: List of 445 genes differentially expressed in *P. luminescens* TT01 between LB culture and LB+polyB culture (logarithmic phase, FDR≤0.005, abs(log2FC)≥1)
# indicates the genes belonging to the PhoP regulon in LB broth.
# # indicates the genes belonging to the core PhoP regulon.
* ratio LB+polyB/LB
Colors highlight some functional categories:
components of secretion system (33 genes)
insecticidal toxins (9 genes)
transporter (25 genes)
extracellular enzymes

Label	Type	Name	Product	COG_ID	COG_classID	COGClassDescription	Begin	End	normalized average read count	log2 fold change*	adjusted p-value (FDR)
plu0008	CDS	ogrK	Ogr protein; transcriptional activator for bacteriophage P2 late genes			Unclassified	10251	10472	45	-1.99	8.01e-4
plu0010	CDS	pts25	Phage tail protein	COG3499	X	Mobilome: prophages, transposons	11631	12095	71	-1.83	5.66e-4
plu0011	CDS	pts24	Phage tail protein	COG5283	X	Mobilome: prophages, transposons	12098	14536	382	-3.55	7.26e-26
plu0012	CDS	pts23	Phage tail protein			Unclassified	14517	14636	75	-5.20	6.86e-19
plu0013	CDS	pts22	Phage tail protein			Unclassified	14651	14968	404	-5.40	4.19e-49
plu0014	CDS	pts21	Major phage tail tube protein	COG3498	X	Mobilome: prophages, transposons	14995	15510	670	-5.12	1.47e-55
plu0015	CDS	pts20	Major phage tail sheath protein	COG3497	X	Mobilome: prophages, transposons	15521	16693	1243	-5.87	1.78e-81
plu0019	CDS	pts16	Tail fiber assembly protein	COG2110	J	Translation, ribosomal structure and biogenesis	18438	19034	127	-1.79	2.08e-5
plu0020	CDS	pts15	Alternative tail fiber protein of bacteriophage-related plasmid and bacteriophage	COG4675	S	Function unknown	19034	19522	89	-1.68	1.19e-3
plu0021	CDS	pts14	Tail fiber protein of bacteriophage	COG5301	X	Mobilome: prophages, transposons	19522	20649	145	-2.10	1.45e-6
plu0022	CDS	pts13	Phage tail protein	COG4385	X	Mobilome: prophages, transposons	20646	21260	82	-2.31	1.27e-6
plu0023	CDS	pts12	Phage baseplate assembly protein	COG3948	X	Mobilome: prophages, transposons	21253	22251	132	-2.59	4.16e-10
plu0024	CDS	pts11	Phage baseplate assembly protein	COG3628	X	Mobilome: prophages, transposons	22256	22597	54	-2.98	8.39e-9
plu0025	CDS	pts10	Phage-related baseplate assembly protein	COG4540	X	Mobilome: prophages, transposons	22597	23439	98	-3.47	6.24e-14
plu0026	CDS	pts9	Phage tail protein	COG5004	X	Mobilome: prophages, transposons	23936	24139	35	-3.96	1.48e-10
plu0052	CDS	asnA	Aspartate--ammonia ligase (Asparagine synthetase A)	COG2502	E	Amino acid transport and metabolism	46030	47022	637	1.92	1.04e-9
plu0072	CDS	yjcG	-	COG4147	C	Energy production and conversion	69280	70929	370	1.41	3.72e-5
plu0073	CDS	yjcH	-	COG3162	S	Function unknown	70926	71237	34	2.02	6.47e-4
plu0074	CDS	acs	acetyl-coenzyme A synthetase (acetate--COA ligase) (acyl-activating enzyme)	COG0365	I	Lipid transport and metabolism	71302	73257	1315	1.92	3.29e-14
plu0082	CDS	-	conserved hypothetical protein	COG5572	S	Function unknown	78376	78669	174	1.22	4.44e-3
plu0100	CDS	pckA	phosphoenolpyruvate carboxykinase [ATP]	COG1866	C	Energy production and conversion	96415	98034	1188	1.13	1.24e-5
**plu0104#**	**CDS**	**-**	**Conserved hypothetical secreted protein**	**COG3193**	**S**	**Function unknown**	**101566**	**102060**	**166**	**-6.83**	**1.21e-36**
plu0112	CDS	cynS	cyanate lyase (cyanate hydrolase) (cyanase)	COG1513	P	Inorganic ion transport and metabolism	115374	115844	157	1.23	4.63e-3
plu0121	CDS	uspA	universal stress protein A	COG0589	T	Signal transduction mechanisms	126961	127392	1033	1.11	3.98e-5
plu0157	CDS	cipB	Crystalline inclusion protein CipB			Unclassified	162599	162901	68	-2.21	4.00e-4
plu0158	CDS	amt	L-arginine:lysine amidinotransferase	COG1834	E	Amino acid transport and metabolism	163665	164765	79	-2.80	1.50e-8
plu0162	CDS	-	conserved hypothetical protein	COG1680	V	Defense mechanisms	168154	169746	218	-1.69	3.41e-5
plu0163	CDS	-	conserved hypothetical protein	COG1680	V	Defense mechanisms	169909	170412	8.83	-3.15	1.99e-3
plu0165	CDS	-	conserved hypothetical protein	COG1680	V	Defense mechanisms	172935	174524	92	-2.11	2.21e-5
plu0182	CDS	cpmA	CpmA protein involved in carbapenem biosynthesis	COG0367	E	Amino acid transport and metabolism	192436	193941	1786	-1.30	8.04e-7
plu0183	CDS	cpmB	CpmB protein involved in carbapenem biosynthesis	COG1024	I	Lipid transport and metabolism	193944	194693	1082	-1.09	5.50e-5
plu0184	CDS	cpmC	CpmC protein involved in carbapenem biosynthesis	COG2175	Q	Secondary metabolites biosynthesis, transport and catabolism	194707	195528	1562	-1.04	5.23e-5
plu0208	CDS	feoB	ferrous iron transport protein B	COG0370	P	Inorganic ion transport and metabolism	211862	214174	703	1.36	1.84e-5
plu0209	CDS	feoA	ferrous iron transport protein A	COG1918	P	Inorganic ion transport and metabolism	214227	214460	195	1.56	1.63e-4
plu0221	CDS	-	putative amino acid permease component of ABC transporter	COG0765	E	Amino acid transport and metabolism	227701	228444	551	-1.16	9.56e-4
plu0224	CDS	-	conserved hypothetical protein			Unclassified	229778	230323	189	2.83	4.45e-14
plu0225	CDS	-	Putative TpsA-related protein	COG3210	U	Intracellular trafficking, secretion, and vesicular transport	230329	235488	737	3.62	7.75e-35
plu0226	CDS	-	TpsB protein	COG2831	U	Intracellular trafficking, secretion, and vesicular transport	235518	237197	176	4.53	3.48e-25
plu0228	CDS	-	conserved hypothetical protein	COG1807	M	Cell wall/membrane/envelope biogenesis	237589	239052	472	1.16	6.75e-4
plu0250	CDS	-	putative TRP-1 protein encoded by the toxA gene of Burkholderia glumae, methyltransferase involved in toxoflavin biosynthesis	COG4976	R	General function prediction only	267752	268495	64	-2.10	1.32e-4
plu0300	CDS	dppA	Periplasmic dipeptide transport protein precursor DppA	COG0747	E	Amino acid transport and metabolism	317950	319557	852	2.15	7.06e-16
plu0301	CDS	dppB	Dipeptide transport system permease protein DppB	COG0601	EP	Amino acid transport and metabolism, Inorganic ion transport and metabolism	319676	320695	249	1.99	1.21e-8
plu0302	CDS	dppC	Dipeptide transport system permease protein DppC	COG1173	EP	Amino acid transport and metabolism, Inorganic ion transport and metabolism	320706	321605	345	1.80	2.56e-8
plu0303	CDS	dppD	Dipeptide transport ATP-binding protein dppD	COG0444	EP	Amino acid transport and metabolism, Inorganic ion transport and metabolism	321618	322598	269	2.00	9.47e-9
plu0304	CDS	dppF	Dipeptide transport ATP-binding protein dppF	COG4608	E	Amino acid transport and metabolism	322595	323614	414	1.33	7.85e-5
plu0315	CDS	-	conserved hypothetical protein			Unclassified	333297	333845	67	-1.64	2.38e-3
plu0318	CDS	-	Putative AidA protein			Unclassified	341028	341618	145	-1.92	5.26e-5
plu0368	CDS	-	Putative Type VI secretion, VasB, ImpH, VC_A0111 (IPR010732 domain)	COG3520	U	Intracellular trafficking, secretion, and vesicular transport	395492	396472	244	-1.42	4.12e-5
plu0369	CDS	-	Putative type VI secretion system, VCA0110 (domain IPR010272)	COG3519	U	Intracellular trafficking, secretion, and vesicular transport	396508	398355	679	-1.25	8.14e-6
plu0370	CDS	-	Conserved hypothetical protein with basic-leucine zipper domain (IPR004827)	COG3518	U	Intracellular trafficking, secretion, and vesicular transport	398357	398797	248	-1.60	7.64e-6
plu0371	CDS	-	Putative type VI secretion protein, EvpB/VC_A0108, tail sheath (domain IPR010269)	COG3517	U	Intracellular trafficking, secretion, and vesicular transport	398804	400282	1379	-1.58	4.37e-10
plu0372	CDS	-	Putative Type VI secretion system, VipA, VC_A0107 or Hcp2 (domain IPR008312)	COG3516	U	Intracellular trafficking, secretion, and vesicular transport	400306	400803	679	-1.88	1.85e-11
plu0373	CDS	-	Putative Type VI secretion system effector, Hcp (IPR008514)	COG3157	U	Intracellular trafficking, secretion, and vesicular transport	401704	402222	5526	-2.01	1.25e-17
plu0418	CDS	-	Major structural subunit MrpA of Proteus mirabilis	COG3539	N	Cell motility	447141	447707	210	1.34	1.66e-4
plu0441	CDS	-	conserved hypothetical protein	COG4683	S	Function unknown	472833	473132	169	1.33	3.15e-3
**plu0480#**	**CDS**	**-**	**Conserved hypothetical protein with Galactose-binding like domain (IPR008979 domain)**	**COG2273**	**G**	**Carbohydrate transport and metabolism**	**520584**	**521060**	**1908**	**-3.34**	**7.36e-39**
plu0515	CDS	tcaC	B component of insecticidal toxin complex, protein TcaC	COG3209	R	General function prediction only	567083	571540	381	1.37	2.78e-5
plu0525	CDS	-	Similar to probable acetyltransferase	COG0456	J	Translation, ribosomal structure and biogenesis	582031	582573	101	1.97	2.08e-5
plu0526	CDS	-	conserved hypothetical protein			Unclassified	582534	583718	96	2.95	5.10e-10
pluCDS05837CDS		-	-			Unclassified	583732	584238	153	1.48	6.70e-4
plu0527	CDS	-	conserved hypothetical protein	COG3220	S	Function unknown	584213	584992	151	2.89	2.19e-12
plu0528	CDS	-	Conserved hypothetical protein	COG3220	S	Function unknown	584996	586072	419	3.69	1.42e-29
plu0549	CDS	-	CdiB protein (TpsB family)	COG2831	U	Intracellular trafficking, secretion, and vesicular transport	618550	620268	37	-2.43	1.48e-4
plu0578	CDS	yaaH	-	COG1584	C	Energy production and conversion	654920	655489	260	-1.30	6.42e-5
plu0628	CDS	ygdB	-			Unclassified	712917	713402	181	2.21	5.10e-8
plu0634	CDS	-	Toxin secretion ABC transporter protein, HlyB family	COG2274	V	Defense mechanisms	726383	728518	2133	3.69	2.64e-46
plu0635	CDS	-	HlyD family secretion protein	COG0845	MV	Cell wall/membrane/envelope biogenesis, Defense mechanisms	728660	730075	589	3.85	9.48e-36
plu0636	CDS	-	conserved hypothetical protein			Unclassified	730185	730706	331	3.38	1.87e-23
plu0637	CDS	-	conserved hypothetical protein	COG0722	E	Amino acid transport and metabolism	730755	731276	640	3.68	2.52e-35
plu0638	CDS	-	conserved hypothetical protein			Unclassified	731335	731856	530	3.60	4.90e-31
plu0639	CDS	-	conserved hypothetical protein			Unclassified	731915	732445	698	3.72	7.24e-37
plu0640	CDS	-	Conserved hypothetical protein	COG5000	T	Signal transduction mechanisms	732504	733025	1231	3.78	1.83e-45
plu0641	CDS	-	conserved hypothetical protein			Unclassified	733074	733595	461	3.93	1.02e-35
plu0642	CDS	-	conserved hypothetical protein			Unclassified	733644	734165	1125	3.93	7.50e-48
plu0643	CDS	-	Putative transmembrane protein. The central region presents some similarities with proprotein convertase.	COG1404	O	Posttranslational modification, protein turnover, chaperones	734216	740587	7912	3.64	2.42e-54
plutRNA0744tRNA		-	transfert RNA-Met			Unclassified	744628	744704	740	-1.50	1.54e-7

**plu0691##**	**CDS**	**cutF**	**Copper homeostasis protein CutF precursor**	**COG3015**	**MV**	**Cell wall/membrane/envelope biogenesis, Defense mechanisms**	**794390**	**795061**	**3705**	**1.60**	**2.51e-10**
plu0697	CDS	metN	D-methionine transport ATP-binding protein MetN	COG1135	E	Amino acid transport and metabolism	799779	800810	345	-1.09	1.27e-3
plu0734	CDS	-	conserved hypothetical protein	COG1621	G	Carbohydrate transport and metabolism	841205	842320	95	-3.40	6.40e-13
plu0760	CDS	-	putative acid--CoA ligase and gramicidin S synthase 2	COG0318	IQ	Lipid transport and metabolism, Secondary metabolites biosynthesis, transport and catabolism	878406	879983	99	-1.82	1.76e-5
plu0761	CDS	-	-	COG0534	V	Defense mechanisms	880009	881409	137	-1.69	1.79e-5
plu0762	CDS	-	-	COG1960	I	Lipid transport and metabolism	881414	882493	239	-1.14	4.02e-4
plu0763	CDS	-	hypothetical protein			Unclassified	882486	883160	177	-2.12	7.13e-9
plu0764	CDS	-	hypothetical protein	COG0332	I	Lipid transport and metabolism	883171	884049	353	-2.71	1.90e-17
plu0765	CDS	-	putative acyl carrier protein	COG0236	IQ	Lipid transport and metabolism, Secondary metabolites biosynthesis, transport and catabolism	884051	884314	249	-2.68	6.96e-14
plu0786	CDS	-	Conserved hypothetical protein. Probable transmembrane protein with 3 candidate membrane-spanning segments.	COG3539	N	Cell motility	905365	905925	639	-5.19	4.96e-57
plu0787	CDS	-	putative fimbrial chaperone	COG3121	W	Extracellular structures	906027	906743	55	-2.60	3.13e-7
plu0788	CDS	-	conserved hypothetical protein	COG3539	N	Cell motility	906743	907315	31	-2.90	6.24e-6
plu0789	CDS	-	putative fimbrial chaperone	COG3121	W	Extracellular structures	907349	908098	29	-2.31	2.51e-4
plu0824	CDS	-	Some similarities with gp31 protein of Bacteriophage			Unclassified	955418	956575	122	1.27	4.46e-3
plu0842	CDS	speD	S-adenosylmethionine decarboxylase proenzyme	COG1586	E	Amino acid transport and metabolism	973875	974669	644	1.43	8.83e-7
plu0843	CDS	speE	spermidine synthase (putrescine aminopropyltransferase)	COG0421	E	Amino acid transport and metabolism	974708	975577	948	2.02	8.62e-14
plu0855	CDS	-	-	COG0845	MV	Cell wall/membrane/envelope biogenesis, Defense mechanisms	985470	985898	75	-2.39	2.26e-6
plu0856	CDS	-	conserved hypothetical protein	COG2850	J	Translation, ribosomal structure and biogenesis	985888	987054	330	-3.67	1.85e-24
plu0857	CDS	-	-			Unclassified	987214	987588	1049	-2.33	2.33e-17
**plu0858#**	**CDS**	**-**	**Conserved hypothetical protein; putative transmembrane protein**			**Unclassified**	**987602**	**988066**	**23**	**-4.97**	**9.24e-9**
plu0884	CDS	-	conserved hypothetical protein	COG3266	D	Cell cycle control, cell division, chromosome partitioning	1012869	1014542	2543	1.33	5.35e-8
plu0885	CDS	-	putative pyocin S3 immunity protein			Unclassified	1014542	1014994	3375	1.20	1.07e-7
plu0886	CDS	-	Putative pyocin S3 immunity protein			Unclassified	1015076	1015528	551	1.21	1.53e-5
plu0887	CDS	-	C-terminal region of klebicin B, pyocin S2 and the killer protein of pyocin S1	COG4104	U	Intracellular trafficking, secretion, and vesicular transport	1015625	1016074	430	1.34	6.28e-6
plu0888	CDS	-	putative colicin / pyocin immunity protein			Unclassified	1016082	1016333	713	1.46	3.60e-8
plu0902	CDS	hemL	glutamate-1-semialdehyde 2,1-aminomutase	COG0001	H	Coenzyme transport and metabolism	1036929	1038212	1225	1.45	1.07e-7
plu0903	CDS	-	conserved hypothetical protein	COG0657	I	Lipid transport and metabolism	1038263	1039048	862	1.85	1.47e-10
plu0932	CDS	-	hypothetical protein	COG1708	R	General function prediction only	1073495	1074538	152	4.07	8.51e-22
plu0933	CDS	-	Conserved hypothetical protein			Unclassified	1074544	1075578	60	4.50	5.89e-15
plu0934	CDS	-	-	COG0483	G	Carbohydrate transport and metabolism	1075575	1076420	24	4.18	6.00e-8
plu0935	CDS	-	Putative biotin synthase-related enzyme	COG2516	R	General function prediction only	1076423	1077556	48	3.10	2.60e-8
plu0961	CDS	tcdB1	B component of insecticidal toxin complex, protein TcdB1	COG3209	R	General function prediction only	1103029	1107459	212	1.80	6.76e-7
plu0962	CDS	tcdA1	A component of insecticidal toxin complex, protein TcdA1	COG0497	L	Replication, recombination and repair	1107512	1115089	423	2.00	3.31e-11
plu0969	CDS	tcdB2	B component of insecticidal toxin complex, protein TcdB2	COG3209	R	General function prediction only	1132074	1136501	105	2.70	7.36e-10
plu0970	CDS	tcdA2	A component of insecticidal toxin complex, protein TcdA1	COG0497	L	Replication, recombination and repair	1136558	1143892	264	3.48	1.30e-23
plu0973	CDS	-	putative di-tripeptide transporter protein YhiP of Escherichia coli	COG3104	E	Amino acid transport and metabolism	1152675	1154144	310	1.97	1.72e-10
plu0994	CDS	phfA	PhfA protein; adhesion protein; fimbriae	COG3539	N	Cell motility	1177360	1178340	22	-3.86	6.29e-8
plu0995	CDS	phfD	Fimbria; Membrane; Outer membrane; Transmembrane; Transport	COG3188	NW	Cell motility, Extracellular structures	1178368	1180881	155	-4.10	3.35e-21
plu0996	CDS	phfC	PhfC protein, putative adhesion protein, fimbriae	COG3121	W	Extracellular structures	1180887	1181555	105	-4.54	1.64e-20
plu0997	CDS	phfS	PhfC protein, putative adhesion protein, fimbriae	COG3539	N	Cell motility	1181612	1182121	609	-4.37	1.66e-44
plu1010	CDS	-	hypothetical protein			Unclassified	1195389	1195637	81	1.58	8.97e-4
plu1012	CDS	-	conserved hypothetical protein	COG5441	S	Function unknown	1196502	1197746	694	1.16	2.47e-5
plu1149	CDS	-	CdiA protein (TpsA-related family); presence of the VENN motif and DUF638 domain	COG3210	U	Intracellular trafficking, secretion, and vesicular transport	1327789	1336602	7344	2.53	3.65e-28
plu1150	CDS	-	CdiB protein (TpsB family)	COG2831	U	Intracellular trafficking, secretion, and vesicular transport	1336651	1338315	2726	2.33	2.52e-23
plu1191	CDS	lysA	diaminopimelate decarboxylase (DAP decarboxylase)	COG0019	E	Amino acid transport and metabolism	1373105	1374415	1126	-2.62	1.57e-24
plu1192	CDS	fadE	-	COG1960	I	Lipid transport and metabolism	1374488	1376923	128	1.46	1.24e-3
plu1234	CDS	-	putative exported protein	COG0584	I	Lipid transport and metabolism	1426574	1427470	60	1.75	9.01e-4
plu1241	CDS	gpt	xanthine-guanine phosphoribosyltransferase (XGPRT)	COG2236	H	Coenzyme transport and metabolism	1435168	1435629	698	-1.01	4.57e-3
plu1242	CDS	yafA	-	COG1073	T	Signal transduction mechanisms	1435644	1436891	750	-1.06	1.10e-3
plu1266	CDS	yfiA	Highly similar to probable sigma-54 modulation protein YfiA of Escherichia coli	COG1544	J	Translation, ribosomal structure and biogenesis	1466407	1466769	92	1.95	3.37e-5
plu1306	CDS	gltJ	Glutamate/aspartate transport system permease protein GltJ	COG0765	E	Amino acid transport and metabolism	1514978	1515718	114	1.67	4.05e-4
plu1307	CDS	gltI	Glutamate/aspartate transport system permease protein GltI	COG0834	ET	Amino acid transport and metabolism, Signal transduction mechanisms	1515851	1516744	579	2.04	1.03e-12
pluCDS16291	CDS	-	-			Unclassified	1629184	1629867	697	2.26	5.21e-18
plu1367	CDS	-	CdiA protein (TpsA-related family); presence of the VENN motif and DUF638 domain	COG3210	U	Intracellular trafficking, secretion, and vesicular transport	1629806	1638691	3391	2.92	5.98e-35
plu1368	CDS	-	CdiB protein (TpsB family)	COG2831	U	Intracellular trafficking, secretion, and vesicular transport	1638740	1640404	495	3.01	2.76e-24
plu1382	CDS	prtS	Extracellular M4 metalloprotease precursor PrtS	COG3227	O	Posttranslational modification, protein turnover, chaperones	1661809	1662906	1566	-2.16	1.46e-17
plutRNA1683	tRNA	-	transfert RNA-Val			Unclassified	1683915	1683990	156	-1.49	1.11e-3
plutRNA1684	tRNA	-	transfert RNA-Lys			Unclassified	1684372	1684447	474	-1.05	3.44e-3
plu1409	CDS	-	putative MFS transporter	COG2814	G	Carbohydrate transport and metabolism	1691868	1693100	398	-2.07	1.43e-10
plu1410	CDS	-	conserved hypothetical protein	COG1475	D	Cell cycle control, cell division, chromosome partitioning	1693066	1693461	164	-2.20	4.14e-8
plu1411	CDS	-	-	COG0399	M	Cell wall/membrane/envelope biogenesis	1693448	1694650	370	-2.46	2.12e-14
plu1412	CDS	-	putative hemolysin erythrocyte lysis protein 2	COG0079	E	Amino acid transport and metabolism	1694887	1696743	260	-2.65	1.15e-13
plu1413	CDS	-	-	COG0223	J	Translation, ribosomal structure and biogenesis	1696740	1697414	135	-1.87	9.71e-6
plu1414	CDS	-	putative hydrolase	COG0561	HR	Coenzyme transport and metabolism, General function prediction only	1697422	1698249	496	-1.15	4.95e-4
plu1415	CDS	-	Protein containing Membrane Attack Complex/Perforin (MACPF) domains			Unclassified	1698682	1700214	31	-1.88	3.31e-3
plu1419	CDS	kdpB	potassium-transporting ATPase B chain	COG2216	P	Inorganic ion transport and metabolism	1704464	1706530	40	1.82	6.40e-4
plu1420	CDS	kdpA	potassium-transporting ATPase A chain	COG2060	P	Inorganic ion transport and metabolism	1706551	1708257	72	1.85	7.93e-5
plutRNA1750	tRNA	-	transfert RNA-Lys			Unclassified	1749948	1750023	935	-1.39	9.16e-7
plutRNA1750	tRNA	-	transfert RNA-Lys			Unclassified	1750058	1750133	216	-1.51	1.42e-4
plu1504	CDS	-	Putative 2-amino-3-ketobutyrate coenzyme A ligase	COG0156	H	Coenzyme transport and metabolism	1801468	1802814	375	-2.12	3.54e-10
plu1515	CDS	-	Similar to MccB protein of Escherichia coli, involved in the production of microcin C7	COG0476	H	Coenzyme transport and metabolism	1814537	1815595	134	1.99	1.19e-6
plu1516	CDS	-	Lipase	COG3675	I	Lipid transport and metabolism	1815904	1817010	290	-1.41	5.46e-4
plu1519	CDS	-	Lipase	COG3675	I	Lipid transport and metabolism	1820645	1821769	563	-1.74	9.34e-8
plu1534	CDS	-	DNA protection during starvation protein	COG0783	PV	Inorganic ion transport and metabolism, Defense mechanisms	1835229	1835576	40	-2.47	8.47e-7
plu1537	CDS	pit	Insecticidal crystal-related protein Pit			Unclassified	1838326	1838736	5919	-2.04	9.27e-17
plu1561	CDS	-	Putative calcium-dependent cell adhesion molecule-1 of Dictyostelium discoideum			Unclassified	1866612	1867232	1030	-2.28	9.76e-17
**plu1575#**	**CDS**	**-**	**Similar to crystalline inclusion protein type II (CipA-like protein)**			**Unclassified**	**1882485**	**1882823**	**2740**	**-4.58**	**2.00e-68**
plu1576	CDS	cipA	Crystalline inclusion protein CipA			Unclassified	1883974	1884288	179	-3.41	1.14e-15
**plu1579##**	**CDS**	**-**	**putative bacitracin transport permease YbjG of Escherichia coli**	**COG0671**	**I**	**Lipid transport and metabolism**	**1888106**	**1888714**	**741**	**1.82**	**2.45e-10**
plu1592	CDS	cspD	Cold shock-like protein CspD	COG1278	K	Transcription	1899373	1899594	192	1.79	3.02e-6
plu1651	CDS	-	-			Unclassified	1969768	1970754	22	-2.27	1.61e-3
plu1654	CDS	-	conserved hypothetical protein	COG1196	D	Cell cycle control, cell division, chromosome partitioning	1973894	1975858	7.03	-2.78	3.59e-3

plu1665	CDS	-	conserved hypothetical protein	COG3497	X	Mobilome: prophages, transposons	1988466	1989899	95	-3.17	2.35e-11
plu1666	CDS	-	conserved hypothetical protein	COG3497	X	Mobilome: prophages, transposons	1989911	1990993	72	-2.97	2.61e-10
plu1667	CDS	-	conserved hypothetical protein	COG0399	M	Cell wall/membrane/envelope biogenesis	1991047	1991496	39	-3.82	5.34e-9
plu1694	CDS	-	conserved hypothetical protein			Unclassified	2021795	2023357	11	2.79	1.69e-3
plu1697	CDS	-	conserved hypothetical protein	COG3422	S	Function unknown	2025763	2028669	28	2.00	2.12e-3
plu1698	CDS	-	conserved hypothetical protein	COG3299	X	Mobilome: prophages, transposons	2028666	2031368	30	1.95	2.42e-3
plu1706	CDS	-	conserved hypothetical protein	COG3497	X	Mobilome: prophages, transposons	2036552	2038036	265	3.50	2.37e-22
plu1707	CDS	-	conserved hypothetical protein	COG3497	X	Mobilome: prophages, transposons	2038048	2039124	148	3.92	4.44e-20
plu1708	CDS	-	conserved hypothetical protein	COG0399	M	Cell wall/membrane/envelope biogenesis	2039178	2039627	55	4.24	4.78e-14
plu1780	CDS	mgsA	methylglyoxal synthase	COG1803	G	Carbohydrate transport and metabolism	2125558	2126016	53	1.82	1.66e-3
plu1808	CDS	-	hypothetical protein			Unclassified	2156934	2157293	189	1.32	1.16e-3
plu1813	CDS	-	putative di-tripeptide ABC transporter	COG3104	E	Amino acid transport and metabolism	2159566	2161104	120	2.50	3.71e-9
plutRNA218[illegible]	tRNA	-	transfert RNA-Asn			Unclassified	2186131	2186206	51	-2.67	3.65e-7
plu1840	CDS	-	Conserved hypothetical protein			Unclassified	2187702	2188778	147	-1.89	2.74e-5
plu1864	CDS	-	Truncated gene. Putative phosphoenolpyruvate phosphomutase	COG0615	M	Cell wall/membrane/envelope biogenesis	2210011	2210184	98	3.96	3.15e-17
plu1865	CDS	-	Truncated gene. Putative phosphoenolpyruvate phosphomutase	COG2513	G	Carbohydrate transport and metabolism	2210256	2210996	57	2.90	1.15e-8
plu1866	CDS	-	Similar to nitrilotriacetate monooxygenase component A	COG2141	HR	Coenzyme transport and metabolism, General function prediction only	2211178	2212509	73	3.57	9.74e-12
plu1867	CDS	-	Truncated gene, similar to N-terminal of homocitrate synthase	COG0119	E	Amino acid transport and metabolism	2212546	2213232	61	2.92	1.01e-8
plu1868	CDS	-	Truncated gene, similar to C-terminal of homocitrate synthase	COG0119	E	Amino acid transport and metabolism	2213239	2213682	38	2.84	1.00e-6
plu1869	CDS	-	Similar to large subunit of 3-isopropylmalate dehydratase, large subunit	COG0065	E	Amino acid transport and metabolism	2213679	2214920	151	1.92	7.66e-7
plu1870	CDS	-	Similar to 3-isopropylmalate dehydratase, small subunit (leuD-2)	COG0066	E	Amino acid transport and metabolism	2214913	2215470	43	1.62	3.58e-3
plu1871	CDS	-	Putative methyltransferases	COG2226	H	Coenzyme transport and metabolism	2215467	2216252	53	1.80	8.09e-4
plu1882	CDS	sylC	Similar to non-ribosomal peptide synthetase (NRPS)			Unclassified	2240985	2242391	213	1.24	1.22e-3
plu1957	CDS	putA	proline dehydrogenase (Proline oxidase) / delta-1-pyrroline-5-carboxylate dehydrogenase (p5c dehydrogenase)	COG0506	E	Amino acid transport and metabolism	2322639	2326619	1675	1.33	1.26e-7
plu1958	CDS	putP	Sodium/proline symporter (Proline permease)	COG0591	E	Amino acid transport and metabolism	2327106	2328590	3142	1.03	3.69e-5
plu1967	CDS	pagC	PagC-like membrane protein (virulence-related membrane protein) of Escherichia coli O157:H7	COG3637	M	Cell wall/membrane/envelope biogenesis	2339359	2339907	377	-1.16	1.14e-3
plu1968	CDS	dsdC	D-serine dehydratase (deaminase) transcriptional activator DsdC	COG0583	K	Transcription	2340217	2341155	146	-1.16	4.05e-3
plu1969	CDS	dsdX	DsdX permease	COG2610	GR	Carbohydrate transport and metabolism, General function prediction only	2341397	2342734	3063	-3.51	6.32e-53
plu1970	CDS	dsdA	D-serine dehydratase DsdA	COG3048	E	Amino acid transport and metabolism	2343419	2344750	2905	-2.35	1.22e-26
plu1975	CDS	-	putative xenobiotic reductase B and NAD(P)H-dependent 2-cyclohexen-1-one reductase homolog	COG1902	C	Energy production and conversion	2348123	2349178	296	2.23	5.54e-11
plu1979	CDS	sgcA	Putative phosphotransferase enzyme II, A component SgcA	COG1762	GT	Carbohydrate transport and metabolism, Signal transduction mechanisms	2351773	2352216	284	1.04	1.22e-3
plu1983	CDS	-	putative transcriptional regulator	COG2197	TK	Signal transduction mechanisms, Transcription	2354123	2354818	31	-2.39	1.37e-3
plu1984	CDS	-	-	COG2771	K	Transcription	2354999	2355448	45	-2.76	1.24e-6
plu1985	CDS	-	-	COG2771	K	Transcription	2355488	2355625	21	-3.12	2.33e-5
plu1986	CDS	-	hypothetical protein	COG2771	K	Transcription	2355775	2356209	156	-2.65	1.09e-10
plu1992	CDS	fruB	PTS system, fructose-specific IIA/FPr component	COG1925	TG	Signal transduction mechanisms, Carbohydrate transport and metabolism	2362665	2363792	488	3.37	4.01e-28
plu1993	CDS	fruA	PTS system, fructose-specific IIBC component	COG1299	G	Carbohydrate transport and metabolism	2363862	2365532	303	2.50	1.00e-13
plu1996	CDS	yhfX	-	COG3457	E	Amino acid transport and metabolism	2367087	2368265	698	1.50	4.19e-8
plu2008	CDS	-	putative transcriptional regulator, LuxR family	COG2197	TK	Signal transduction mechanisms, Transcription	2377769	2378449	39	-2.44	1.53e-4
plu2011	CDS	-	-	COG2197	TK	Signal transduction mechanisms, Transcription	2380505	2381185	108	-2.09	6.85e-5
plu2012	CDS	-	-	COG2197	TK	Signal transduction mechanisms, Transcription	2381346	2382026	147	-2.06	9.02e-6
plu2013	CDS	-	-	COG2197	TK	Signal transduction mechanisms, Transcription	2382053	2382733	135	-2.19	4.62e-6
plu2014	CDS	-	-	COG2197	TK	Signal transduction mechanisms, Transcription	2382718	2382864	77	-2.55	7.66e-7
plu2015	CDS	-	-	COG2197	TK	Signal transduction mechanisms, Transcription	2382894	2383571	245	-2.38	3.22e-9
plu2016	CDS	-	-	COG2197	TK	Signal transduction mechanisms, Transcription	2383627	2384319	520	-2.25	2.35e-11
plu2019	CDS	-	putative transcriptional regulator, LuxR family	COG2197	TK	Signal transduction mechanisms, Transcription	2387125	2387799	44	-2.03	1.01e-3
plu2023	CDS	-	putative tail fiber protein of a prophage	COG4675	S	Function unknown	2392707	2394188	161	-1.50	7.72e-4
plutRNA247[illegible]	tRNA	-	transfert RNA-Ser			Unclassified	2470432	2470521	514	-1.27	1.01e-4
plu2133	CDS	yeaY	-	COG3065	M	Cell wall/membrane/envelope biogenesis	2516428	2517006	877	1.24	1.71e-5
**plu2143#**	**CDS**	**-**	**hypothetical protein**			**Unclassified**	**2525733**	**2526353**	**1418**	**-1.94**	**1.74e-12**
plu2144	CDS	hslJ	Heat shock protein HslJ	COG3187	O	Posttranslational modification, protein turnover, chaperones	2526677	2527090	3227	-1.83	2.46e-13
plu2153	CDS	-	conserved hypothetical protein	COG1376	M	Cell wall/membrane/envelope biogenesis	2538015	2538377	84	3.37	9.33e-13
plu2162	CDS	ydgI	-	COG0531	E	Amino acid transport and metabolism	2546307	2547698	401	-1.09	1.90e-3
plu2191	CDS	-	Similarity with diaminobutyric acid aminotransferase	COG0160	E	Amino acid transport and metabolism	2576697	2580116	3267	3.51	2.40e-44
plu2192	CDS	-	Similar to molybdopterin and thiamine biosynthesis protein	COG0476	H	Coenzyme transport and metabolism	2580119	2581237	799	3.12	3.48e-28
plu2193	CDS	-	Putative gamma-butyrobetaine,2-oxoglutarate	COG2175	Q	Secondary metabolites biosynthesis, transport and catabolism	2581248	2582255	753	3.09	2.51e-27
plu2194	CDS	-	Similar to acetyltransferase	COG0456	J	Translation, ribosomal structure and biogenesis	2582304	2582744	389	3.27	1.45e-23
plu2195	CDS	-	Hypothetical protein	COG2843	M	Cell wall/membrane/envelope biogenesis	2582780	2583016	126	3.16	9.62e-13
plu2196	CDS	-	Similar to capsule biosynthesis protein	COG2843	M	Cell wall/membrane/envelope biogenesis	2582988	2583881	543	3.17	3.48e-25
plu2197	CDS	-	Similar to NikS protein involved in the biosynthesis of the peptidyl nucleoside antibiotic nikkomycin	COG0026	F	Nucleotide transport and metabolism	2583890	2585077	631	3.06	1.90e-25
plu2198	CDS	-	Similar to the phosphoribosylamine--glycine ligase	COG0151	F	Nucleotide transport and metabolism	2585118	2586380	558	3.02	2.31e-24
plu2199	CDS	-	Similar to permeases of the major facilitator superfamily	COG2271	G	Carbohydrate transport and metabolism	2586407	2587792	464	2.79	2.14e-20
plu2200	CDS	-	Similar to glutamyl-tRNA(Gln) amidotransferase chain A	COG0154	J	Translation, ribosomal structure and biogenesis	2587792	2589135	399	2.41	9.35e-15
plu2231	CDS	ynfD	-			Unclassified	2624343	2624606	695	1.37	1.47e-6
plu2232	CDS	ynfB	-	COG1508	K	Transcription	2624622	2624984	679	1.33	3.62e-6
plu2238	CDS	attM	NAHL lactonase ; Zn-metallohydrolase family	COG0491	R	General function prediction only	2632964	2633734	59	1.57	1.38e-3
plu2267	CDS	-	conserved hypothetical protein			Unclassified	2664517	2665476	50	-1.99	9.56e-4
plu2303	CDS	-	putative tail fiber protein	COG5301	X	Mobilome: prophages, transposons	2704727	2705602	20	-2.88	2.87e-4
plu2313	CDS	-	Lipase	COG2931	Q	Secondary metabolites biosynthesis, transport and catabolism	2709005	2710858	173	1.29	1.91e-3
plu2315	CDS	-	Probable transcriptional regulator of the luminbactin locus	COG2207	K	Transcription	2712189	2713160	263	-1.12	4.78e-3
plu2316	CDS	-	Luminbactin receptor	COG4771	P	Inorganic ion transport and metabolism	2713457	2715463	855	-1.10	3.28e-5
plu2326	CDS	txp40	Txp40, 40 kDa insecticidal protein (previously name A24tox)			Unclassified	2744886	2745893	81	3.33	7.68e-12
plu2336	CDS	-	-	COG1960	I	Lipid transport and metabolism	2751654	2753417	30	-2.10	1.58e-3
plu2337	CDS	-	hypothetical protein	COG1038	C	Energy production and conversion	2753942	2754256	317	-2.68	6.01e-15
plu2347	CDS	goaG	4-aminobutyrate aminotransferase (gamma-amino-N-butyrate transaminase) (GABA transaminase)	COG0160	E	Amino acid transport and metabolism	2764455	2765732	46	1.69	4.63e-3

plu2350	CDS	-	Oligopeptide ABC transporter; periplasmic binding protein	COG4166	E	Amino acid transport and metabolism	2768877	2770487	18	-2.68	2.95e-3
plu2355	CDS	-	hypothetical protein			Unclassified	2774021	2774488	58	-2.53	2.06e-6
plu2412	CDS	-	conserved hypothetical protein	COG1216	G	Carbohydrate transport and metabolism	2832866	2833933	193	1.06	1.84e-3
plu2420	CDS	-	-			Unclassified	2839044	2843954	262	1.82	6.68e-8
plu2453	CDS	-	Putative TpsA-related protein	COG3210	U	Intracellular trafficking, secretion, and vesicular transport	2880371	2885434	126	2.05	1.70e-6
**plu2480##**	**CDS**	**ail1**	**Ail protein precursor of Yersinia**	**COG3637**	**M**	**Cell wall/membrane/envelope biogenesis**	**2920701**	**2921228**	**3938**	**2.29**	**2.09e-21**
plu2481	CDS	ail2	Similar to Ail protein precursor of Yersinia	COG3637	M	Cell wall/membrane/envelope biogenesis	2921745	2922290	904	-3.09	5.55e-26
**plu2507#**	**CDS**	**ygbJ**	**Highly similar to hypothetical oxidoreductase YgbJ of Escherichia coli**	**COG2084**	**I**	**Lipid transport and metabolism**	**2951054**	**2951959**	**803**	**1.87**	**2.51e-9**
plu2508	CDS	ygbK	Function unknown; regulated by FlhDC and FliA; mutants are not defective in motility or chemotraxis (by similarity)	COG3395	S	Function unknown	2951959	2953236	607	2.05	3.54e-10
plu2509	CDS	ygbL	-	COG0235	G	Carbohydrate transport and metabolism	2953229	2953870	319	2.14	8.41e-9
plu2510	CDS	ygbM	-	COG3622	G	Carbohydrate transport and metabolism	2953889	2954668	439	2.01	4.73e-9
plu2511	CDS	ygbI	-	COG1349	KG	Transcription, Carbohydrate transport and metabolism	2954681	2955448	383	2.30	3.76e-11
plu2512	CDS	-	Putative nucleoside-diphosphate-sugar epimerase	COG0451	M	Cell wall/membrane/envelope biogenesis	2955621	2956586	441	2.58	1.68e-14
plu2513	CDS	-	-	COG2610	GR	Carbohydrate transport and metabolism, General function prediction only	2956589	2957974	549	2.27	7.53e-13
plu2534	CDS	-	conserved hypothetical protein; weak similarity with PirA of Photorhabdus luminescens Hm	COG4633	R	General function prediction only	2982682	2983026	3943	-2.63	1.38e-27
plu2560	CDS	dadX	Alanine racemase, catabolic	COG0787	M	Cell wall/membrane/envelope biogenesis	3004014	3005102	300	1.63	3.84e-7
plu2561	CDS	dadA	D-amino acid dehydrogenase small subunit	COG0665	E	Amino acid transport and metabolism	3005109	3006422	278	1.80	2.05e-8
plu2598	CDS	ydhH	-	COG2377	M	Cell wall/membrane/envelope biogenesis	3041021	3042139	371	1.71	2.52e-7
plu2599	CDS	slyB	Outer membrane lipoprotein SlyB precursor	COG3133	M	Cell wall/membrane/envelope biogenesis	3042481	3042951	4157	1.18	2.26e-6
plu2600	CDS	slyA	Transcriptional regulator protein SlyA	COG1846	K	Transcription	3043036	3043467	406	-1.20	4.55e-4
plu2609	CDS	-	-	COG2309	E	Amino acid transport and metabolism	3051950	3052927	167	1.19	1.10e-3
**pluCDS3053(**	**CDS**	**-**	**hypothetical protein**	**COG0469**	**G**	**Carbohydrate transport and metabolism**	**3053012**	**3053125**	**300**	**1.17**	**6.83e-5**
plu2634	CDS	-	putative hemin-binding protein	COG4558	P	Inorganic ion transport and metabolism	3084624	3085442	576	-1.32	3.29e-5
plu2637	CDS	-	hypothetical protein			Unclassified	3087284	3087898	767	-1.53	1.23e-7
plu2653	CDS	nlpC	Probable lipoprotein NlpC precursor	COG0791	M	Cell wall/membrane/envelope biogenesis	3107082	3107567	119	2.01	4.15e-6
plu2654	CDS	pbgE3	4-amino-4-deoxy-L-arabinose-phosphoundecaprenol flippase, PbgE3	COG2076	V	Defense mechanisms	3107673	3108053	196	2.33	2.79e-10
**plu2655#**	**CDS**	**pbgE2**	**4-amino-4-deoxy-L-arabinose-phosphoundecaprenol flippase, PbgE2**	**COG2076**	**V**	**Defense mechanisms**	**3108050**	**3108391**	**259**	**2.03**	**3.99e-9**
**plu2656#**	**CDS**	**pbgE1**	**4-amino-4-deoxy-L-arabinose transferase, PbgE1 protein**	**COG1807**	**M**	**Cell wall/membrane/envelope biogenesis**	**3108388**	**3110049**	**1447**	**2.29**	**1.06e-18**
**plu2657##**	**CDS**	**pbgP4**	**Conserved hypothetical protein PbgP4**	**COG0726**	**GM**	**Carbohydrate transport and metabolism, Cell wall/membrane/envelope biogenesis**	**3110057**	**3110950**	**2419**	**2.40**	**5.20e-22**
**plu2658##**	**CDS**	**pbgP3**	**UDP-L-Ara4N formyltransferase ; UDP-GlcA C-4'-decarboxylase PbgP3**	**COG0223**	**J**	**Translation, ribosomal structure and biogenesis**	**3110950**	**3112932**	**6214**	**2.44**	**1.95e-24**
**plu2659##**	**CDS**	**pbgP2**	**undecaprenyl phosphate-L-Ara4FN transferase PbgP2**	**COG0463**	**M**	**Cell wall/membrane/envelope biogenesis**	**3112933**	**3113910**	**1623**	**2.49**	**1.84e-22**
**plu2660##**	**CDS**	**pbgP1**	**UDP-4-amino-4-deoxy-L-arabinose--oxoglutarate aminotransferase PbgP1**	**COG0399**	**M**	**Cell wall/membrane/envelope biogenesis**	**3113911**	**3115056**	**2190**	**2.52**	**8.88e-24**
plu2689	CDS	ftnA	nonheme ferritin 1	COG1528	P	Inorganic ion transport and metabolism	3191952	3192455	2640	1.01	5.37e-6
plu2691	CDS	pip	proline iminopeptidase (PIP)	COG0596	HR	Coenzyme transport and metabolism, General function prediction only	3193177	3194130	1359	1.89	1.29e-15
plu2692	CDS	yebF	-			Unclassified	3194204	3194578	433	1.68	1.86e-8
plu2696	CDS	sdaA	L-serine dehydratase 1 (SDH 1)	COG1760	E	Amino acid transport and metabolism	3197110	3198474	396	-1.27	3.96e-5
plu2704	CDS	-	-	COG0846	O	Posttranslational modification, protein turnover, chaperones	3204991	3208869	309	1.16	1.53e-3
plu2779	CDS	yegP	-	COG3422	S	Function unknown	3297706	3298050	157	1.63	7.26e-5
**plu2784#**	**CDS**	**pagP**	**LipidA acyltransferase PagP**			**Unclassified**	**3305063**	**3305686**	**201**	**1.80**	**2.10e-6**
**plu2807#**	**CDS**	**phoP**	**Two-component response regulator PhoP**	**COG0745**	**TK**	**Signal transduction mechanisms, Transcription**	**3332475**	**3333143**	**302**	**1.49**	**5.42e-5**
plu2808	CDS	phoQ	Two-component sensor kinase PhoQ	COG0642	T	Signal transduction mechanisms	3333163	3334626	354	1.37	5.97e-5
plu2821	CDS	ndh	NADH dehydrogenase	COG1252	C	Energy production and conversion	3351843	3353147	430	-1.20	1.13e-5
plu2842	CDS	-	conserved hypothetical protein	COG0702	R	General function prediction only	3373241	3373873	304	1.37	2.11e-5
plu2849	CDS	yeeF	-	COG0531	E	Amino acid transport and metabolism	3380945	3382327	1457	-1.11	1.11e-4
plu2957	CDS	-	Similar to bacteriophage protein	COG4675	S	Function unknown	3460804	3461709	82	-2.51	4.29e-7
plu2958	CDS	-	putative tail fiber assembly protein	COG1881	R	General function prediction only	3462413	3462835	363	-2.28	2.30e-11
**plu2959#**	**CDS**	**-**	**Some similarities with bacteriophage protein**	**COG4675**	**S**	**Function unknown**	**3462838**	**3463425**	**462**	**-2.30**	**5.07e-13**
plu2960	CDS	-	Similar to bacteriophage tail fiber protein	COG4675	S	Function unknown	3463959	3464723	104	-3.36	4.42e-12
plu2961	CDS	-	putative bacteriophage tail fiber protein	COG4675	S	Function unknown	3465167	3466582	135	-2.42	2.03e-8
**plu2962#**	**CDS**	**-**	**putative bacteriophage tail fiber protein**	**COG4675**	**S**	**Function unknown**	**3466665**	**3468149**	**207**	**-3.26**	**3.39e-16**
plu2972	CDS	-	conserved hypothetical protein			Unclassified	3478972	3479619	224	-1.70	1.90e-5
plu2977	CDS	-	conserved hypothetical protein			Unclassified	3481924	3482754	48	-2.87	2.64e-7
plu2978	CDS	-	putative Hcp protein	COG3157	U	Intracellular trafficking, secretion, and vesicular transport	3482762	3483241	68	-4.01	1.83e-13
plu3001	CDS	-	conserved hypothetical protein	COG3591	E	Amino acid transport and metabolism	3502295	3503821	331	-1.96	1.10e-8
plutRNA3538	tRNA	-	transfert RNA-Thr			Unclassified	3538549	3538624	844	-1.21	4.59e-4
plu3044	CDS	ahpD	AhpD, carboxymuconolactone decarboxylase involved in 2,5-dihydrophenylalanine, precursor of 2,5-dihydroxystilbene	COG2128	P	Inorganic ion transport and metabolism	3543179	3543721	477	-1.14	7.00e-4
plu3067	CDS	-	conserved hypothetical protein	COG0654	HC	Coenzyme transport and metabolism, Energy production and conversion	3575125	3575667	1508	2.22	1.90e-17
plu3075	CDS	phaxA2	PhaxA2, similar to binary toxin XaxA of Xenorhabdus nematophila			Unclassified	3585640	3586866	40	-2.61	1.20e-5
plu3090	CDS	lrhA	Transcriptional regulator LrhA (HexA)	COG0583	K	Transcription	3603871	3604800	1403	-1.17	1.73e-6
plu3095	CDS	ackA	acetate kinase (acetokinase)	COG0282	C	Energy production and conversion	3611182	3612384	860	-1.62	4.90e-12
plu3096	CDS	pta	Phosphate acetyltransferase (phosphotransacetylase)	COG0280	C	Energy production and conversion	3612456	3614597	1549	-1.21	7.94e-9
plu3098	CDS	-	conserved hypothetical protein			Unclassified	3616168	3616842	84	-2.12	3.79e-5
plu3102	CDS	-	putative methyltransferase	COG2226	H	Coenzyme transport and metabolism	3621255	3622079	242	1.82	7.57e-8
plu3103	CDS	-	putative cysteine desulfurase (NifS protein homolog)	COG1104	E	Amino acid transport and metabolism	3622091	3623257	190	1.81	2.08e-6
plu3104	CDS	-	conserved hypothetical protein			Unclassified	3623257	3624141	265	2.46	8.03e-13
plu3107	CDS	astB	succinylarginine dihydrolase	COG3724	E	Amino acid transport and metabolism	3627587	3628930	43	1.87	7.58e-4
plu3108	CDS	astD	succinylglutamic semialdehyde dehydrogenase	COG1012	C	Energy production and conversion	3628981	3630456	109	2.21	4.99e-7
plu3109	CDS	astA	arginine N-succinyltransferase (AOST)	COG3138	E	Amino acid transport and metabolism	3630459	3631490	70	2.47	4.65e-7
plu3110	CDS	argM	succinylornithine transaminase (succinylornithine aminotransferase) (carbon starvation protein C)	COG4992	E	Amino acid transport and metabolism	3631512	3632720	102	2.75	1.29e-10
plu3120	CDS	-	Putative membrane protein	COG3209	R	General function prediction only	3639471	3643967	194	1.85	2.31e-6
plu3121	CDS	-	hypothetical protein			Unclassified	3643983	3644150	199	1.79	2.55e-6
plu3146	CDS	lsrB	ABC transporter Binding Protein (BP) LsrB	COG1879	G	Carbohydrate transport and metabolism	3709664	3710683	149	1.25	4.12e-3
plu3154	CDS	-	Hemolysin-coregulated protein (Hcp protein)	COG3157	U	Intracellular trafficking, secretion, and vesicular transport	3714422	3714901	481	1.40	2.19e-7
plu3159	CDS	pdl	putative lipase of Photorhabdus luminescens, strain W14	COG3675	I	Lipid transport and metabolism	3718571	3720448	135	-1.69	1.89e-4
plu3164	CDS	yfcG	-	COG0625	O	Posttranslational modification, protein turnover, chaperones	3724793	3725413	511	1.08	1.54e-3
plu3181	CDS	tctC	Probable tricarboxylate transport protein TctC	COG3181	C	Energy production and conversion	3742383	3743363	54	-1.55	3.67e-3

plu3201	CDS	fadI	Beta-ketoacyl-CoA thiolase, anaerobic beta-oxidation complex II; in FadR regulon (by similarity)	COG0183	I	Lipid transport and metabolism	3766624	3767934	152	1.41	1.16e-3
plu3204	CDS	-	-	COG3103	R	General function prediction only	3771375	3771728	2541	-1.38	1.28e-8
plu3219	CDS	-	Similar to probable transcription regulator, LuxR family	COG2771	K	Transcription	3807331	3808020	757	-1.69	2.74e-8
plu3220	CDS	-	Similar to probable transcription regulator, LuxR family	COG2197	TK	Signal transduction mechanisms, Transcription	3808060	3808746	871	-1.71	1.58e-8
plu3221	CDS	-	Similar to probable transcription regulator, LuxR family	COG2197	TK	Signal transduction mechanisms, Transcription	3809059	3809727	183	-2.21	9.46e-8
plu3287	CDS	treC	Trehalose-6-phosphate hydrolase (alpha,alpha-phosphotrehalase)	COG0366	G	Carbohydrate transport and metabolism	3901245	3902906	1241	1.88	1.30e-12
**plu3288#**	**CDS**	**treB**	**PTS system, trehalose-specific IIBC component (EIIBC-TRE) (trehalose permease IIBC component) (phosphotransferase enzyme II, BC component) (EII-TRE)**	**COG1263**	**G**	**Carbohydrate transport and metabolism**	**3903006**	**3904427**	**643**	**2.21**	**2.59e-14**
plu3330	CDS	-	conserved hypothetical protein	COG4675	S	Function unknown	3959757	3960584	100	-2.14	1.46e-5
plu3507	CDS	-	Putative major fimbrial sub-unit protein	COG3539	N	Cell motility	4099071	4099475	280	6.26	9.09e-47
plu3508	CDS	-	putative Fimbrial regulatory gene			Unclassified	4100136	4100411	68	4.61	2.92e-17
plu3509	CDS	-	conserved hypothetical protein	COG0456	J	Translation, ribosomal structure and biogenesis	4100619	4101074	166	1.81	3.70e-6
plu3539	CDS	prpE	Propionate--CoA ligase (Propionyl-CoA synthetase)	COG0365	I	Lipid transport and metabolism	4168817	4170703	213	1.18	4.20e-4
plu3540	CDS	prpD	2-methylcitrate dehydratase	COG2079	G	Carbohydrate transport and metabolism	4170776	4172227	254	1.43	3.21e-6
plu3541	CDS	prpC	2-methylcitrate synthase (Methylcitrate synthase) (Citrate synthase 2)	COG0372	C	Energy production and conversion	4172282	4173448	209	1.62	5.93e-7
plu3542	CDS	prpB	Probable methylisocitrate lyase (2-methylisocitrate lyase)	COG2513	G	Carbohydrate transport and metabolism	4173509	4174399	200	1.93	2.57e-9
plu3543	CDS	prpR	Propionate catabolism operon regulatory protein	COG3829	KT	Transcription, Signal transduction mechanisms	4174732	4176399	373	1.46	1.71e-5
plu3563	CDS	-	putative p-aminobenzoic acid synthase	COG0147	EH	Amino acid transport and metabolism, Coenzyme transport and metabolism	4198009	4200084	495	-1.37	1.75e-5
plu3565	CDS	-	putative class II aminotransferase and 5-aminolevulinic acid synthase	COG0156	H	Coenzyme transport and metabolism	4200468	4201742	188	-1.69	2.08e-5
plu3566	CDS	-	putative methylase and with protoporphyrinogen oxidase	COG2890	J	Translation, ribosomal structure and biogenesis	4201767	4202618	129	-2.37	3.15e-8
plu3567	CDS	-	putative N-formimidoyl fortimicin A synthase	COG0665	E	Amino acid transport and metabolism	4202615	4204048	211	-3.19	1.20e-16
plu3569	CDS	-	TpsB protein	COG2831	U	Intracellular trafficking, secretion, and vesicular transport	4208442	4210121	145	2.89	2.68e-12
plu3593	CDS	-	conserved hypothetical protein	COG3210	U	Intracellular trafficking, secretion, and vesicular transport	4235193	4236086	25	3.34	7.30e-7
plu3594	CDS	-	Putative TpsA-related protein	COG3210	U	Intracellular trafficking, secretion, and vesicular transport	4236073	4242480	4387	3.58	7.76e-50
plu3595	CDS	-	-	COG2831	U	Intracellular trafficking, secretion, and vesicular transport	4242432	4242791	384	3.48	8.52e-26
plu3596	CDS	gcvP	glycine dehydrogenase [decarboxylating] (glycine decarboxylase) (glycine cleavage system P-protein)	COG0403	E	Amino acid transport and metabolism	4243413	4246289	1528	1.76	7.96e-13
plu3597	CDS	gcvH	glycine cleavage system H protein	COG0509	E	Amino acid transport and metabolism	4246377	4246769	828	1.12	6.57e-5
plu3598	CDS	gcvT	aminomethyltransferase (glycine cleavage system T protein)	COG0404	E	Amino acid transport and metabolism	4246891	4247985	973	1.45	3.70e-8
plu3611	CDS	yggE	-	COG2968	S	Function unknown	4259005	4259727	5876	-1.25	5.93e-7
plu3622	CDS	aceF	dihydrolipoamide acetyltransferase component of pyruvate dehydrogenase complex (E2)	COG0508	C	Energy production and conversion	4271312	4272910	3617	-1.53	2.85e-13
plu3623	CDS	aceE	pyruvate dehydrogenase E1 component	COG2609	C	Energy production and conversion	4272925	4275588	6728	-1.83	3.66e-18
plu3624	CDS	pdhR	pyruvate dehydrogenase complex repressor	COG2186	K	Transcription	4275778	4276542	681	-2.36	9.09e-20
plu3625	CDS	-	hypothetical protein			Unclassified	4277274	4277642	324	1.20	4.39e-4
plu3626	CDS	-	-	COG0472	M	Cell wall/membrane/envelope biogenesis	4277690	4278037	134	1.53	3.16e-4
plu3627	CDS	-	putative transcription regulator.	COG3655	K	Transcription	4278307	4278528	295	1.14	3.42e-4
plu3628	CDS	-	conserved hypothetical protein	COG1086	MO	Cell wall/membrane/envelope biogenesis, Posttranslational modification, protein turnover, chaperones	4278537	4279115	843	1.53	3.98e-8
plu3630	CDS	-	Conserved hypothetical protein	COG3539	N	Cell motility	4280090	4280476	144	5.03	1.21e-26
pluCDS42807	CDS	-	hypothetical protein			Unclassified	4280440	4280703	115	5.30	4.11e-25
plu3631	CDS	-	putative fimbrial chaperone YcbR precursor of Escherichia coli	COG3121	W	Extracellular structures	4280806	4281522	98	4.73	9.09e-20
plu3632	CDS	-	-	COG3539	N	Cell motility	4281533	4281748	30	3.98	1.67e-9
plu3633	CDS	-	-	COG3539	N	Cell motility	4281700	4281996	28	4.22	1.17e-9
plu3634	CDS	-	putative the N-terminal region of outer membrane usher protein precursor	COG3188	NW	Cell motility, Extracellular structures	4282079	4283065	26	3.57	2.59e-7
plu3635	CDS	-	conserved hypothetical protein			Unclassified	4283163	4283507	7.42	3.30	7.20e-4
plu3669	CDS	-	conserved hypothetical protein	COG0790	T	Signal transduction mechanisms	4320004	4320630	187	-1.61	7.26e-5
plu3672	CDS	leuO	activator protein in leuABCD operon	COG0583	K	Transcription	4323714	4324658	265	-1.43	5.98e-5
plu3680	CDS	speB	agmatinase (agmatine ureohydrolase) (AUH)	COG0010	E	Amino acid transport and metabolism	4334743	4335666	865	-1.09	2.18e-4
plu3681	CDS	speA	biosynthetic arginine decarboxylase (ADC)	COG1166	E	Amino acid transport and metabolism	4335836	4337740	795	-1.38	3.77e-6
plu3682	CDS	-	hypothetical protein			Unclassified	4337867	4338079	438	-1.20	4.40e-4
plu3718	CDS	-	CdiA protein (TpsA-related family); presence of the VENN motif and DUF638 domain	COG3210	U	Intracellular trafficking, secretion, and vesicular transport	4374057	4383140	1683	3.27	4.86e-39
plu3719	CDS	-	CdiB protein (TpsB family)	COG2831	U	Intracellular trafficking, secretion, and vesicular transport	4383189	4384853	198	3.13	4.07e-17
plu3725	CDS	abgB	aminobenzoyl-glutamate utilization protein B	COG1473	R	General function prediction only	4391036	4392484	34	1.98	1.00e-3
plu3727	CDS	abgR	Putative transcriptional regulator of abgABT operon	COG0583	K	Transcription	4394074	4394973	94	2.23	4.75e-7
plu3739	CDS	aldB	Aldehyde dehydrogenase B (Lactaldehyde dehydrogenase)	COG1012	C	Energy production and conversion	4409211	4410695	1813	-1.23	8.43e-5
plu3740	CDS	-	putative sodium:alanine symporter and other amino acid transport proteins	COG1115	E	Amino acid transport and metabolism	4410741	4412108	670	1.36	6.34e-7
plu3767	CDS	sctN	Type III secretion component protein SctN	COG1157	NU	Cell motility, Intracellular trafficking, secretion, and vesicular transport	4434397	4435719	79	2.57	5.22e-7
plu3771	CDS	sctR	Type III secretion component protein SctR	COG4790	U	Intracellular trafficking, secretion, and vesicular transport	4438248	4438901	7.69	3.86	4.81e-4
plu3790	CDS	cspI	cold shock-like protein (CPS-I)	COG1278	K	Transcription	4452723	4452938	6615	-1.37	8.48e-9
plu3908	CDS	ggt	gamma-glutamyltranspeptidase	COG0405	E	Amino acid transport and metabolism	4579987	4581747	895	1.22	8.07e-6
plu3983	CDS	-	hypothetical protein			Unclassified	4670172	4670273	213	-1.43	6.98e-4
plu3991	CDS	sstT	Na+/serine (threonine) symporter	COG3633	E	Amino acid transport and metabolism	4678384	4679631	234	1.19	2.34e-3
pluCDS46868	CDS	-	-			Unclassified	4686529	4686873	433	-1.08	1.53e-4
plu4011	CDS	-	-			Unclassified	4702279	4702785	127	-1.51	1.28e-3
plu4084	CDS	-	putative transport protein and multidrug resistance protein like bicyclomycin resistance protein	COG2814	G	Carbohydrate transport and metabolism	4770938	4772125	249	1.36	2.61e-4
plu4089	CDS	fis	DNA-binding protein (Factor-for-inversion stimulation protein) (HIN recombinational enhancer binding protein)	COG2901	K	Transcription	4776705	4777001	4539	-1.05	2.37e-5
plu4168	CDS	tccB1	A component of insecticidal toxin complex (part 2), protein TccB1	COG1538	M	Cell wall/membrane/envelope biogenesis	4869362	4874056	737	1.04	4.61e-4
plu4169	CDS	tccA1	A component of insecticidal toxin complex (part 1), protein TccB1	COG3206	M	Cell wall/membrane/envelope biogenesis	4874151	4877051	482	1.49	1.38e-6
plu4172	CDS	-	putative pyocin S3 immunity protein and to Photorhabdus luminescens proteins			Unclassified	4878614	4879081	305	1.00	4.20e-3
plu4186	CDS	antI	Hydrolase/peptidase AntI involved in anthraquinone biosynthesis (type II polyketide synthase)	COG1073	T	Signal transduction mechanisms	4898888	4900042	547	-3.48	5.71e-29
plu4187	CDS	antH	Cyclase/aromatase AntH involved in anthraquinone biosynthesis (type II polyketide synthase)	COG2867	J	Translation, ribosomal structure and biogenesis	4900062	4901504	339	-2.67	8.14e-16
plu4188	CDS	antG	CoA ligase AntG involved in anthraquinone biosynthesis (type II polyketide synthase)	COG0318	IQ	Lipid transport and metabolism, Secondary metabolites biosynthesis, transport and catabolism	4901504	4903051	182	-3.69	5.46e-19
plu4189	CDS	antF	Acyl carrier protein AntF involved in anthraquinone biosynthesis (type II polyketide synthase)	COG0236	IQ	Lipid transport and metabolism, Secondary metabolites biosynthesis, transport and catabolism	4903075	4903323	96	-4.13	8.65e-17
plu4190	CDS	antE	Ketosynthase KS-beta AntE involved in anthraquinone biosynthesis (type II polyketide synthase)	COG0304	IQ	Lipid transport and metabolism, Secondary metabolites biosynthesis, transport and catabolism	4903358	4904473	263	-2.32	4.88e-11

plu4191	CDS	antD	Ketosynthase KS-alpha AntD involved in anthraquinone biosynthesis (type II polyketide synthase)	COG0304	IQ	Lipid transport and metabolism, Secondary metabolites biosynthesis, transport and catabolism	4904466	4905752	302	-3.68	1.33e-25
plu4192	CDS	ant C	Cyclase AntC involved in anthraquinone biosynthesis (type II polyketide synthase)	COG1878	E	Amino acid transport and metabolism	4906404	4907219	206	-4.20	4.11e-25
plu4193	CDS	antB	Phosphopantetheinyl transferase AntB involved in anthraquinone biosynthesis (type II polyketide synthase)	COG2091	H	Coenzyme transport and metabolism	4907212	4907925	295	-4.70	1.82e-34
plu4194	CDS	antA	Ketoreductase AntA involved in anthraquinone biosynthesis (type II polyketide synthase)	COG1028	IQR	Lipid transport and metabolism, Secondary metabolites biosynthesis, transport and catabolism, General function prediction only	4907928	4908704	460	-3.80	2.75e-33
**plu4210##**	**CDS**	**-**	**Conserved hypothetical protein with SANT/Myb DNA-binding domain (IPR001005)**	**COG0287**	**E**	**Amino acid transport and metabolism**	**4923248**	**4925854**	**554**	**1.46**	**4.75e-7**
**plu4211##**	**CDS**	**-**	**Putative Type VI secretion system effector, Hcp (IPR008514)**	**COG3157**	**U**	**Intracellular trafficking, secretion, and vesicular transport**	**4925955**	**4926446**	**1819**	**2.52**	**7.93e-26**
**plu4212##**	**CDS**	**-**	**Hypothetical protein**			**Unclassified**	**4926419**	**4926589**	**248**	**2.14**	**3.54e-10**
plu4213	CDS	-	Hypothetical protein			Unclassified	4926590	4926778	50	2.46	2.41e-6
**plu4214#**	**CDS**	**-**	**Putative type VI secretion system IcmF, C-terminal (domains IPR010623 and IPR009612)**	**COG3523**	**U**	**Intracellular trafficking, secretion, and vesicular transport**	**4927191**	**4930550**	**672**	**2.17**	**3.29e-14**
**plu4215#**	**CDS**	**-**	**Conserved hypothetical protein. Putative membrane protein.**	**COG3000**	**I**	**Lipid transport and metabolism**	**4930543**	**4931718**	**275**	**1.86**	**1.31e-7**
**plu4216#**	**CDS**	**-**	**Conserved hypothetical protein**	**COG4104**	**U**	**Intracellular trafficking, secretion, and vesicular transport**	**4931721**	**4931996**	**135**	**1.54**	**1.87e-4**
**plu4217#**	**CDS**	**-**	**Conserved hypothetical protein. Putative membrane protein**			**Unclassified**	**4932024**	**4932692**	**260**	**1.50**	**7.79e-6**
**plu4220##**	**CDS**	**-**	**Conserved hypothetical protein with SANT/Myb DNA-binding domain (IPR001005)**	**COG0287**	**E**	**Amino acid transport and metabolism**	**4934952**	**4937552**	**1320**	**1.14**	**9.94e-5**
**plu4221##**	**CDS**	**-**	**Conserved hypothetical protein**			**Unclassified**	**4937552**	**4938592**	**427**	**1.36**	**1.15e-4**
**plu4222##**	**CDS**	**-**	**Putative Type VI secretion system, RhsGE-associated Vgr protein (domain IPR006533)**	**COG3501**	**UXR**	**Intracellular trafficking, secretion, and vesicular transport, Mobilome: prophages, transposons, General function prediction only**	**4938603**	**4940981**	**907**	**1.33**	**3.63e-5**
**plu4223##**	**CDS**	**-**	**Putative type VI secretion ATPase, ClpV1 family**	**COG0542**	**O**	**Posttranslational modification, protein turnover, chaperones**	**4940978**	**4943659**	**1722**	**1.11**	**4.55e-4**
plu4230	CDS	-	conserved hypothetical protein			Unclassified	4952453	4953391	62	-3.38	1.21e-10
plu4238	CDS	-	conserved hypothetical protein			Unclassified	4960560	4961522	77	-4.28	9.27e-17
plu4241	CDS	-	-			Unclassified	4964137	4965018	45	-2.19	1.83e-4
plu4242	CDS	ppxA	photopexin A	COG1524	R	General function prediction only	4965343	4967037	35	-2.06	1.33e-3
plutRNA4975	tRNA	-	transfert RNA-Leu			Unclassified	4975653	4975739	223	-1.22	2.94e-3
**plu4291##**	**CDS**	**-**	**conserved hypothetical protein**	**COG3577**	**R**	**General function prediction only**	**5018093**	**5019289**	**644**	**2.05**	**5.07e-12**
plu4336	CDS	-	-	COG3670	Q	Secondary metabolites biosynthesis, transport and catabolism	5064178	5065650	82	-2.44	8.47e-7
plu4382	CDS	dltD	DltD protein for D-alanine esterification of lipoteichoic acid and wall teichoic acid	COG3966	M	Cell wall/membrane/envelope biogenesis	5116163	5117365	255	-1.97	1.14e-7
plu4383	CDS	dltB	DltB protein for D-alanine esterification of lipoteichoic acid and wall teichoic acid	COG1696	M	Cell wall/membrane/envelope biogenesis	5117509	5118633	72	-2.21	2.10e-5
plu4384	CDS	dltA	D-alanine-activating enzyme (DAE) (D-alanine-D-alanyl carrier protein ligase) (DCL)	COG1020	Q	Secondary metabolites biosynthesis, transport and catabolism	5118630	5120093	90	-1.68	1.13e-3
plu4393	CDS	-	conserved hypothetical protein	COG0823	U	Intracellular trafficking, secretion, and vesicular transport	5131421	5131987	117	-2.40	1.95e-8
plu4402	CDS	fadB	Fatty oxidation complex alpha subunit [includes: enoyl-CoA hydratase; delta(3)-cis-delta(2)-trans-enoyl-CoA isomerase; 3-hydroxyacyl-CoA dehydrogenase; 3-hydroxybutyryl-CoA epimerase]	COG1024	I	Lipid transport and metabolism	5149379	5151565	386	1.54	6.21e-7
plu4403	CDS	fadA	3-ketoacyl-CoA thiolase (fatty oxidation complex beta subunit) (beta-ketothiolase) (acetyl-CoA acyltransferase)	COG0183	I	Lipid transport and metabolism	5151577	5152743	256	1.37	1.24e-4
plu4418	CDS	-	putative pyrrolidone-carboxylate peptidase (5-oxoprolyl-peptidase) (Pyroglutamyl-peptidase I) (PGP-I) (Pyrase)	COG2039	O	Posttranslational modification, protein turnover, chaperones	5166814	5167470	104	-1.71	3.63e-4
plu4419	CDS	-	putative exported protein	COG3485	Q	Secondary metabolites biosynthesis, transport and catabolism	5167715	5168548	99	-1.92	2.51e-4
plu4424	CDS	-	putative MFS transporter	COG2814	G	Carbohydrate transport and metabolism	5175988	5177184	198	-2.44	5.27e-11
plu4496	CDS	-	conserved hypothetical protein	COG2509	R	General function prediction only	5252293	5253813	179	-3.01	3.92e-13
pluCDS52537	CDS	-	-	COG3335	X	Mobilome: prophages, transposons	5253759	5254148	136	-3.11	2.35e-12
plu4502	CDS	-	-	COG0023	J	Translation, ribosomal structure and biogenesis	5259368	5259619	1.21e+4	-1.10	3.25e-6
plu4523	CDS	deaD	inducible ATP-independent RNA helicase	COG0513	L	Replication, recombination and repair	5281393	5283327	4394	-1.15	6.34e-7
plu4540	CDS	obgE	DNA-binding GTPase involved in cell partioning; multicopy suppressor of ftsJ(rrmJ) (by similarity)	COG0536	DL	Cell cycle control, cell division, chromosome partitioning, Replication, recombination and repair	5302270	5303445	1651	-1.08	3.63e-5
plu4556	CDS	ytfL	-	COG1253	R	General function prediction only	5319488	5320819	253	1.32	1.50e-4
**plu4558#**	**CDS**	**ecfJ**	**Function unknown; rpoE regulon; periplasmic protein; expressed protein (by similarity)**	**COG3054**	**R**	**General function prediction only**	**5321428**	**5321982**	**1426**	**-1.21**	**1.66e-5**
plu4570	CDS	rplI	50S ribosomal subunit protein L9	COG0359	J	Translation, ribosomal structure and biogenesis	5336224	5336676	8280	-1.02	1.33e-4
plutRNA5359	tRNA	-	transfert RNA-Gly			Unclassified	5359645	5359720	980	-1.09	3.29e-4
plutRNA5359	tRNA	-	transfert RNA-Gly			Unclassified	5359917	5359992	378	-1.18	5.46e-4
plu4601	CDS	-	-	COG3501	UXR	Intracellular trafficking, secretion, and vesicular transport, Mobilome: prophages, transposons, General function prediction only	5368573	5370549	1438	1.35	2.16e-6
plu4602	CDS	-	Conserved hypothetical protein	COG1716	T	Signal transduction mechanisms	5370546	5371037	620	1.43	4.68e-6
plu4603	CDS	-	conserved hypothetical protein			Unclassified	5371040	5371384	341	1.29	4.00e-4
plu4604	CDS	-	conserved hypothetical protein			Unclassified	5371386	5371994	338	1.40	6.45e-5
plu4605	CDS	-	Conserved hypothetical protein			Unclassified	5372018	5372740	428	1.44	5.86e-6
plu4606	CDS	-	conserved hypothetical protein	COG4104	U	Intracellular trafficking, secretion, and vesicular transport	5372743	5373006	110	1.38	2.86e-3
plu4607	CDS	-	conserved hypothetical protein			Unclassified	5373170	5373802	270	1.46	7.69e-5
plu4608	CDS	-	Conserved hypothetical protein			Unclassified	5373851	5374585	395	1.52	8.62e-7
plu4609	CDS	-	conserved hypothetical protein	COG4104	U	Intracellular trafficking, secretion, and vesicular transport	5374588	5374851	145	1.54	5.76e-4
plu4611	CDS	-	conserved hypothetical protein			Unclassified	5375668	5376402	71	1.63	1.10e-3
plu4628	CDS	-	putative L-2,4-diaminobutyrate decarboxylase	COG0076	E	Amino acid transport and metabolism	5394901	5396445	104	-1.46	2.17e-4
plu4629	CDS	-	-	COG3486	Q	Secondary metabolites biosynthesis, transport and catabolism	5396476	5397777	66	-1.49	2.28e-3
plu4630	CDS	-	putative rhizobactin and aerobactin siderophores biosynthesis proteins RhsF and LucC	COG1670	JO	Translation, ribosomal structure and biogenesis, Posttranslational modification, protein turnover, chaperones	5397777	5400194	184	-1.07	4.18e-3
plu4668	CDS	ilvC	ketol-acid reductoisomerase (acetohydroxy-acidisomeroreductase) (alpha-keto-beta-hydroxylacil reductoisomerase)	COG0059	EH	Amino acid transport and metabolism, Coenzyme transport and metabolism	5443372	5444850	5347	-2.14	6.40e-24
**plu4748#**	**CDS**	**-**	**-**			**Unclassified**	**5528419**	**5529000**	**1453**	**-1.93**	**7.79e-13**
plu4758	CDS	rpmE	50S ribosomal subunit protein L31	COG0254	J	Translation, ribosomal structure and biogenesis	5539633	5539848	8211	-1.02	3.71e-5
plu4767	CDS	glpF	glycerol uptake facilitator protein	COG0580	G	Carbohydrate transport and metabolism	5548786	5549616	373	1.07	1.84e-3
plu4774	CDS	pfkA	6-phosphofructokinase isozyme I (phosphofructokinase-1) (phosphohexokinase-1)	COG0205	G	Carbohydrate transport and metabolism	5555554	5556525	828	1.24	3.19e-5
plu4777	CDS	ynbA	-	COG0558	I	Lipid transport and metabolism	5558482	5559156	170	1.32	1.59e-3
plu4793	CDS	cpxP	periplasmic protein precursor	COG3678	O	Posttranslational modification, protein turnover, chaperones	5566624	5567118	246	2.25	1.60e-11
plu4844	CDS	ppyS	ketosynthase responsible for production of pyrone, signaling molecule causing Photorhabdus cell clumping	COG0332	I	Lipid transport and metabolism	5617384	5618445	18	-2.18	2.94e-3
plu4873	CDS	-	conserved hypothetical protein			Unclassified	5645829	5646260	576	-1.19	3.39e-4
plu4892	CDS	-	-	COG0500	QR	Secondary metabolites biosynthesis, transport and catabolism, General function prediction only	5665879	5666832	36	-2.58	9.73e-5

Supplementary Table 3B: List of 208 genes underexpressed in *P. luminescens* TT01 between LB culture and LB+polyB culture (logarithmic phase, FDR≤0.005,-1≥ log2FC)
# indicates the genes belonging to the PhoP regulon in LB broth.
# # indicates the genes belonging to the core PhoP regulon.
* ratio LB+polyB/LB
Colors highlight some functional categories:
components of secretion system (33 genes)
insecticidal toxins (9 genes)
transporter (25 genes)
extracellular enzymes

Label	Type	Name	Product	COG_ID	COG_classID	COGClassDescription	Begin	End	normalized average read count	log2 fold change*	adjusted p-value (FDR)
plu0008	CDS	ogrK	Ogr protein; transcriptional activator for bacteriophage P2 late genes			Unclassified	10251	10472	45	-1.99	8.01e-4
plu0010	CDS	pts25	Phage tail protein	COG3499	X	Mobilome: prophages, transposons	11631	12095	71	-1.83	5.66e-4
plu0011	CDS	pts24	Phage tail protein	COG5283	X	Mobilome: prophages, transposons	12098	14536	382	-3.55	7.26e-26
plu0012	CDS	pts23	Phage tail protein			Unclassified	14517	14636	75	-5.20	6.86e-19
plu0013	CDS	pts22	Phage tail protein			Unclassified	14651	14968	404	-5.40	4.19e-49
plu0014	CDS	pts21	Major phage tail tube protein	COG3498	X	Mobilome: prophages, transposons	14995	15510	670	-5.12	1.47e-55
plu0015	CDS	pts20	Major phage tail sheath protein	COG3497	X	Mobilome: prophages, transposons	15521	16693	1243	-5.87	1.78e-81
plu0019	CDS	pts16	Tail fiber assembly protein	COG2110	J	Translation, ribosomal structure and biogenesis	18438	19034	127	-1.79	2.08e-5
plu0020	CDS	pts15	Alternative tail fiber protein of bacteriophage-related plasmid and bacteriophage	COG4675	S	Function unknown	19034	19522	89	-1.68	1.19e-3
plu0021	CDS	pts14	Tail fiber protein of bacteriophage	COG5301	X	Mobilome: prophages, transposons	19522	20649	145	-2.10	1.45e-6
plu0022	CDS	pts13	Phage tail protein	COG4385	X	Mobilome: prophages, transposons	20646	21260	82	-2.31	1.27e-6
plu0023	CDS	pts12	Phage baseplate assembly protein	COG3948	X	Mobilome: prophages, transposons	21253	22251	132	-2.59	4.16e-10
plu0024	CDS	pts11	Phage baseplate assembly protein	COG3628	X	Mobilome: prophages, transposons	22256	22597	54	-2.98	8.39e-9
plu0025	CDS	pts10	Phage-related baseplate assembly protein	COG4540	X	Mobilome: prophages, transposons	22597	23439	98	-3.47	6.24e-14
plu0026	CDS	pts9	Phage tail protein	COG5004	X	Mobilome: prophages, transposons	23936	24139	35	-3.96	1.48e-10
**plu0104#**	**CDS**	**-**	**Conserved hypothetical secreted protein**	**COG3193**	**S**	**Function unknown**	**101566**	**102060**	**166**	**-6.83**	**1.21e-36**
plu0157	CDS	cipB	Crystalline inclusion protein CipB			Unclassified	162599	162901	68	-2.21	4.00e-4
plu0158	CDS	amt	L-arginine:lysine amidinotransferase	COG1834	E	Amino acid transport and metabolism	163665	164765	79	-2.80	1.50e-8
plu0162	CDS	-	conserved hypothetical protein	COG1680	V	Defense mechanisms	168154	169746	218	-1.69	3.41e-5
plu0163	CDS	-	conserved hypothetical protein	COG1680	V	Defense mechanisms	169909	170412	8.83	-3.15	1.99e-3
plu0165	CDS	-	conserved hypothetical protein	COG1680	V	Defense mechanisms	172935	174524	92	-2.11	2.21e-5
plu0182	CDS	cpmA	CpmA protein involved in carbapenem biosynthesis	COG0367	E	Amino acid transport and metabolism	192436	193941	1786	-1.30	8.04e-7
plu0183	CDS	cpmB	CpmB protein involved in carbapenem biosynthesis	COG1024	I	Lipid transport and metabolism	193944	194693	1082	-1.09	5.50e-5
plu0184	CDS	cpmC	CpmC protein involved in carbapenem biosynthesis	COG2175	Q	Secondary metabolites biosynthesis, transport and catabolism	194707	195528	1562	-1.04	5.23e-5
plu0221	CDS	-	putative amino acid permease component of ABC transporter	COG0765	E	Amino acid transport and metabolism	227701	228444	551	-1.16	9.56e-4
plu0250	CDS	-	putative TRP-1 protein encoded by the toxA gene of Burkholderia glumae, methyltransferase involved in toxoflavin biosynthesis	COG4976	R	General function prediction only	267752	268495	64	-2.10	1.32e-4
plu0315	CDS	-	conserved hypothetical protein			Unclassified	333297	333845	67	-1.64	2.38e-3
plu0318	CDS	-	Putative AidA protein			Unclassified	341028	341618	145	-1.92	5.26e-5
plu0368	CDS	-	Putative Type VI secretion, VasB, ImpH, VC_A0111 (IPR010732 domain)	COG3520	U	Intracellular trafficking, secretion, and vesicular transport	395492	396472	244	-1.42	4.12e-5
plu0369	CDS	-	Putative type VI secretion system, VCA0110 (domain IPR010272)	COG3519	U	Intracellular trafficking, secretion, and vesicular transport	396508	398355	679	-1.25	8.14e-6
plu0370	CDS	-	Conserved hypothetical protein with basic-leucine zipper domain (IPR004827)	COG3518	U	Intracellular trafficking, secretion, and vesicular transport	398357	398797	248	-1.60	7.64e-6
plu0371	CDS	-	Putative type VI secretion protein, EvpB/VC_A0108, tail sheath (domain IPR010269)	COG3517	U	Intracellular trafficking, secretion, and vesicular transport	398804	400282	1379	-1.58	4.37e-10
plu0372	CDS	-	Putative Type VI secretion system, VipA, VC_A0107 or Hcp2 (domain IPR008312)	COG3516	U	Intracellular trafficking, secretion, and vesicular transport	400306	400803	679	-1.88	1.85e-11
plu0373	CDS	-	Putative Type VI secretion system effector, Hcp (IPR008514)	COG3157	U	Intracellular trafficking, secretion, and vesicular transport	401704	402222	5526	-2.01	1.25e-17
**plu0480#**	**CDS**	**-**	**Conserved hypothetical protein with Galactose-binding like domain (IPR008979 domain)**	**COG2273**	**G**	**Carbohydrate transport and metabolism**	**520584**	**521060**	**1908**	**-3.34**	**7.36e-39**
plu0549	CDS	-	CdiB protein (TpsB family)	COG2831	U	Intracellular trafficking, secretion, and vesicular transport	618550	620268	37	-2.43	1.48e-4
plu0578	CDS	yaaH	-	COG1584	C	Energy production and conversion	654920	655489	260	-1.30	6.42e-5
plutRNA0744	tRNA	-	transfert RNA-Met			Unclassified	744628	744704	740	-1.50	1.54e-7
plu0697	CDS	metN	D-methionine transport ATP-binding protein MetN	COG1135	E	Amino acid transport and metabolism	799779	800810	345	-1.09	1.27e-3
plu0734	CDS	-	conserved hypothetical protein	COG1621	G	Carbohydrate transport and metabolism	841205	842320	95	-3.40	6.40e-13
plu0760	CDS	-	putative acid--CoA ligase and gramicidin S synthase 2	COG0318	IQ	Lipid transport and metabolism, Secondary metabolites biosynthesis, transport and catabolism	878406	879983	99	-1.82	1.76e-5
plu0761	CDS	-	-	COG0534	V	Defense mechanisms	880009	881409	137	-1.69	1.79e-5
plu0762	CDS	-	-	COG1960	I	Lipid transport and metabolism	881414	882493	239	-1.14	4.02e-4
plu0763	CDS	-	hypothetical protein			Unclassified	882486	883160	177	-2.12	7.13e-9
plu0764	CDS	-	hypothetical protein	COG0332	I	Lipid transport and metabolism	883171	884049	353	-2.71	1.90e-17
plu0765	CDS	-	putative acyl carrier protein	COG0236	IQ	Lipid transport and metabolism, Secondary metabolites biosynthesis, transport and catabolism	884051	884314	249	-2.68	6.96e-14
plu0786	CDS	-	Conserved hypothetical protein. Probable transmembrane protein with 3 candidate membrane-spanning segments.	COG3539	N	Cell motility	905365	905925	639	-5.19	4.96e-57
plu0787	CDS	-	putative fimbrial chaperone	COG3121	W	Extracellular structures	906027	906743	55	-2.60	3.13e-7
plu0788	CDS	-	conserved hypothetical protein	COG3539	N	Cell motility	906743	907315	31	-2.90	6.24e-6
plu0789	CDS	-	putative fimbrial chaperone	COG3121	W	Extracellular structures	907349	908098	29	-2.31	2.51e-4
plu0855	CDS	-	-	COG0845	MV	Cell wall/membrane/envelope biogenesis, Defense mechanisms	985470	985898	75	-2.39	2.26e-6
plu0856	CDS	-	conserved hypothetical protein	COG2850	J	Translation, ribosomal structure and biogenesis	985888	987054	330	-3.67	1.85e-24
plu0857	CDS	-	-			Unclassified	987214	987588	1049	-2.33	2.33e-17
**plu0858#**	**CDS**	**-**	**Conserved hypothetical protein; putative transmembrane protein**			**Unclassified**	**987602**	**988066**	**23**	**-4.97**	**9.24e-9**
plu0994	CDS	phfA	PhfA protein; adhesion protein; fimbriae	COG3539	N	Cell motility	1177360	1178340	22	-3.86	6.29e-8

plu0995	CDS	phfD	Fimbria; Membrane; Outer membrane; Transmembrane; Transport	COG3188	NW	Cell motility, Extracellular structures	1178368	1180881	155	-4.10	3.35e-21
plu0996	CDS	phfC	PhfC protein, putative adhesion protein, fimbriae	COG3121	W	Extracellular structures	1180887	1181555	105	-4.54	1.64e-20
plu0997	CDS	phfS	PhfC protein, putative adhesion protein, fimbriae	COG3539	N	Cell motility	1181612	1182121	609	-4.37	1.66e-44
plu1191	CDS	lysA	diaminopimelate decarboxylase (DAP decarboxylase)	COG0019	E	Amino acid transport and metabolism	1373105	1374415	1126	-2.62	1.57e-24
plu1241	CDS	gpt	xanthine-guanine phosphoribosyltransferase (XGPRT)	COG2236	H	Coenzyme transport and metabolism	1435168	1435629	698	-1.01	4.57e-3
plu1242	CDS	yafA	-	COG1073	T	Signal transduction mechanisms	1435644	1436891	750	-1.06	1.10e-3
plu1382	CDS	prtS	Extracellular M4 metalloprotease precursor PrtS	COG3227	O	Posttranslational modification, protein turnover, chaperones	1661809	1662906	1566	-2.16	1.46e-17
plutRNA1683	tRNA	-	transfert RNA-Val			Unclassified	1683915	1683990	156	-1.49	1.11e-3
plutRNA1684	tRNA	-	transfert RNA-Lys			Unclassified	1684372	1684447	474	-1.05	3.44e-3
plu1409	CDS	-	putative MFS transporter	COG2814	G	Carbohydrate transport and metabolism	1691868	1693100	398	-2.07	1.43e-10
plu1410	CDS	-	conserved hypothetical protein	COG1475	D	Cell cycle control, cell division, chromosome partitioning	1693066	1693461	164	-2.20	4.14e-8
plu1411	CDS	-	-	COG0399	M	Cell wall/membrane/envelope biogenesis	1693448	1694650	370	-2.46	2.12e-14
plu1412	CDS	-	putative hemolysin erythrocyte lysis protein 2	COG0079	E	Amino acid transport and metabolism	1694887	1696743	260	-2.65	1.15e-13
plu1413	CDS	-	-	COG0223	J	Translation, ribosomal structure and biogenesis	1696740	1697414	135	-1.87	9.71e-6
plu1414	CDS	-	putative hydrolase	COG0561	HR	Coenzyme transport and metabolism, General function prediction only	1697422	1698249	496	-1.15	4.95e-4
plu1415	CDS	-	Protein containing Membrane Attack Complex/Perforin (MACPF) domains			Unclassified	1698682	1700214	31	-1.88	3.31e-3
plutRNA1750	tRNA	-	transfert RNA-Lys			Unclassified	1749948	1750023	935	-1.39	9.16e-7
plutRNA1750	tRNA	-	transfert RNA-Lys			Unclassified	1750058	1750133	216	-1.51	1.42e-4
plu1504	CDS	-	Putative 2-amino-3-ketobutyrate coenzyme A ligase	COG0156	H	Coenzyme transport and metabolism	1801468	1802814	375	-2.12	3.54e-10
plu1516	CDS	-	Lipase	COG3675	I	Lipid transport and metabolism	1815904	1817010	290	-1.41	5.46e-4
plu1519	CDS	-	Lipase	COG3675	I	Lipid transport and metabolism	1820645	1821769	563	-1.74	9.34e-8
plu1534	CDS	-	DNA protection during starvation protein	COG0783	PV	Inorganic ion transport and metabolism, Defense mechanisms	1835229	1835576	40	-2.47	8.47e-7
plu1537	CDS	pit	Insecticidal crystal-related protein Pit			Unclassified	1838326	1838736	5919	-2.04	9.27e-17
plu1561	CDS	-	Putative calcium-dependent cell adhesion molecule-1 of Dictyostelium discoideum			Unclassified	1866612	1867232	1030	-2.28	9.76e-17
**plu1575#**	**CDS**	**-**	**Similar to crystalline inclusion protein type II (CipA-like protein)**			**Unclassified**	**1882485**	**1882823**	**2740**	**-4.58**	**2.00e-68**
plu1576	CDS	cipA	Crystalline inclusion protein CipA			Unclassified	1883974	1884288	179	-3.41	1.14e-15
plu1651	CDS	-	-			Unclassified	1969768	1970754	22	-2.27	1.61e-3
plu1654	CDS	-	conserved hypothetical protein	COG1196	D	Cell cycle control, cell division, chromosome partitioning	1973894	1975858	7.03	-2.78	3.59e-3
plu1665	CDS	-	conserved hypothetical protein	COG3497	X	Mobilome: prophages, transposons	1988466	1989899	95	-3.17	2.35e-11
plu1666	CDS	-	conserved hypothetical protein	COG3497	X	Mobilome: prophages, transposons	1989911	1990993	72	-2.97	2.61e-10
plu1667	CDS	-	conserved hypothetical protein	COG0399	M	Cell wall/membrane/envelope biogenesis	1991047	1991496	39	-3.82	5.34e-9
plutRNA2186	tRNA	-	transfert RNA-Asn			Unclassified	2186131	2186206	51	-2.67	3.65e-7
plu1840	CDS	-	Conserved hypothetical protein			Unclassified	2187702	2188778	147	-1.89	2.74e-5
plu1967	CDS	pagC	PagC-like membrane protein (virulence-related membrane protein) of Escherichia coli O157:H7	COG3637	M	Cell wall/membrane/envelope biogenesis	2339359	2339907	377	-1.16	1.14e-3
plu1968	CDS	dsdC	D-serine dehydratase (deaminase) transcriptional activator DsdC	COG0583	K	Transcription	2340217	2341155	146	-1.16	4.05e-3
plu1969	CDS	dsdX	DsdX permease	COG2610	GR	Carbohydrate transport and metabolism, General function prediction only	2341397	2342734	3063	-3.51	6.32e-53
plu1970	CDS	dsdA	D-serine dehydratase DsdA	COG3048	E	Amino acid transport and metabolism	2343419	2344750	2905	-2.35	1.22e-26
plu1983	CDS	-	putative transcriptional regulator	COG2197	TK	Signal transduction mechanisms, Transcription	2354123	2354818	31	-2.39	1.37e-3
plu1984	CDS	-	-	COG2771	K	Transcription	2354999	2355448	45	-2.76	1.24e-6
plu1985	CDS	-	-	COG2771	K	Transcription	2355488	2355625	21	-3.12	2.33e-5
plu1986	CDS	-	hypothetical protein	COG2771	K	Transcription	2355775	2356209	156	-2.65	1.09e-10
plu2008	CDS	-	putative transcriptional regulator, LuxR family	COG2197	TK	Signal transduction mechanisms, Transcription	2377769	2378449	39	-2.44	1.53e-4
plu2011	CDS	-	-	COG2197	TK	Signal transduction mechanisms, Transcription	2380505	2381185	108	-2.09	6.85e-5
plu2012	CDS	-	-	COG2197	TK	Signal transduction mechanisms, Transcription	2381346	2382026	147	-2.06	9.02e-6
plu2013	CDS	-	-	COG2197	TK	Signal transduction mechanisms, Transcription	2382053	2382733	135	-2.19	4.62e-6
plu2014	CDS	-	-	COG2197	TK	Signal transduction mechanisms, Transcription	2382718	2382864	77	-2.55	7.66e-7
plu2015	CDS	-	-	COG2197	TK	Signal transduction mechanisms, Transcription	2382894	2383571	245	-2.38	3.22e-9
plu2016	CDS	-	-	COG2197	TK	Signal transduction mechanisms, Transcription	2383627	2384319	520	-2.25	2.35e-11
plu2019	CDS	-	putative transcriptional regulator, LuxR family	COG2197	TK	Signal transduction mechanisms, Transcription	2387125	2387799	44	-2.03	1.01e-3
plu2023	CDS	-	putative tail fiber protein of a prophage	COG4675	S	Function unknown	2392707	2394188	161	-1.50	7.72e-4
plutRNA2470	tRNA	-	transfert RNA-Ser			Unclassified	2470432	2470521	514	-1.27	1.01e-4
**plu2143#**	**CDS**	**-**	**hypothetical protein**			**Unclassified**	**2525733**	**2526353**	**1418**	**-1.94**	**1.74e-12**
plu2144	CDS	hslJ	Heat shock protein HslJ	COG3187	O	Posttranslational modification, protein turnover, chaperones	2526677	2527090	3227	-1.83	2.46e-13
plu2162	CDS	ydgI	-	COG0531	E	Amino acid transport and metabolism	2546307	2547698	401	-1.09	1.90e-3
plu2267	CDS	-	conserved hypothetical protein			Unclassified	2664517	2665476	50	-1.99	9.56e-4
plu2303	CDS	-	putative tail fiber protein	COG5301	X	Mobilome: prophages, transposons	2704727	2705602	20	-2.88	2.87e-4
plu2315	CDS	-	Probable transcriptional regulator of the luminbactin locus	COG2207	K	Transcription	2712189	2713160	263	-1.12	4.78e-3
plu2316	CDS	-	Luminbactin receptor	COG4771	P	Inorganic ion transport and metabolism	2713457	2715463	855	-1.10	3.28e-5
plu2336	CDS	-	-	COG1960	I	Lipid transport and metabolism	2751654	2753417	30	-2.10	1.58e-3
plu2337	CDS	-	hypothetical protein	COG1038	C	Energy production and conversion	2753942	2754256	317	-2.68	6.01e-15
plu2350	CDS	-	Oligopeptide ABC transporter; periplasmic binding protein	COG4166	E	Amino acid transport and metabolism	2768877	2770487	18	-2.68	2.95e-3
plu2355	CDS	-	hypothetical protein			Unclassified	2774021	2774488	58	-2.53	2.06e-6
plu2481	CDS	ail2	Similar to Ail protein precursor of Yersinia	COG3637	M	Cell wall/membrane/envelope biogenesis	2921745	2922290	904	-3.09	5.55e-26
plu2534	CDS	-	conserved hypothetical protein; weak similarity with PirA of Photorhabdus luminescens Hm	COG4633	R	General function prediction only	2982682	2983026	3943	-2.63	1.38e-27
plu2600	CDS	slyA	Transcriptional regulator protein SlyA	COG1846	K	Transcription	3043036	3043467	406	-1.20	4.55e-4
plu2634	CDS	-	putative hemin-binding protein	COG4558	P	Inorganic ion transport and metabolism	3084624	3085442	576	-1.32	3.29e-5

plu2637	CDS	-	hypothetical protein			Unclassified	3087284	3087898	767	-1.53	1.23e-7
plu2696	CDS	sdaA	L-serine dehydratase 1 (SDH 1)	COG1760	E	Amino acid transport and metabolism	3197110	3198474	396	-1.27	3.96e-5
plu2821	CDS	ndh	NADH dehydrogenase	COG1252	C	Energy production and conversion	3351843	3353147	430	-1.20	1.13e-5
plu2849	CDS	yeeF	-	COG0531	E	Amino acid transport and metabolism	3380945	3382327	1457	-1.11	1.11e-4
plu2957	CDS	-	Similar to bacteriophage protein	COG4675	S	Function unknown	3460804	3461709	82	-2.51	4.29e-7
plu2958	CDS	-	putative tail fiber assembly protein	COG1881	R	General function prediction only	3462413	3462835	363	-2.28	2.30e-11
**plu2959#**	**CDS**	**-**	**Some similarities with bacteriophage protein**	**COG4675**	**S**	**Function unknown**	**3462838**	**3463425**	**462**	**-2.30**	**5.07e-13**
plu2960	CDS	-	Similar to bacteriophage tail fiber protein	COG4675	S	Function unknown	3463959	3464723	104	-3.36	4.42e-12
plu2961	CDS	-	putative bacteriophage tail fiber protein	COG4675	S	Function unknown	3465167	3466582	135	-2.42	2.03e-8
**plu2962#**	**CDS**	**-**	**putative bacteriophage tail fiber protein**	**COG4675**	**S**	**Function unknown**	**3466665**	**3468149**	**207**	**-3.26**	**3.39e-16**
plu2972	CDS	-	conserved hypothetical protein			Unclassified	3478972	3479619	224	-1.70	1.90e-5
plu2977	CDS	-	conserved hypothetical protein			Unclassified	3481924	3482754	48	-2.87	2.64e-7
plu2978	CDS	-	putative Hcp protein	COG3157	U	Intracellular trafficking, secretion, and vesicular transport	3482762	3483241	68	-4.01	1.83e-13
plu3001	CDS	-	conserved hypothetical protein	COG3591	E	Amino acid transport and metabolism	3502295	3503821	331	-1.96	1.10e-8
plutRNA3538	tRNA	-	transfert RNA-Thr			Unclassified	3538549	3538624	844	-1.21	4.59e-4
plu3044	CDS	ahpD	AhpD, carboxymuconolactone decarboxylase involved in 2,5-dihydrophenylalanine, precursor of 2,5-dihydroxystilbene	COG2128	P	Inorganic ion transport and metabolism	3543179	3543721	477	-1.14	7.00e-4
plu3075	CDS	phaxA2	PhaxA2, similar to binary toxin XaxA of Xenorhabdus nematophila			Unclassified	3585640	3586866	40	-2.61	1.20e-5
plu3090	CDS	lrhA	Transcriptional regulator LrhA (HexA)	COG0583	K	Transcription	3603871	3604800	1403	-1.17	1.73e-6
plu3095	CDS	ackA	acetate kinase (acetokinase)	COG0282	C	Energy production and conversion	3611182	3612384	860	-1.62	4.90e-12
plu3096	CDS	pta	Phosphate acetyltransferase (phosphotransacetylase)	COG0280	C	Energy production and conversion	3612456	3614597	1549	-1.21	7.94e-9
plu3098	CDS	-	conserved hypothetical protein			Unclassified	3616168	3616842	84	-2.12	3.79e-5
plu3159	CDS	pdl	putative lipase of Photorhabdus luminescens, strain W14	COG3675	I	Lipid transport and metabolism	3718571	3720448	135	-1.69	1.89e-4
plu3181	CDS	tctC	Probable tricarboxylate transport protein TctC	COG3181	C	Energy production and conversion	3742383	3743363	54	-1.55	3.67e-3
plu3204	CDS	-	-	COG3103	R	General function prediction only	3771375	3771728	2541	-1.38	1.28e-8
plu3219	CDS	-	Similar to probable transcription regulator, LuxR family	COG2771	K	Transcription	3807331	3808020	757	-1.69	2.74e-8
plu3220	CDS	-	Similar to probable transcription regulator, LuxR family	COG2197	TK	Signal transduction mechanisms, Transcription	3808060	3808746	871	-1.71	1.58e-8
plu3221	CDS	-	Similar to probable transcription regulator, LuxR family	COG2197	TK	Signal transduction mechanisms, Transcription	3809059	3809727	183	-2.21	9.46e-8
plu3330	CDS	-	conserved hypothetical protein	COG4675	S	Function unknown	3959757	3960584	100	-2.14	1.46e-5
plu3563	CDS	-	putative p-aminobenzoic acid synthase	COG0147	EH	Amino acid transport and metabolism, Coenzyme transport and metabolism	4198009	4200084	495	-1.37	1.75e-5
plu3565	CDS	-	putative class II aminotransferase and 5-aminolevulinic acid synthase	COG0156	H	Coenzyme transport and metabolism	4200468	4201742	188	-1.69	2.08e-5
plu3566	CDS	-	putative methylase and with protoporphyrinogen oxidase	COG2890	J	Translation, ribosomal structure and biogenesis	4201767	4202618	129	-2.37	3.15e-8
plu3567	CDS	-	putative N-formimidoyl fortimicin A synthase	COG0665	E	Amino acid transport and metabolism	4202615	4204048	211	-3.19	1.20e-16
plu3611	CDS	yggE	-	COG2968	S	Function unknown	4259005	4259727	5876	-1.25	5.93e-7
plu3622	CDS	aceF	dihydrolipoamide acetyltransferase component of pyruvate dehydrogenase complex (E2)	COG0508	C	Energy production and conversion	4271312	4272910	3617	-1.53	2.85e-13
plu3623	CDS	aceE	pyruvate dehydrogenase E1 component	COG2609	C	Energy production and conversion	4272925	4275588	6728	-1.83	3.66e-18
plu3624	CDS	pdhR	pyruvate dehydrogenase complex repressor	COG2186	K	Transcription	4275778	4276542	681	-2.36	9.09e-20
plu3669	CDS	-	conserved hypothetical protein	COG0790	T	Signal transduction mechanisms	4320004	4320630	187	-1.61	7.26e-5
plu3672	CDS	leuO	activator protein in leuABCD operon	COG0583	K	Transcription	4323714	4324658	265	-1.43	5.98e-5
plu3680	CDS	speB	agmatinase (agmatine ureohydrolase) (AUH)	COG0010	E	Amino acid transport and metabolism	4334743	4335666	865	-1.09	2.18e-4
plu3681	CDS	speA	biosynthetic arginine decarboxylase (ADC)	COG1166	E	Amino acid transport and metabolism	4335836	4337740	795	-1.38	3.77e-6
plu3682	CDS	-	hypothetical protein			Unclassified	4337867	4338079	438	-1.20	4.40e-4
plu3739	CDS	aldB	Aldehyde dehydrogenase B (Lactaldehyde dehydrogenase)	COG1012	C	Energy production and conversion	4409211	4410695	1813	-1.23	8.43e-5
plu3790	CDS	cspI	cold shock-like protein (CPS-I)	COG1278	K	Transcription	4452723	4452938	6615	-1.37	8.48e-9
plu3983	CDS	-	hypothetical protein			Unclassified	4670172	4670273	213	-1.43	6.98e-4
pluCDS46868	CDS	-	-			Unclassified	4686529	4686873	433	-1.08	1.53e-4
plu4011	CDS	-	-			Unclassified	4702279	4702785	127	-1.51	1.28e-3
plu4089	CDS	fis	DNA-binding protein (Factor-for-inversion stimulation protein) (HIN recombinational enhancer binding protein)	COG2901	K	Transcription	4776705	4777001	4539	-1.05	2.37e-5
plu4186	CDS	antI	Hydrolase/peptidase AntI involved in anthraquinone biosynthesis (type II polyketide synthase)	COG1073	T	Signal transduction mechanisms	4898888	4900042	547	-3.48	5.71e-29
plu4187	CDS	antH	Cyclase/aromatase AntH involved in anthraquinone biosynthesis (type II polyketide synthase)	COG2867	J	Translation, ribosomal structure and biogenesis	4900062	4901504	339	-2.67	8.14e-16
plu4188	CDS	antG	CoA ligase AntG involved in anthraquinone biosynthesis (type II polyketide synthase)	COG0318	IQ	Lipid transport and metabolism, Secondary metabolites biosynthesis, transport and catabolism	4901504	4903051	182	-3.69	5.46e-19
plu4189	CDS	antF	Acyl carrier protein AntF involved in anthraquinone biosynthesis (type II polyketide synthase)	COG0236	IQ	Lipid transport and metabolism, Secondary metabolites biosynthesis, transport and catabolism	4903075	4903323	96	-4.13	8.65e-17
plu4190	CDS	antE	Ketosynthase KS-beta AntE involved in anthraquinone biosynthesis (type II polyketide synthase)	COG0304	IQ	Lipid transport and metabolism, Secondary metabolites biosynthesis, transport and catabolism	4903358	4904473	263	-2.32	4.88e-11
plu4191	CDS	antD	Ketosynthase KS-alpha AntD involved in anthraquinone biosynthesis (type II polyketide synthase)	COG0304	IQ	Lipid transport and metabolism, Secondary metabolites biosynthesis, transport and catabolism	4904466	4905752	302	-3.68	1.33e-25
plu4192	CDS	ant C	Cyclase AntC involved in anthraquinone biosynthesis (type II polyketide synthase)	COG1878	E	Amino acid transport and metabolism	4906404	4907219	206	-4.20	4.11e-25
plu4193	CDS	antB	Phosphopantetheinyl transferase AntB involved in anthraquinone biosynthesis (type II polyketide synthase)	COG2091	H	Coenzyme transport and metabolism	4907212	4907925	295	-4.70	1.82e-34
plu4194	CDS	antA	Ketoreductase AntA involved in anthraquinone biosynthesis (type II polyketide synthase)	COG1028	IQR	Lipid transport and metabolism, Secondary metabolites biosynthesis, transport and catabolism, General function prediction only	4907928	4908704	460	-3.80	2.75e-33
plu4230	CDS	-	conserved hypothetical protein			Unclassified	4952453	4953391	62	-3.38	1.21e-10
plu4238	CDS	-	conserved hypothetical protein			Unclassified	4960560	4961522	77	-4.28	9.27e-17
plu4241	CDS	-	-			Unclassified	4964137	4965018	45	-2.19	1.83e-4
plu4242	CDS	ppxA	photopexin A	COG1524	R	General function prediction only	4965343	4967037	35	-2.06	1.33e-3
plutRNA4975	tRNA	-	transfert RNA-Leu			Unclassified	4975653	4975739	223	-1.22	2.94e-3
plu4336	CDS	-	-	COG3670	Q	Secondary metabolites biosynthesis, transport and catabolism	5064178	5065650	82	-2.44	8.47e-7
plu4382	CDS	dltD	DltD protein for D-alanine esterification of lipoteichoic acid and wall teichoic acid	COG3966	M	Cell wall/membrane/envelope biogenesis	5116163	5117365	255	-1.97	1.14e-7
plu4383	CDS	dltB	DltB protein for D-alanine esterification of lipoteichoic acid and wall teichoic acid	COG1696	M	Cell wall/membrane/envelope biogenesis	5117509	5118633	72	-2.21	2.10e-5

plu4384	CDS	dltA	D-alanine-activating enzyme (DAE) (D-alanine-D-alanyl carrier protein ligase) (DCL)	COG1020	Q	Secondary metabolites biosynthesis, transport and catabolism	5118630	5120093	90	-1.68	1.13e-3
plu4393	CDS	-	conserved hypothetical protein	COG0823	U	Intracellular trafficking, secretion, and vesicular transport	5131421	5131987	117	-2.40	1.95e-8
plu4418	CDS	-	putative pyrrolidone-carboxylate peptidase (5-oxoprolyl-peptidase) (Pyroglutamyl-peptidase I) (PGP-I) (Pyrase)	COG2039	O	Posttranslational modification, protein turnover, chaperones	5166814	5167470	104	-1.71	3.63e-4
plu4419	CDS	-	putative exported protein	COG3485	Q	Secondary metabolites biosynthesis, transport and catabolism	5167715	5168548	99	-1.92	2.51e-4
plu4424	CDS	-	putative MFS transporter	COG2814	G	Carbohydrate transport and metabolism	5175988	5177184	198	-2.44	5.27e-11
plu4496	CDS	-	conserved hypothetical protein	COG2509	R	General function prediction only	5252293	5253813	179	-3.01	3.92e-13
pluCDS52537	CDS	-	-	COG3335	X	Mobilome: prophages, transposons	5253759	5254148	136	-3.11	2.35e-12
plu4502	CDS	-	-	COG0023	J	Translation, ribosomal structure and biogenesis	5259368	5259619	1.21e+4	-1.10	3.25e-6
plu4523	CDS	deaD	inducible ATP-independent RNA helicase	COG0513	L	Replication, recombination and repair	5281393	5283327	4394	-1.15	6.34e-7
plu4540	CDS	obgE	DNA-binding GTPase involved in cell partioning; multicopy suppresssor of ftsJ(rrmJ) (by similarity)	COG0536	DL	Cell cycle control, cell division, chromosome partitioning, Replication, recombination and repair	5302270	5303445	1651	-1.08	3.63e-5
**plu4558#**	**CDS**	**ecfJ**	**Function unknown; rpoE regulon; periplasmic protein; expressed protein (by similarity)**	**COG3054**	**R**	**General function prediction only**	**5321428**	**5321982**	**1426**	**-1.21**	**1.66e-5**
plu4570	CDS	rplI	50S ribosomal subunit protein L9	COG0359	J	Translation, ribosomal structure and biogenesis	5336224	5336676	8280	-1.02	1.33e-4
plutRNA5359	tRNA	-	transfert RNA-Gly			Unclassified	5359645	5359720	980	-1.09	3.29e-4
plutRNA5359	tRNA	-	transfert RNA-Gly			Unclassified	5359917	5359992	378	-1.18	5.46e-4
plu4628	CDS	-	putative L-2,4-diaminobutyrate decarboxylase	COG0076	E	Amino acid transport and metabolism	5394901	5396445	104	-1.46	2.17e-4
plu4629	CDS	-	-	COG3486	Q	Secondary metabolites biosynthesis, transport and catabolism	5396476	5397777	66	-1.49	2.28e-3
plu4630	CDS	-	putative rhizobactin and aerobactin siderophores biosynthesis proteins RhsF and LucC	COG1670	JO	Translation, ribosomal structure and biogenesis, Posttranslational modification, protein turnover, chaperones	5397777	5400194	184	-1.07	4.18e-3
plu4668	CDS	ilvC	ketol-acid reductoisomerase (acetohydroxy-acidisomeroreductase) (alpha-keto-beta-hydroxylacil reductoisomerase)	COG0059	EH	Amino acid transport and metabolism, Coenzyme transport and metabolism	5443372	5444850	5347	-2.14	6.40e-24
**plu4748#**	**CDS**	**-**	**-**			**Unclassified**	**5528419**	**5529000**	**1453**	**-1.93**	**7.79e-13**
plu4758	CDS	rpmE	50S ribosomal subunit protein L31	COG0254	J	Translation, ribosomal structure and biogenesis	5539633	5539848	8211	-1.02	3.71e-5
plu4844	CDS	ppyS	ketosynthase responsible for production of pyrone, signaling molecule causing Photorhabdus cell clumping	COG0332	I	Lipid transport and metabolism	5617384	5618445	18	-2.18	2.94e-3
plu4873	CDS	-	conserved hypothetical protein			Unclassified	5645829	5646260	576	-1.19	3.39e-4
plu4892	CDS	-	-	COG0500	QR	Secondary metabolites biosynthesis, transport and catabolism, General function prediction only	5665879	5666832	36	-2.58	9.73e-5

Supplementary Table 3C: List of 237 genes overexpressed in *P. luminescens* TT01 between LB culture and LB+polyB culture (logarithmic phase, FDR≤0.005, abs(log2FC)≥1)
# indicates the genes belonging to the PhoP regulon in LB broth.
indicates the genes belonging to the core PhoP regulon
* ratio LB+polyB/LB
Colors highlight some functional categories:
components of secretion system (33 genes)
insecticidal toxins (9 genes)
transporter (25 genes)
extracellular enzymes

Label	Type	Name	Product	COG_ID	COG_classID	COGClassDescription	Begin	End	normalized average read count	log2 fold change*	adjusted pvalue (FDR)
plu0052	CDS	asnA	Aspartate--ammonia ligase (Asparagine synthetase A)	COG2502	E	Amino acid transport and metabolism	46030	47022	637	1.92	1.04e-9
plu0072	CDS	yjcG	-	COG4147	C	Energy production and conversion	69280	70929	370	1.41	3.72e-5
plu0073	CDS	yjcH	-	COG3162	S	Function unknown	70926	71237	34	2.02	6.47e-4
**plu0074#**	**CDS**	**acs**	**acetyl-coenzyme A synthetase (acetate--COA ligase) (acyl-activating enzyme)**	**COG0365**	**I**	**Lipid transport and metabolism**	**71302**	**73257**	**1315**	**1.92**	**3.29e-14**
plu0082	CDS	-	conserved hypothetical protein	COG5572	S	Function unknown	78376	78669	174	1.22	4.44e-3
plu0100	CDS	pckA	phosphoenolpyruvate carboxykinase [ATP]	COG1866	C	Energy production and conversion	96415	98034	1188	1.13	1.24e-5
plu0112	CDS	cynS	cyanate lyase (cyanate hydrolase) (cyanase)	COG1513	P	Inorganic ion transport and metabolism	115374	115844	157	1.23	4.63e-3
plu0121	CDS	uspA	universal stress protein A	COG0589	T	Signal transduction mechanisms	126961	127392	1033	1.11	3.98e-5
plu0208	CDS	feoB	ferrous iron transport protein B	COG0370	P	Inorganic ion transport and metabolism	211862	214174	703	1.36	1.84e-5
plu0209	CDS	feoA	ferrous iron transport protein A	COG1918	P	Inorganic ion transport and metabolism	214227	214460	195	1.56	1.63e-4
plu0224	CDS	-	conserved hypothetical protein			Unclassified	229778	230323	189	2.83	4.45e-14
plu0225	CDS	-	Putative TpsA-related protein	COG3210	U	Intracellular trafficking, secretion, and vesicular transport	230329	235488	737	3.62	7.75e-35
plu0226	CDS	-	TpsB protein	COG2831	U	Intracellular trafficking, secretion, and vesicular transport	235518	237197	176	4.53	3.48e-25
plu0228	CDS	-	conserved hypothetical protein	COG1807	M	Cell wall/membrane/envelope biogenesis	237589	239052	472	1.16	6.75e-4
plu0300	CDS	dppA	Periplasmic dipeptide transport protein precursor DppA	COG0747	E	Amino acid transport and metabolism	317950	319557	852	2.15	7.06e-16
plu0301	CDS	dppB	Dipeptide transport system permease protein DppB	COG0601	EP	Amino acid transport and metabolism, Inorganic ion transport and metabolism	319676	320695	249	1.99	1.21e-8
plu0302	CDS	dppC	Dipeptide transport system permease protein DppC	COG1173	EP	Amino acid transport and metabolism, Inorganic ion transport and metabolism	320706	321605	345	1.80	2.56e-8
plu0303	CDS	dppD	Dipeptide transport ATP-binding protein dppD	COG0444	EP	Amino acid transport and metabolism, Inorganic ion transport and metabolism	321618	322598	269	2.00	9.47e-9
plu0304	CDS	dppF	Dipeptide transport ATP-binding protein dppF	COG4608	E	Amino acid transport and metabolism	322595	323614	414	1.33	7.85e-5
plu0418	CDS	-	Major structural subunit MrpA of Proteus mirabilis	COG3539	N	Cell motility	447141	447707	210	1.34	1.66e-4
plu0441	CDS	-	conserved hypothetical protein	COG4683	S	Function unknown	472833	473132	169	1.33	3.15e-3
plu0515	CDS	tcaC	B component of insecticidal toxin complex, protein TcaC	COG3209	R	General function prediction only	567083	571540	381	1.37	2.78e-5
plu0525	CDS	-	Similar to probable acetyltransferase	COG0456	J	Translation, ribosomal structure and biogenesis	582031	582573	101	1.97	2.08e-5
plu0526	CDS	-	conserved hypothetical protein			Unclassified	582534	583718	96	2.95	5.10e-10
pluCDS05837	CDS	-	-			Unclassified	583732	584238	153	1.48	6.70e-4
plu0527	CDS	-	conserved hypothetical protein	COG3220	S	Function unknown	584213	584992	151	2.89	2.19e-12
plu0528	CDS	-	Conserved hypothetical protein	COG3220	S	Function unknown	584996	586072	419	3.69	1.42e-29
plu0628	CDS	ygdB	-			Unclassified	712917	713402	181	2.21	5.10e-8
plu0634	CDS	-	Toxin secretion ABC transporter protein, HlyB family	COG2274	V	Defense mechanisms	726383	728518	2133	3.69	2.64e-46
plu0635	CDS	-	HlyD family secretion protein	COG0845	MV	Cell wall/membrane/envelope biogenesis, Defense mechanisms	728660	730075	589	3.85	9.48e-36
plu0636	CDS	-	conserved hypothetical protein			Unclassified	730185	730706	331	3.38	1.87e-23
plu0637	CDS	-	conserved hypothetical protein	COG0722	E	Amino acid transport and metabolism	730755	731276	640	3.68	2.52e-35
plu0638	CDS	-	conserved hypothetical protein			Unclassified	731335	731856	530	3.60	4.90e-31
plu0639	CDS	-	conserved hypothetical protein			Unclassified	731915	732445	698	3.72	7.24e-37
plu0640	CDS	-	Conserved hypothetical protein	COG5000	T	Signal transduction mechanisms	732504	733025	1231	3.78	1.83e-45
plu0641	CDS	-	conserved hypothetical protein			Unclassified	733074	733595	461	3.93	1.02e-35
plu0642	CDS	-	conserved hypothetical protein			Unclassified	733644	734165	1125	3.93	7.50e-48
plu0643	CDS	-	Putative transmembrane protein. The central region presents some similarities with proprotein convertase.	COG1404	O	Posttranslational modification, protein turnover, chaperones	734216	740587	7912	3.64	2.42e-54
**plu0691##**	**CDS**	**cutF**	**Copper homeostasis protein CutF precursor**	**COG3015**	**MV**	**Cell wall/membrane/envelope biogenesis, Defense mechanisms**	**794390**	**795061**	**3705**	**1.60**	**2.51e-10**
plu0824	CDS	-	Some similarities with gp31 protein of Bacteriophage			Unclassified	955418	956575	122	1.27	4.46e-3
plu0842	CDS	speD	S-adenosylmethionine decarboxylase proenzyme	COG1586	E	Amino acid transport and metabolism	973875	974669	644	1.43	8.83e-7
plu0843	CDS	speE	spermidine synthase (putrescine aminopropyltransferase)	COG0421	E	Amino acid transport and metabolism	974708	975577	948	2.02	8.62e-14
plu0884	CDS	-	conserved hypothetical protein	COG3266	D	Cell cycle control, cell division, chromosome partitioning	1012869	1014542	2543	1.33	5.35e-8
plu0885	CDS	-	putative pyocin S3 immunity protein			Unclassified	1014542	1014994	3375	1.20	1.07e-7
plu0886	CDS	-	Putative pyocin S3 immunity protein			Unclassified	1015076	1015528	551	1.21	1.53e-5
plu0887	CDS	-	C-terminal region of klebicin B, pyocin S2 and the killer protein of pyocin S1	COG4104	U	Intracellular trafficking, secretion, and vesicular transport	1015625	1016074	430	1.34	6.28e-6
plu0888	CDS	-	putative colicin / pyocin immunity protein			Unclassified	1016082	1016333	713	1.46	3.60e-8
plu0902	CDS	hemL	glutamate-1-semialdehyde 2,1-aminomutase	COG0001	H	Coenzyme transport and metabolism	1036929	1038212	1225	1.45	1.07e-7
plu0903	CDS	-	conserved hypothetical protein	COG0657	I	Lipid transport and metabolism	1038263	1039048	862	1.85	1.47e-10
plu0932	CDS	-	hypothetical protein	COG1708	R	General function prediction only	1073495	1074538	152	4.07	8.51e-22
plu0933	CDS	-	Conserved hypothetical protein			Unclassified	1074544	1075578	60	4.50	5.89e-15
plu0934	CDS	-	-	COG0483	G	Carbohydrate transport and metabolism	1075575	1076420	24	4.18	6.00e-8
plu0935	CDS	-	Putative biotin synthase-related enzyme	COG2516	R	General function prediction only	1076423	1077556	48	3.10	2.60e-8
plu0961	CDS	tcdB1	B component of insecticidal toxin complex, protein TcdB1	COG3209	R	General function prediction only	1103029	1107459	212	1.80	6.76e-7
plu0962	CDS	tcdA1	A component of insecticidal toxin complex, protein TcdA1	COG0497	L	Replication, recombination and repair	1107512	1115089	423	2.00	3.31e-11
plu0969	CDS	tcdB2	B component of insecticidal toxin complex, protein TcdB2	COG3209	R	General function prediction only	1132074	1136501	105	2.70	7.36e-10
plu0970	CDS	tcdA2	A component of insecticidal toxin complex, protein TcdA1	COG0497	L	Replication, recombination and repair	1136558	1143892	264	3.48	1.30e-23
plu0973	CDS	-	putative di-tripeptide transporter protein YhiP of Escherichia coli	COG3104	E	Amino acid transport and metabolism	1152675	1154144	310	1.97	1.72e-10
plu1010	CDS	-	hypothetical protein			Unclassified	1195389	1195637	81	1.58	8.97e-4
plu1012	CDS	-	conserved hypothetical protein	COG5441	S	Function unknown	1196502	1197746	694	1.16	2.47e-5

plu1149	CDS	-	CdiA protein (TpsA-related family); presence of the VENN motif and DUF638 domain	COG3210	U	Intracellular trafficking, secretion, and vesicular transport	1327789	1336602	7344	2.53	3.65e-28
plu1150	CDS	-	CdiB protein (TpsB family)	COG2831	U	Intracellular trafficking, secretion, and vesicular transport	1336651	1338315	2726	2.33	2.52e-23
plu1192	CDS	fadE	-	COG1960	I	Lipid transport and metabolism	1374488	1376923	128	1.46	1.24e-3
plu1234	CDS	-	putative exported protein	COG0584	I	Lipid transport and metabolism	1426574	1427470	60	1.75	9.01e-4
plu1266	CDS	yfiA	Highly similar to probable sigma-54 modulation protein YfiA of Escherichia coli	COG1544	J	Translation, ribosomal structure and biogenesis	1466407	1466769	92	1.95	3.37e-5
plu1306	CDS	gltJ	Glutamate/aspartate transport system permease protein GltJ	COG0765	E	Amino acid transport and metabolism	1514978	1515718	114	1.67	4.05e-4
plu1307	CDS	gltI	Glutamate/aspartate transport system permease protein GltI	COG0834	ET	Amino acid transport and metabolism, Signal transduction mechanisms	1515851	1516744	579	2.04	1.03e-12
pluCDS16291	CDS	-	-			Unclassified	1629184	1629867	697	2.26	5.21e-18
plu1367	CDS	-	CdiA protein (TpsA-related family); presence of the VENN motif and DUF638 domain	COG3210	U	Intracellular trafficking, secretion, and vesicular transport	1629806	1638691	3391	2.92	5.98e-35
plu1368	CDS	-	CdiB protein (TpsB family)	COG2831	U	Intracellular trafficking, secretion, and vesicular transport	1638740	1640404	495	3.01	2.76e-24
plu1419	CDS	kdpB	potassium-transporting ATPase B chain	COG2216	P	Inorganic ion transport and metabolism	1704464	1706530	40	1.82	6.40e-4
plu1420	CDS	kdpA	potassium-transporting ATPase A chain	COG2060	P	Inorganic ion transport and metabolism	1706551	1708257	72	1.85	7.93e-5
plu1515	CDS	-	Similar to MccB protein of Escherichia coli, involved in the production of microcin C7	COG0476	H	Coenzyme transport and metabolism	1814537	1815595	134	1.99	1.19e-6
**plu1579##**	**CDS**	**-**	**putative bacitracin transport permease YbjG of Escherichia coli**	**COG0671**	**I**	**Lipid transport and metabolism**	**1888106**	**1888714**	**741**	**1.82**	**2.45e-10**
plu1592	CDS	cspD	Cold shock-like protein CspD	COG1278	K	Transcription	1899373	1899594	192	1.79	3.02e-6
plu1694	CDS	-	conserved hypothetical protein			Unclassified	2021795	2023357	11	2.79	1.69e-3
plu1697	CDS	-	conserved hypothetical protein	COG3422	S	Function unknown	2025763	2028669	28	2.00	2.12e-3
plu1698	CDS	-	conserved hypothetical protein	COG3299	X	Mobilome: prophages, transposons	2028666	2031368	30	1.95	2.42e-3
plu1706	CDS	-	conserved hypothetical protein	COG3497	X	Mobilome: prophages, transposons	2036552	2038036	265	3.50	2.37e-22
plu1707	CDS	-	conserved hypothetical protein	COG3497	X	Mobilome: prophages, transposons	2038048	2039124	148	3.92	4.44e-20
plu1708	CDS	-	conserved hypothetical protein	COG0399	M	Cell wall/membrane/envelope biogenesis	2039178	2039627	55	4.24	4.78e-14
plu1780	CDS	mgsA	methylglyoxal synthase	COG1803	G	Carbohydrate transport and metabolism	2125558	2126016	53	1.82	1.66e-3
plu1808	CDS	-	hypothetical protein			Unclassified	2156934	2157293	189	1.32	1.16e-3
plu1813	CDS	-	putative di-tripeptide ABC transporter	COG3104	E	Amino acid transport and metabolism	2159566	2161104	120	2.50	3.71e-9
plu1864	CDS	-	Truncated gene. Putative phosphoenolpyruvate phosphomutase	COG0615	M	Cell wall/membrane/envelope biogenesis	2210011	2210184	98	3.96	3.15e-17
plu1865	CDS	-	Truncated gene. Putative phosphoenolpyruvate phosphomutase	COG2513	G	Carbohydrate transport and metabolism	2210256	2210996	57	2.90	1.15e-8
plu1866	CDS	-	Similar to nitrilotriacetate monooxygenase component A	COG2141	HR	Coenzyme transport and metabolism, General function prediction only	2211178	2212509	73	3.57	9.74e-12
plu1867	CDS	-	Truncated gene, similar to N-terminal of homocitrate synthase	COG0119	E	Amino acid transport and metabolism	2212546	2213232	61	2.92	1.01e-8
plu1868	CDS	-	Truncated gene, similar to C-terminal of homocitrate synthase	COG0119	E	Amino acid transport and metabolism	2213239	2213682	38	2.84	1.00e-6
plu1869	CDS	-	Similar to large subunit of 3-isopropylmalate dehydratase, large subunit	COG0065	E	Amino acid transport and metabolism	2213679	2214920	151	1.92	7.66e-7
plu1870	CDS	-	Similar to 3-isopropylmalate dehydratase, small subunit (leuD-2)	COG0066	E	Amino acid transport and metabolism	2214913	2215470	43	1.62	3.58e-3
plu1871	CDS	-	Putative methyltransferases	COG2226	H	Coenzyme transport and metabolism	2215467	2216252	53	1.80	8.09e-4
plu1882	CDS	sylC	Similar to non-ribosomal peptide synthetase (NRPS)			Unclassified	2240985	2242391	213	1.24	1.22e-3
plu1957	CDS	putA	proline dehydrogenase (Proline oxidase) / delta-1-pyrroline-5-carboxylate dehydrogenase (p5c dehydrogenase)	COG0506	E	Amino acid transport and metabolism	2322639	2326619	1675	1.33	1.26e-7
plu1958	CDS	putP	Sodium/proline symporter (Proline permease)	COG0591	E	Amino acid transport and metabolism	2327106	2328590	3142	1.03	3.69e-5
plu1975	CDS	-	putative xenobiotic reductase B and NAD(P)H-dependent 2-cyclohexen-1-one reductase homolog	COG1902	C	Energy production and conversion	2348123	2349178	296	2.23	5.54e-11
plu1979	CDS	sgcA	Putative phosphotransferase enzyme II, A component SgcA	COG1762	GT	Carbohydrate transport and metabolism, Signal transduction mechanisms	2351773	2352216	284	1.04	1.22e-3
plu1992	CDS	fruB	PTS system, fructose-specific IIA/FPr component	COG1925	TG	Signal transduction mechanisms, Carbohydrate transport and metabolism	2362665	2363792	488	3.37	4.01e-28
plu1993	CDS	fruA	PTS system, fructose-specific IIBC component	COG1299	G	Carbohydrate transport and metabolism	2363862	2365532	303	2.50	1.00e-13
plu1996	CDS	yhfX	-	COG3457	E	Amino acid transport and metabolism	2367087	2368265	698	1.50	4.19e-8
plu2133	CDS	yeaY	-	COG3065	M	Cell wall/membrane/envelope biogenesis	2516428	2517006	877	1.24	1.71e-5
plu2153	CDS	-	conserved hypothetical protein	COG1376	M	Cell wall/membrane/envelope biogenesis	2538015	2538377	84	3.37	9.33e-13
plu2191	CDS	-	Similarity with diaminobutyric acid aminotransferase	COG0160	E	Amino acid transport and metabolism	2576697	2580116	3267	3.51	2.40e-44
plu2192	CDS	-	Similar to molybdopterin and thiamine biosynthesis protein	COG0476	H	Coenzyme transport and metabolism	2580119	2581237	799	3.12	3.48e-28
plu2193	CDS	-	Putative gamma-butyrobetaine,2-oxoglutarate	COG2175	Q	Secondary metabolites biosynthesis, transport and catabolism	2581248	2582255	753	3.09	2.51e-27
plu2194	CDS	-	Similar to acetyltransferase	COG0456	J	Translation, ribosomal structure and biogenesis	2582304	2582744	389	3.27	1.45e-23
plu2195	CDS	-	Hypothetical protein	COG2843	M	Cell wall/membrane/envelope biogenesis	2582780	2583016	126	3.16	9.62e-13
plu2196	CDS	-	Similar to capsule biosynthesis protein	COG2843	M	Cell wall/membrane/envelope biogenesis	2582988	2583881	543	3.17	3.48e-25
plu2197	CDS	-	Similar to NikS protein involved in the biosynthesis of the peptidyl nucleoside antibiotic nikkomycin	COG0026	F	Nucleotide transport and metabolism	2583890	2585077	631	3.06	1.90e-25
plu2198	CDS	-	Similar to the phosphoribosylamine--glycine ligase	COG0151	F	Nucleotide transport and metabolism	2585118	2586380	558	3.02	2.31e-24
plu2199	CDS	-	Similar to permeases of the major facilitator superfamily	COG2271	G	Carbohydrate transport and metabolism	2586407	2587792	464	2.79	2.14e-20
plu2200	CDS	-	Similar to glutamyl-tRNA(Gln) amidotransferase chain A	COG0154	J	Translation, ribosomal structure and biogenesis	2587792	2589135	399	2.41	9.35e-15
plu2231	CDS	ynfD	-			Unclassified	2624343	2624606	695	1.37	1.47e-6
plu2232	CDS	ynfB	-	COG1508	K	Transcription	2624622	2624984	679	1.33	3.62e-6
plu2238	CDS	attM	NAHL lactonase ; Zn-metallohydrolase family	COG0491	R	General function prediction only	2632964	2633734	59	1.57	1.38e-3
plu2313	CDS	-	Lipase	COG2931	Q	Secondary metabolites biosynthesis, transport and catabolism	2709005	2710858	173	1.29	1.91e-3
plu2326	CDS	txp40	Txp40, 40 kDa insecticidal protein (previously name A24tox)			Unclassified	2744886	2745893	81	3.33	7.68e-12
plu2347	CDS	goaG	4-aminobutyrate aminotransferase (gamma-amino-N-butyrate transaminase) (GABA transaminase)	COG0160	E	Amino acid transport and metabolism	2764455	2765732	46	1.69	4.63e-3
plu2412	CDS	-	conserved hypothetical protein	COG1216	G	Carbohydrate transport and metabolism	2832866	2833933	193	1.06	1.84e-3

plu2420	CDS	-	-			Unclassified	2839044	2843954	262	1.82	6.68e-8
plu2453	CDS	-	Putative TpsA-related protein	COG3210	U	Intracellular trafficking, secretion, and vesicular transport	2880371	2885434	126	2.05	1.70e-6
**plu2480##**	**CDS**	**ail1**	**Ail protein precursor of Yersinia**	**COG3637**	**M**	**Cell wall/membrane/envelope biogenesis**	**2920701**	**2921228**	**3938**	**2.29**	**2.09e-21**
**plu2507#**	**CDS**	**ygbJ**	**Highly similar to hypothetical oxidoreductase YgbJ of Escherichia coli**	**COG2084**	**I**	**Lipid transport and metabolism**	**2951054**	**2951959**	**803**	**1.87**	**2.51e-9**
plu2508	CDS	ygbK	Function unknown; regulated by FlhDC and FliA; mutants are not defective in motility or chemotraxis (by similarity)	COG3395	S	Function unknown	2951959	2953236	607	2.05	3.54e-10
plu2509	CDS	ygbL	-	COG0235	G	Carbohydrate transport and metabolism	2953229	2953870	319	2.14	8.41e-9
plu2510	CDS	ygbM	-	COG3622	G	Carbohydrate transport and metabolism	2953889	2954668	439	2.01	4.73e-9
plu2511	CDS	ygbI	-	COG1349	KG	Transcription, Carbohydrate transport and metabolism	2954681	2955448	383	2.30	3.76e-11
**plu2512#**	**CDS**	**-**	**Putative nucleoside-diphosphate-sugar epimerase**	**COG0451**	**M**	**Cell wall/membrane/envelope biogenesis**	**2955621**	**2956586**	**441**	**2.58**	**1.68e-14**
plu2513	CDS	-	-	COG2610	GR	Carbohydrate transport and metabolism, General function prediction only	2956589	2957974	549	2.27	7.53e-13
plu2560	CDS	dadX	Alanine racemase, catabolic	COG0787	M	Cell wall/membrane/envelope biogenesis	3004014	3005102	300	1.63	3.84e-7
plu2561	CDS	dadA	D-amino acid dehydrogenase small subunit	COG0665	E	Amino acid transport and metabolism	3005109	3006422	278	1.80	2.05e-8
plu2598	CDS	ydhH	-	COG2377	M	Cell wall/membrane/envelope biogenesis	3041021	3042139	371	1.71	2.52e-7
plu2599	CDS	slyB	Outer membrane lipoprotein SlyB precursor	COG3133	M	Cell wall/membrane/envelope biogenesis	3042481	3042951	4157	1.18	2.26e-6
plu2609	CDS	-	-	COG2309	E	Amino acid transport and metabolism	3051950	3052927	167	1.19	1.10e-3
**pluCDS3053(**	**CDS**	**-**	**hypothetical protein**	**COG0469**	**G**	**Carbohydrate transport and metabolism**	**3053012**	**3053125**	**300**	**1.17**	**6.83e-5**
plu2653	CDS	nlpC	Probable lipoprotein NlpC precursor	COG0791	M	Cell wall/membrane/envelope biogenesis	3107082	3107567	119	2.01	4.15e-6
plu2654	CDS	pbgE3	4-amino-4-deoxy-L-arabinose-phosphoundecaprenol flippase, PbgE3	COG2076	V	Defense mechanisms	3107673	3108053	196	2.33	2.79e-10
**plu2655#**	**CDS**	**pbgE2**	**4-amino-4-deoxy-L-arabinose-phosphoundecaprenol flippase, PbgE2**	**COG2076**	**V**	**Defense mechanisms**	**3108050**	**3108391**	**259**	**2.03**	**3.99e-9**
**plu2656#**	**CDS**	**pbgE1**	**4-amino-4-deoxy-L-arabinose transferase, PbgE1 protein**	**COG1807**	**M**	**Cell wall/membrane/envelope biogenesis**	**3108388**	**3110049**	**1447**	**2.29**	**1.06e-18**
**plu2657##**	**CDS**	**pbgP4**	**Conserved hypothetical protein PbgP4**	**COG0726**	**GM**	**Carbohydrate transport and metabolism, Cell wall/membrane/envelope biogenesis**	**3110057**	**3110950**	**2419**	**2.40**	**5.20e-22**
**plu2658##**	**CDS**	**pbgP3**	**UDP-L-Ara4N formyltransferase ; UDP-GlcA C-4'-decarboxylase PbgP3**	**COG0223**	**J**	**Translation, ribosomal structure and biogenesis**	**3110950**	**3112932**	**6214**	**2.44**	**1.95e-24**
**plu2659##**	**CDS**	**pbgP2**	**undecaprenyl phosphate-L-Ara4FN transferase PbgP2**	**COG0463**	**M**	**Cell wall/membrane/envelope biogenesis**	**3112933**	**3113910**	**1623**	**2.49**	**1.84e-22**
**plu2660##**	**CDS**	**pbgP1**	**UDP-4-amino-4-deoxy-L-arabinose--oxoglutarate aminotransferase PbgP1**	**COG0399**	**M**	**Cell wall/membrane/envelope biogenesis**	**3113911**	**3115056**	**2190**	**2.52**	**8.88e-24**
plu2689	CDS	ftnA	nonheme ferritin 1	COG1528	P	Inorganic ion transport and metabolism	3191952	3192455	2640	1.01	5.37e-6
plu2691	CDS	pip	proline iminopeptidase (PIP)	COG0596	HR	Coenzyme transport and metabolism, General function prediction only	3193177	3194130	1359	1.89	1.29e-15
plu2692	CDS	yebF	-			Unclassified	3194204	3194578	433	1.68	1.86e-8
plu2704	CDS	-	-	COG0846	O	Posttranslational modification, protein turnover, chaperones	3204991	3208869	309	1.16	1.53e-3
plu2779	CDS	yegP	-	COG3422	S	Function unknown	3297706	3298050	157	1.63	7.26e-5
**plu2784#**	**CDS**	**pagP**	**LipidA acyltransferase PagP**			**Unclassified**	**3305063**	**3305686**	**201**	**1.80**	**2.10e-6**
**plu2807#**	**CDS**	**phoP**	**Two-component response regulator PhoP**	**COG0745**	**TK**	**Signal transduction mechanisms, Transcription**	**3332475**	**3333143**	**302**	**1.49**	**5.42e-5**
plu2808	CDS	phoQ	Two-component sensor kinase PhoQ	COG0642	T	Signal transduction mechanisms	3333163	3334626	354	1.37	5.97e-5
plu2842	CDS	-	conserved hypothetical protein	COG0702	R	General function prediction only	3373241	3373873	304	1.37	2.11e-5
plu3067	CDS	-	conserved hypothetical protein	COG0654	HC	Coenzyme transport and metabolism, Energy production and conversion	3575125	3575667	1508	2.22	1.90e-17
plu3102	CDS	-	putative methyltransferase	COG2226	H	Coenzyme transport and metabolism	3621255	3622079	242	1.82	7.57e-8
plu3103	CDS	-	putative cysteine desulfurase (NifS protein homolog)	COG1104	E	Amino acid transport and metabolism	3622091	3623257	190	1.81	2.08e-6
plu3104	CDS	-	conserved hypothetical protein			Unclassified	3623257	3624141	265	2.46	8.03e-13
plu3107	CDS	astB	succinylarginine dihydrolase	COG3724	E	Amino acid transport and metabolism	3627587	3628930	43	1.87	7.58e-4
plu3108	CDS	astD	succinylglutamic semialdehyde dehydrogenase	COG1012	C	Energy production and conversion	3628981	3630456	109	2.21	4.99e-7
plu3109	CDS	astA	arginine N-succinyltransferase (AOST)	COG3138	E	Amino acid transport and metabolism	3630459	3631490	70	2.47	4.65e-7
plu3110	CDS	argM	succinylornithine transaminase (succinylornithine aminotransferase) (carbon starvation protein C)	COG4992	E	Amino acid transport and metabolism	3631512	3632720	102	2.75	1.29e-10
plu3120	CDS	-	Putative membrane protein	COG3209	R	General function prediction only	3639471	3643967	194	1.85	2.31e-6
plu3121	CDS	-	hypothetical protein			Unclassified	3643983	3644150	199	1.79	2.55e-6
plu3146	CDS	lsrB	ABC transporter Binding Protein (BP) LsrB	COG1879	G	Carbohydrate transport and metabolism	3709664	3710683	149	1.25	4.12e-3
plu3154	CDS	-	Hemolysin-coregulated protein (Hcp protein)	COG3157	U	Intracellular trafficking, secretion, and vesicular transport	3714422	3714901	481	1.40	2.19e-7
plu3164	CDS	yfcG	-	COG0625	O	Posttranslational modification, protein turnover, chaperones	3724793	3725413	511	1.08	1.54e-3
plu3201	CDS	fadI	Beta-ketoacyl-CoA thiolase, anaerobic beta-oxidation complex II; in FadR regulon (by similarity)	COG0183	I	Lipid transport and metabolism	3766624	3767934	152	1.41	1.16e-3
plu3287	CDS	treC	Trehalose-6-phosphate hydrolase (alpha,alpha-phosphotrehalase)	COG0366	G	Carbohydrate transport and metabolism	3901245	3902906	1241	1.88	1.30e-12
**plu3288#**	**CDS**	**treB**	**PTS system, trehalose-specific IIBC component (EIIBC-TRE) (trehalose-permease IIBC component) (phosphotransferase enzyme II, BC component) (EII-TRE)**	**COG1263**	**G**	**Carbohydrate transport and metabolism**	**3903006**	**3904427**	**643**	**2.21**	**2.59e-14**
plu3507	CDS	-	Putative major fimbrial sub-unit protein	COG3539	N	Cell motility	4099071	4099475	280	6.26	9.09e-47
plu3508	CDS	-	putative Fimbrial regulatory gene			Unclassified	4100136	4100411	68	4.61	2.92e-17
plu3509	CDS	-	conserved hypothetical protein	COG0456	J	Translation, ribosomal structure and biogenesis	4100619	4101074	166	1.81	3.70e-6
plu3539	CDS	prpE	Propionate--CoA ligase (Propionyl-CoA synthetase)	COG0365	I	Lipid transport and metabolism	4168817	4170703	213	1.18	4.20e-4
plu3540	CDS	prpD	2-methylcitrate dehydratase	COG2079	G	Carbohydrate transport and metabolism	4170776	4172227	254	1.43	3.21e-6
plu3541	CDS	prpC	2-methylcitrate synthase (Methylcitrate synthase) (Citrate synthase 2)	COG0372	C	Energy production and conversion	4172282	4173448	209	1.62	5.93e-7
plu3542	CDS	prpB	Probable methylisocitrate lyase (2-methylisocitrate lyase)	COG2513	G	Carbohydrate transport and metabolism	4173509	4174399	200	1.93	2.57e-9
plu3543	CDS	prpR	Propionate catabolism operon regulatory protein	COG3829	KT	Transcription, Signal transduction mechanisms	4174732	4176399	373	1.46	1.71e-5

plu3569	CDS	-	TpsB protein	COG2831	U	Intracellular trafficking, secretion, and vesicular transport	4208442	4210121	145	2.89	2.68e-12
plu3593	CDS	-	conserved hypothetical protein	COG3210	U	Intracellular trafficking, secretion, and vesicular transport	4235193	4236086	25	3.34	7.30e-7
plu3594	CDS	-	Putative TpsA-related protein	COG3210	U	Intracellular trafficking, secretion, and vesicular transport	4236073	4242480	4387	3.58	7.76e-50
plu3595	CDS	-	-	COG2831	U	Intracellular trafficking, secretion, and vesicular transport	4242432	4242791	384	3.48	8.52e-26
plu3596	CDS	gcvP	glycine dehydrogenase [decarboxylating] (glycine decarboxylase) (glycine cleavage system P-protein)	COG0403	E	Amino acid transport and metabolism	4243413	4246289	1528	1.76	7.96e-13
plu3597	CDS	gcvH	glycine cleavage system H protein	COG0509	E	Amino acid transport and metabolism	4246377	4246769	828	1.12	6.57e-5
plu3598	CDS	gcvT	aminomethyltransferase (glycine cleavage system T protein)	COG0404	E	Amino acid transport and metabolism	4246891	4247985	973	1.45	3.70e-8
plu3625	CDS	-	hypothetical protein			Unclassified	4277274	4277642	324	1.20	4.39e-4
plu3626	CDS	-	-	COG0472	M	Cell wall/membrane/envelope biogenesis	4277690	4278037	134	1.53	3.16e-4
plu3627	CDS	-	putative transcription regulator.	COG3655	K	Transcription	4278307	4278528	295	1.14	3.42e-4
plu3628	CDS	-	conserved hypothetical protein	COG1086	MO	Cell wall/membrane/envelope biogenesis, Posttranslational modification, protein turnover, chaperones	4278537	4279115	843	1.53	3.98e-8
plu3630	CDS	-	Conserved hypothetical protein	COG3539	N	Cell motility	4280090	4280476	144	5.03	1.21e-26
pluCDS42807	CDS	-	hypothetical protein			Unclassified	4280440	4280703	115	5.30	4.11e-25
plu3631	CDS	-	putative fimbrial chaperone YcbR precursor of Escherichia coli	COG3121	W	Extracellular structures	4280806	4281522	98	4.73	9.09e-20
plu3632	CDS	-	-	COG3539	N	Cell motility	4281533	4281748	30	3.98	1.67e-9
plu3633	CDS	-	-	COG3539	N	Cell motility	4281700	4281996	28	4.22	1.17e-9
plu3634	CDS	-	putative the N-terminal region of outer membrane usher protein precursor	COG3188	NW	Cell motility, Extracellular structures	4282079	4283065	26	3.57	2.59e-7
plu3635	CDS	-	conserved hypothetical protein			Unclassified	4283163	4283507	7.42	3.30	7.20e-4
plu3718	CDS	-	CdiA protein (TpsA-related family); presence of the VENN motif and DUF638 domain	COG3210	U	Intracellular trafficking, secretion, and vesicular transport	4374057	4383140	1683	3.27	4.86e-39
plu3719	CDS	-	CdiB protein (TpsB family)	COG2831	U	Intracellular trafficking, secretion, and vesicular transport	4383189	4384853	198	3.13	4.07e-17
plu3725	CDS	abgB	aminobenzoyl-glutamate utilization protein B	COG1473	R	General function prediction only	4391036	4392484	34	1.98	1.00e-3
plu3727	CDS	abgR	Putative transcriptional regulator of abgABT operon	COG0583	K	Transcription	4394074	4394973	94	2.23	4.75e-7
plu3740	CDS	-	putative sodium:alanine symporter and other amino acid transport proteins	COG1115	E	Amino acid transport and metabolism	4410741	4412108	670	1.36	6.34e-7
plu3767	CDS	sctN	Type III secretion component protein SctN	COG1157	NU	Cell motility, Intracellular trafficking, secretion, and vesicular transport	4434397	4435719	79	2.57	5.22e-7
plu3771	CDS	sctR	Type III secretion component protein SctR	COG4790	U	Intracellular trafficking, secretion, and vesicular transport	4438248	4438901	7.69	3.86	4.81e-4
plu3908	CDS	ggt	gamma-glutamyltranspeptidase	COG0405	E	Amino acid transport and metabolism	4579987	4581747	895	1.22	8.07e-6
plu3991	CDS	sstT	Na+/serine (threonine) symporter	COG3633	E	Amino acid transport and metabolism	4678384	4679631	234	1.19	2.34e-3
plu4084	CDS	-	putative transport protein and multidrug resistance protein like bicyclomycin resistance protein	COG2814	G	Carbohydrate transport and metabolism	4770938	4772125	249	1.36	2.61e-4
plu4168	CDS	tccB1	A component of insecticidal toxin complex (part 2), protein TccB1	COG1538	M	Cell wall/membrane/envelope biogenesis	4869362	4874056	737	1.04	4.61e-4
plu4169	CDS	tccA1	A component of insecticidal toxin complex (part 1), protein TccB1	COG3206	M	Cell wall/membrane/envelope biogenesis	4874151	4877051	482	1.49	1.38e-6
plu4172	CDS	-	putative pyocin S3 immunity protein and to Photorhabdus luminescens proteins			Unclassified	4878614	4879081	305	1.00	4.20e-3
**plu4210##**	**CDS**	**-**	**Conserved hypothetical protein with SANT/Myb DNA-binding domain (IPR001005)**	**COG0287**	**E**	**Amino acid transport and metabolism**	**4923248**	**4925854**	**554**	**1.46**	**4.75e-7**
**plu4211##**	**CDS**	**-**	**Putative Type VI secretion system effector, Hcp (IPR008514)**	**COG3157**	**U**	**Intracellular trafficking, secretion, and vesicular transport**	**4925955**	**4926446**	**1819**	**2.52**	**7.93e-26**
**plu4212##**	**CDS**	**-**	**Hypothetical protein**			**Unclassified**	**4926419**	**4926589**	**248**	**2.14**	**3.54e-10**
plu4213	CDS	-	Hypothetical protein			Unclassified	4926590	4926778	50	2.46	2.41e-6
**plu4214#**	**CDS**	**-**	**Putative type VI secretion system IcmF, C-terminal (domains IPR010623 and IPR009612)**	**COG3523**	**U**	**Intracellular trafficking, secretion, and vesicular transport**	**4927191**	**4930550**	**672**	**2.17**	**3.29e-14**
**plu4215#**	**CDS**	**-**	**Conserved hypothetical protein. Putative membrane protein.**	**COG3000**	**I**	**Lipid transport and metabolism**	**4930543**	**4931718**	**275**	**1.86**	**1.31e-7**
**plu4216#**	**CDS**	**-**	**Conserved hypothetical protein**	**COG4104**	**U**	**Intracellular trafficking, secretion, and vesicular transport**	**4931721**	**4931996**	**135**	**1.54**	**1.87e-4**
**plu4217#**	**CDS**	**-**	**Conserved hypothetical protein. Putative membrane protein**			**Unclassified**	**4932024**	**4932692**	**260**	**1.50**	**7.79e-6**
**plu4220##**	**CDS**	**-**	**Conserved hypothetical protein with SANT/Myb DNA-binding domain (IPR001005)**	**COG0287**	**E**	**Amino acid transport and metabolism**	**4934952**	**4937552**	**1320**	**1.14**	**9.94e-5**
**plu4221##**	**CDS**	**-**	**Conserved hypothetical protein**			**Unclassified**	**4937552**	**4938592**	**427**	**1.36**	**1.15e-4**
**plu4222##**	**CDS**	**-**	**Putative Type VI secretion system, RhsGE-associated Vgr protein (domain IPR006533)**	**COG3501**	**UXR**	**Intracellular trafficking, secretion, and vesicular transport, Mobilome: prophages, transposons, General function prediction only**	**4938603**	**4940981**	**907**	**1.33**	**3.63e-5**
**plu4223##**	**CDS**	**-**	**Putative type VI secretion ATPase, ClpV1 family**	**COG0542**	**O**	**Posttranslational modification, protein turnover, chaperones**	**4940978**	**4943659**	**1722**	**1.11**	**4.55e-4**
**plu4291##**	**CDS**	**-**	**conserved hypothetical protein**	**COG3577**	**R**	**General function prediction only**	**5018093**	**5019289**	**644**	**2.05**	**5.07e-12**
plu4402	CDS	fadB	Fatty oxidation complex alpha subunit [includes: enoyl-CoA hydratase; delta(3)-cis-delta(2)-trans-enoyl-CoA isomerase; 3-hydroxyacyl-CoA dehydrogenase; 3-hydroxybutyryl-CoA epimerase]	COG1024	I	Lipid transport and metabolism	5149379	5151565	386	1.54	6.21e-7
plu4403	CDS	fadA	3-ketoacyl-CoA thiolase (fatty oxidation complex beta subunit) (beta-ketothiolase) (acetyl-CoA acyltransferase)	COG0183	I	Lipid transport and metabolism	5151577	5152743	256	1.37	1.24e-4
plu4556	CDS	ytfL	-	COG1253	R	General function prediction only	5319488	5320819	253	1.32	1.50e-4
plu4601	CDS	-	-	COG3501	UXR	Intracellular trafficking, secretion, and vesicular transport, Mobilome: prophages, transposons, General function prediction only	5368573	5370549	1438	1.35	2.16e-6
plu4602	CDS	-	Conserved hypothetical protein	COG1716	T	Signal transduction mechanisms	5370546	5371037	620	1.43	4.68e-6
plu4603	CDS	-	conserved hypothetical protein			Unclassified	5371040	5371384	341	1.29	4.00e-4
plu4604	CDS	-	conserved hypothetical protein			Unclassified	5371386	5371994	338	1.40	6.45e-5
plu4605	CDS	-	Conserved hypothetical protein			Unclassified	5372018	5372740	428	1.44	5.86e-6
plu4606	CDS	-	conserved hypothetical protein	COG4104	U	Intracellular trafficking, secretion, and vesicular transport	5372743	5373006	110	1.38	2.86e-3
plu4607	CDS	-	conserved hypothetical protein			Unclassified	5373170	5373802	270	1.46	7.69e-5
plu4608	CDS	-	Conserved hypothetical protein			Unclassified	5373851	5374585	395	1.52	8.62e-7
plu4609	CDS	-	conserved hypothetical protein	COG4104	U	Intracellular trafficking, secretion, and vesicular transport	5374588	5374851	145	1.54	5.76e-4

plu4611	CDS	-	conserved hypothetical protein			Unclassified	5375668	5376402	71	1.63	1.10e-3
plu4767	CDS	glpF	glycerol uptake facilitator protein	COG0580	G	Carbohydrate transport and metabolism	5548786	5549616	373	1.07	1.84e-3
plu4774	CDS	pfkA	6-phosphofructokinase isozyme I (phosphofructokinase-1) (phosphohexokinase-1)	COG0205	G	Carbohydrate transport and metabolism	5555554	5556525	828	1.24	3.19e-5
plu4777	CDS	ynbA	-	COG0558	I	Lipid transport and metabolism	5558482	5559156	170	1.32	1.59e-3
plu4793	CDS	cpxP	periplasmic protein precursor	COG3678	O	Posttranslational modification, protein turnover, chaperones	5566624	5567118	246	2.25	1.60e-11

Supplementary Table 3D: RT-qPCR comparative analysis of 20 differentially expressed marker genes in polyB resistant clones (PolyR) cultivated in LB+polyB or in LB cultures (OD=0.3)
# indicates the genes belonging to the PhoP regulon in LB broth.
indicates the genes belonging to the core PhoP regulon.
Colors highlight some functional categories:
components of secretion system
insecticidal toxins
transporter
extracellular enzymes

Label	Type	Name	Product	COG_ID	COG_classID	COGClassDescription	log2 fold change RNA-Seq PolyR(LB+poly) vs TT01(LB)	log2 fold change RT-qPCR PolyR(LB+poly) vs TT01(LB)	log2 fold change RT-qPCR PolyR(LB) vs TT01(LB)
plu0015	CDS	pts20	Major phage tail sheath protein	COG3497	X	Mobilome: prophages, transposons	-5.87	-6.16	-3.46
plu0226	CDS	-	TpsB protein	COG2831	U	Intracellular trafficking, secretion, and vesicular transport	4.53	4.29	2.61
plu0300	CDS	dppA	Periplasmic dipeptide transport protein precursor DppA	COG0747	E	Amino acid transport and metabolism	2.15	2.36	0.3
plu0373	CDS	-	Putative Type VI secretion system effector, Hcp (IPR008514)	COG3157	U	Intracellular trafficking, secretion, and vesicular transport	-2.01	-1.74	-1.7
plu0549	CDS	-	CdiB protein (TpsB family)	COG2831	U	Intracellular trafficking, secretion, and vesicular transport	-2.43	-1.74	-1.14
**plu0691##**	**CDS**	**cutF**	**Copper homeostasis protein CutF precursor**	**COG3015**	**MV**	**Cell wall/membrane/envelope biogenesis, Defense mechanisms**	1.6	1.97	1.61
plu0969	CDS	tcdB2	B component of insecticidal toxin complex, protein TcdB2	COG3209	R	General function prediction only	2.7	2.35	0.76
plu1367	CDS	-	CdiA protein (TpsA-related family); presence of the VENN motif and DUF638 domain	COG3210	U	Intracellular trafficking, secretion, and vesicular transport	2.92	2.66	0.7
plu1382	CDS	prtS	Extracellular M4 metalloprotease precursor PrtS	COG3227	O	Posttranslational modification, protein turnover, chaperones	-2.16	-2.91	-3.25
plu1414	CDS	-	putative hydrolase	COG0561	HR	Coenzyme transport and metabolism, General function prediction only	-1.15	-0.61	-0.38
plu1516	CDS	-	Lipase	COG3675	I	Lipid transport and metabolism	-1.41	-0.92	-0.74
**plu1579##**	**CDS**	**-**	**putative bacitracin transport permease YbjG of Escherichia coli**	**COG0671**	**I**	**Lipid transport and metabolism**	1.82	1.53	0.61
plu2326	CDS	txp40	Txp40, 40 kDa insecticidal protein (previously name A24tox)			Unclassified	3.33	3.09	4.18
**plu2480##**	**CDS**	**ail1**	**Ail protein precursor of Yersinia**	**COG3637**	**M**	**Cell wall/membrane/envelope biogenesis**	2.29	1.73	1.97
**plu2660##**	**CDS**	**pbgP1**	**UDP-4-amino-4-deoxy-L-arabinose--oxoglutarate aminotransferase PbgP1**	**COG0399**	**M**	**Cell wall/membrane/envelope biogenesis**	2.52	2.10	1.57
**plu2807#**	**CDS**	**phoP**	**Two-component response regulator PhoP**	**COG0745**	**TK**	**Signal transduction mechanisms, Transcription**	1.49	1.40	0.64
plu3594	CDS	-	Putative TpsA-related protein	COG3210	U	Intracellular trafficking, secretion, and vesicular transport	3.58	3.36	2.00
plu3718	CDS	-	CdiA protein (TpsA-related family); presence of the VENN motif and DUF638 domain	COG3210	U	Intracellular trafficking, secretion, and vesicular transport	3.27	2.86	1.16
plu4194	CDS	antA	Ketoreductase AntA involved in anthraquinone biosynthesis (type II polyketide synthase)	COG1028	IQR	Lipid transport and metabolism, Secondary metabolites biosynthesis, transport and catabolism, General function prediction only	-3.8	-3.64	-4.8
**plu4211##**	**CDS**	**-**	**Putative Type VI secretion system effector, Hcp (IPR008514)**	**COG3157**	**U**	**Intracellular trafficking, secretion, and vesicular transport**	2.52	2.46	0.90

Supplementary Table 4: Bacterial strains and plasmids used in this study.

Strain or plasmid	Description	Source or reference
***P. luminescens* strains**		
**TT01**	Strain isolated from the nematode *Heterorhabditis bacteriophora* TH01 in Trinidad; wild-type (WT) form	Laboratory collection
phoP	TT01 *phoP*::cat ; *phoP* mutant	1
pbgE	TT01 *pbgE1::*Kan ; *pbgE* mutant	2
***phoP*/ pBBR1-MCS5**	*phoP* mutant carrying pBBR1-MCS5 (negative control), Gm^R	This study
phoP*/ P_{lac}-*phoPQ	*phoP* mutant carrying P_{lac}-*phoPQ* plasmid, Gm^R	1
***pbgE*/ pBBR1-MCS5**	*pbgE* mutant carrying pBBR1-MCS5 (negative control), Gm^R	This study
pbgE*/ P_{lac}-*pbgPE	*pbgE* mutant carrying P_{lac}-*pbgPE* plasmid, Gm^R	3
**TT01/ P_{pbgPE}-*gfp*[AAV]**	TT01 carrying P_{pbgPE}-*gfp*[AAV] plasmid, Kan^R	This study
**TT01/ P_{pbgPE}-*gfp*[mut3]**	TT01 carrying P_{pbgPE}-*gfp*[mut3] plasmid, Kan^R	This study
**TT01/ pPROBE-*gfp*[AAV]**	TT01 carrying pPROBE-*gfp*[AAV] plasmid, Kan^R	This study
***E. coli* strains**		
**XL1 blue MRF'**	Δ(*mcrA*)*183* Δ(*mcrCB-hsdSMR-mrr*)*173 endA1 supE44 thi-1 recA1* gyrA96 relA1 lac [*F'* proAB $lacI^qZ\Delta$M15 *Tn*10 *(Tet^r)]*	Stratagene
**WM3064**	*thrB*1004 *pro thi rpsL hsdS lacZ*ΔM15 RP4-1360 Δ(*araBAD*)567 Δ*dapA*1341::[erm pir (wt)]	4
**BL21 (DE3) pLysS**	F^- *dcm ompT hsdS*($r_B^- m_B^-$) *gal* λ(DE3) [pLysS Cam^R]	Laboratory stock
**Plasmids**		
**pBBR1-MCS5**	broad host range vector Gm^R *mob*	5
**pPROBE-*gfp*[AAV]**	Plasmid (pBBR1 replicon) containing promoter-less *gfp[*AAV] gene downstream from a multiple cloning site, Kan^R	6
**P_{pbgPE}-*gfp*[AAV]**	pPROBE with *gfp*[AAV] under the control of *pbgPE* promoter, Kan^R	this study
**pBB-KGFP**	pBB broad host range *gfp*[mut3] Kan^R	S. Köhler

**P_{pbgPE}-*gfp*[mut3]**	pPROBE with *gfp*[mut3] under the control of *pbgPE* promoter, Kan^R	this study
**P_{T7}PhoP-His**	pET28 with *phoP*(His-tag) in N-term under the control of T7 promoter; Amp^R	[7]

1 Derzelle, S. *et al.* The PhoP-PhoQ two-component regulatory system of *Photorhabdus luminescens* is essential for virulence in insects. *Journal of bacteriology* **186**, 1270-1279, doi: 10.1128/JB.186.5.1270-1279 (2004).

2 Bennett, H. P. & Clarke, D. J. The *pbgPE* operon in *Photorhabdus luminescens* is required for pathogenicity and symbiosis. *Journal of bacteriology* **187**, 77-84, doi:10.1128/JB.187.1.77-84.2005 (2005).

3 Abi Khattar, Z. *Impact de la résistance aux peptides antimicrobiens et aux composés toxiques sur les interactions bactéries-insectes : Cas des infections par Photorhabdus luminescens et Bacillus cereus* PhD thesis, Université Montpellier 2, Université Saint-Joseph de Beyrouth, (2009).

4 Paulick, A. *et al.* Two different stator systems drive a single polar flagellum in S*hewanella oneidensis* MR-1. *Molecular microbiology* **71**, 836-850, doi:10.1111/j.1365-2958.2008.06570.x (2009).

5 Kovach, M.E., Elzer, P.H., Hill, D.S., Robertson, G.T., Farris, M.A., Roop, R.M., 2nd, and Peterson, K.M. Four new derivatives of the broad-host-range cloning vector pBBR1MCS, carrying different antibiotic-resistance cassettes. *Gene* **166:** 175–176, doi:10.1016/0378-1119(95)00584-1 (1995).

6 Miller, W. G., Leveau, J. H. & Lindow, S. E. Improved gfp and inaZ broad-host-range promoter-probe vectors. *Molecular plant-microbe interactions : MPMI* **13**, 1243-1250, doi:10.1094/MPMI.2000.13.11.1243 (2000).

7 Mouammine, A. *et al.* Ail and PagC-related proteins in the entomopathogenic bacteria of *Photorhabdus* genus. *PloS one* **9**, e110060, doi:10.1371/journal.pone.0110060 (2014).

Supplementary Table 5

Primer name	Primer sequence (5' to 3')	Use
L-P_{pbgPE}-*Xba*I	GCTCTAGATTACAGTCCAGGCTTATGTATGTGCC	cloning of *pbgPE* promoter from *Photorhabdus luminescens* TT01
R-P_{pbgPE}-*Kpn*I	CAGGTACCCTATGGAAGAAAGCTATCCATAAAACACAGTCC	
L-P_{pbgPE}	TTACAGTCCAGGCTTATGTATGTGCC	EMSA analysis of *pbgPE* promoter
R-P_{pbgPE}	ATGGAAGAAAGCTATCCATAAAACACAGTCC	
L-P_{phoP}	AAACATCCGTCTGTTGCTATCC	EMSA analysis of *phoP* promoter
R-P_{phoP}	CTCTCCAGCAGGCAGTTATTG	
L-gyrB	ATACACGAAGAAGAAGGTGTTTCAG	qRT-PCR of an internal region within *gyrB* from *Photorhabdus luminescens* TT01
R-gyrB	TACCTGTCTGTTCAGTTTCTCCAAC	
L-recA	GTTCAATGGACGTTGAAACTATCTC	qRT-PCR of an internal region within *recA* from *Photorhabdus luminescens* TT01
R-recA	ATCAACACCCAACTTCTTAGCATAG	
L-cutF	GTAGATCAGACTTTTCATGGTGCTC	qRT-PCR of an internal region within *cutF* from *Photorhabdus luminescens* TT01
R-cutF	AGATTTCTGGTCGTCTGATTTTGTC	
L-ail1Pl	AGAACATTAGTGGCTTCAGTGGTAG	qRT-PCR of an internal region within *ail1* from *Photorhabdus luminescens* TT01
R-ail1Pl	ATTATCCAACTCGTAGCGGTATTTC	
L-pbgP1	GAATTCGATGTCTAAAGTTTCATGG	qRT-PCR of an internal region within *pbgP1* from *Photorhabdus luminescens* TT01
R-pbgP1	GCGAATATTCATTGATTCCAATTTA	
L-mgtE	ACTGTAGCCGATGATTTATTTGTGT	qRT-PCR of an internal region within *mgtE* from *Photorhabdus luminescens* TT01
R-mgtE	ACGTATTTTCATCTTCAACCAACTC	
L-3988	GCAACGAAAGATTGTTATGGATTAT	qRT-PCR of an internal region within *plu3988* from *Photorhabdus luminescens* TT01
R-3988	TAATGATTCCCAGAAAGAGTCGTAA	
L-plu4211	AAAGGTTCGGTGGATGTACACAAC	qRT-PCR of an internal region within *plu4211* from *Photorhabdus luminescens* TT01
R-plu4211	CGCTTTATACAGATACGGGCTAGA	
L-plu4227	GGTTTACTCGTCTTTGTTTGAGAAA	qRT-PCR of an internal region within *plu4227* from *Photorhabdus luminescens* TT01
R-plu4227	CTTTATCCAACTGTTCCACTTTTTG	

Primer name	Primer sequence (5' to 3')	Use
L-plu0015	AATTAGGTGTGAAGCCTCGTATTCT	qRT-PCR of an internal region within *plu0015* from *Photorhabdus luminescens* TT01
R-plu0015	ATCAGTTCTCGTTGACTGAAGTTTT	
L-plu0226	GTTATATCGTGATGGAGAGCAGAAG	qRT-PCR of an internal region within *plu0226* from *Photorhabdus luminescens* TT01
R-plu0226	ACCATCGGGTTAAAGGTGACATAG	
L-plu0300	GCAGAATATGCAGATAAGATGATGA	qRT-PCR of an internal region within *plu0300* from *Photorhabdus luminescens* TT01
R-plu0300	AATAGAGAAAACTAGCCGGTCAACT	
L-plu0373	GCAACCATCTTTAGTCAGGACATTA	qRT-PCR of an internal region within *plu0373* from *Photorhabdus luminescens* TT01
R-plu0373	AAATACCTCCCAGAATCTCATTTCT	
L-plu0549	AAGCAAAAATCTGAATGTCCAACT	qRT-PCR of an internal region within *plu0549* from *Photorhabdus luminescens* TT01
R-plu0549	TACCGATGTAGTGACGATGAGATAA	
L-plu0969	TGGTTGTTCTACCTGGTATTTGATT	qRT-PCR of an internal region within *plu0969* from *Photorhabdus luminescens* TT01
R-plu0969	GATACATCAGTATCTGACGGCATAA	
L-plu1367	TACACTCAAGTTGCTAATCCAACTG	qRT-PCR of an internal region within *plu1367* from *Photorhabdus luminescens* TT01
R-plu1367	ACCTGCTCCTACAGGAGAATAGTTT	
L-plu1382	TGAAGAAGCTGATTGGATTATAGGT	qRT-PCR of an internal region within *plu1382* from *Photorhabdus luminescens* TT01
R-plu1382	ACTCCACCATTGTCTCTATAAATCG	
L-plu1414	TCAAGTATGGGAAAGCAAATTGTAT	qRT-PCR of an internal region within *plu1414* from *Photorhabdus luminescens* TT01
R-plu1414	TCAATCACTCTTTTTACGATGTGAC	
L-plu1516	GGTTTGATGAGAATTTCAATGTAGG	qRT-PCR of an internal region within *plu1516* from *Photorhabdus luminescens* TT01
R-plu1516	ATTTTATCAATTGCCTCAATCAATG	
L-plu1579	GAAGGCTTCGGTTATAACTTTCTTC	qRT-PCR of an internal region within *plu1579* from *Photorhabdus luminescens* TT01
R-plu1579	AGTGAACGCCTAAATAGACTCTGG	
L-plu2326	GATTACCTGATGGCAGAATAGGTTA	qRT-PCR of an internal region within *plu2326* from *Photorhabdus luminescens* TT01
R-plu2326	GTACCAATACTTTTTGCTGCTGTCT	

**Primer name**	**Primer sequence (5' to 3')**	**Use**
L-plu2807	ATTACTATCTGGTCGAGAGCGAAC	qRT-PCR of an internal region within *plu2807* from *Photorhabdus luminescens* TT01
R-plu2807	TGGTAACATAATCATCTGCTCCTG	
L-plu3594	CCCCTATGAATATGAAGATGAACAC	qRT-PCR of an internal region within *plu3594* from *Photorhabdus luminescens* TT01
R-plu3594	TCAGTATCAATAATGTCACCACCAG	
L-plu3718	CTGACGGGGACTCATAACTCAG	qRT-PCR of an internal region within *plu3718* from *Photorhabdus luminescens* TT01
R-plu3718	TTTATTACCTGCGTTGATCAATTTT	
L-plu4194	CTTATTCTGCTTCTAAGCATGGTGT	qRT-PCR of an internal region within *plu4194* from *Photorhabdus luminescens* TT01
R-plu4194	TCTTCTACTCTTCTTTTCGCTTCTG	
